# Design and efficient synthesis of pyrazoline and isoxazole bridged indole *C*-glycoside hybrids as potential anticancer agents

**DOI:** 10.1038/s41598-020-63377-x

**Published:** 2020-04-20

**Authors:** Priti Kumari, Vishnu S. Mishra, Chintam Narayana, Ashish Khanna, Anindita Chakrabarty, Ram Sagar

**Affiliations:** 1 Department of Chemistry, School of Natural Sciences, Shiv Nadar University (SNU), NH91, Tehsil-Dadri, Gautam Buddha Nagar, Uttar Pradesh 201314 India; 2 Department of Life Sciences, School of Natural Sciences, Shiv Nadar University (SNU), NH91, Tehsil-Dadri, Gautam Buddha Nagar, Uttar Pradesh 201314 India; 3 Department of Chemistry, Institute of Science, Banaras Hindu University, Varanasi, Uttar Pradesh 221005 India

**Keywords:** Cancer, Drug discovery, Chemistry

## Abstract

*C-*glycosides are important class of molecules exhibit diverse biological activities and present as structural motif in many natural products. Two series of new pyrazoline and isoxazole bridged indole *C*-glycoside molecular hybrids (n = 36) were efficiently synthesized starting from diverse indole 3-carboxaldehydes derived α, β-unsaturated ketone derivatives of β-D-glucosyl-propan-2-one, β-D-galactosyl-propan-2-one and β-D-mannosyl-propan-2-one, reacting with hydrazine hydrate and hydroxyl amine hydrochloride in shorter reaction time (15 min) under microwave assisted condition. Anticancer activity of these newly synthesized pyrazoline and isoxazole bridged indoles *C*-glycoside hybrids were determined in details through cellular assays against MCF-7, MDA-MB-453 and MDA-MB-231 cancer cell lines. The selected library members displayed low micromolar (IC_50_ = 0.67–4.67 µM) and selective toxicity against breast cancer cell line (MCF-7). Whereas these compounds were nontoxic towards normal cell line (MCF-10A). Mechanistic studies showed that, active compounds inhibit COX-2 enzyme, which was also supported by molecular docking studies. These findings are expected to provide new leads towards anticancer drug discovery.

## Introduction

*C*-Glycosides are stable surrogates of *O*-glycosides and are often used as investigative tool in chemical biology and medicinal chemistry, in particular *C*-glycoside analogues are often used as substrate mimics for different enzymes involved in the carbohydrate metabolism^1–10^. The use of glycomimetic in the design of inhibitors has proven to be a successful approach and now constitutes one of the most attractive ways to develop new generations of effective and selective inhibitors^11^. α, β-unsaturated ketone is a central core for many significant biological compounds^12,13^. According to literature α, β-unsaturated ketone derivatives from natural and synthetic analogues exhibit diverse pharmacological activities such as anti-tuberculosis, anti-inflammatory, anticancer, antineoplastic, antibacterial, antifungal, antimalarial, antiviral, antiallergic, estrogenic^12^ and COX-2 inhibitor^13^.

**Figure 1:**
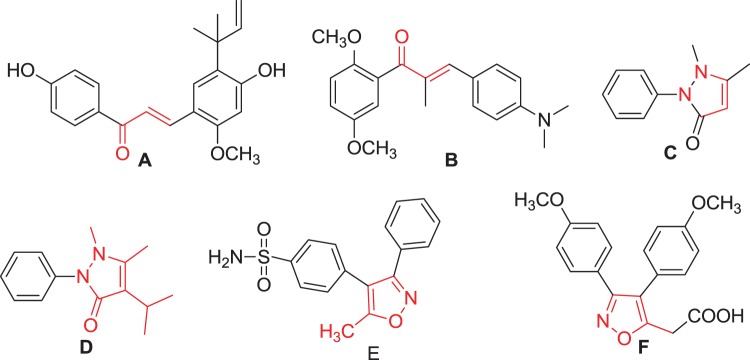
Known drug molecule with chalcone, pyrazole and isoxazole moiety: [**A**] Licochalcone A, anticancer agent. [**B**] MDL 27048, antitumor agent. [**C**] Phenazone, antipyretic agent. [**D**] Propyphenazone, anti-inflammatory agent. [**E**] Valdecoxib, COX-2 inhibitor. [**F**] Mofezolac, COX-2 inhibitor.

Pyrazoline derivatives are important biologically active heterocyclic compounds. These derivatives are the subject of many research studies due to their widespread potential biological activities such as anticancer, antioxidant, antibacterial activities^14^ and COX-2 inhibitor^15^. The most prominent compounds containing pyrazoline nucleus are ampyrone, phenazone and propyphenzone. Isoxazole heterocycle is well known for its medicinal relevance^16,17^. There are isoxazole nucleus containing drug molecules currently available in market, such as acetylsulfisoxazole, cycloserine, sulfisoxazole, and zonisamide etc. These drugs possess antimicrobial^18^, antioxidant^19,20^ tuberculostatic^21^ neurotoxic^22^, antiepileptic activities^23,24^ and COX-2 inhibitor^25^. The comparable activities are also observed in other isoxazole derivatives, captivates researcher to search for newer bioactive compounds of using this as pharmacophore. α, β-unsaturated ketone, pyrazoline and isoxazole motifs are found in many known drugs (Fig. 1).

Indole nucleus is known as privileged structure and has been reported for its various biological activities including fungicidal, bactericidal and herbicidal activities^26^. The indole motif is also a classical pharmacophore present in the COX-2 inhibitors. Indomethacin is a non-selective COX inhibitor, which was used as template structure for the design of several selective COX-2 inhibitors^27,28^. COX-2 has pleiotropic roles in tumorigenesis and cancer proliferation. Its inhibition has chemo- preventive activity against several cancer types. Hence, developing bioactive compounds against this clinically relevant anti-cancer drug target continues to be of interest to the scientific community. This has been recognized that infections and inflammation are associated to cancer, and strong relationships between the presence of inflammation and the development of pre-cancerous abrasions at various anatomic sites have been recognized. Thus, this is one of the considered targets for designing anticancer molecules^29^.

The molecular hybridization (MH) is a powerful approach for rational design of new ligands or prototypes of active molecules^30,31^. The pharmacophoric sub-units in the molecular structure of two or more known bioactive molecules which are linked/fused together, lead to the design of new hybrid structures that maintain pre-selected characteristics of the original templates^32^. This research stemmed from the idea that carbohydrate decoration of compounds having a firmly established pharmacological activity not only can modify pharmacokinetic or the pharmacodynamic properties but also can lead to products with new and unique biological functions^33–39^. The use of carbohydrates as scaffolds or a template for drug discovery has attracted considerable attention in recent years^40,41^. The modern emphasis on chiral synthesis of single-isomer drugs has further enhanced carbohydrate’s utility^42^. However, the drawback associated with use of carbohydrates as drug scaffolds is their metabolism by native enzymes in the body^43,44^. A potentially useful approach to circumvent this drawback could be achieved after synthesis of hybrid molecules with a sugar and a non-sugar domain fused or linked together, possessing druggable properties that may avoid enzymatic breakdown in the body^45–51^. The mounting realization of the potential biological significance of sugar-based hybrid molecules has prompted a number of chemists to attempt their synthesis in recent years.

Therefore, it was envisioned that a suitable combination of carbohydrates with two or more small organic molecules with the diverse structural features may result in the creation of new conjugates (new hybrid molecules). These new molecules may result into better biological activity or prove to be more efficacious, economical, and safe for their use as drug candidates.

A facile efficient synthesis of gluco-, galacto- and manno- (sugar) derived *C*-glycoside linked pyrazoline and isoxazole derivatives has been accomplished by condensation reaction of sugar-linked α, β-unsaturated ketone with hydrazine hydrate and hydroxyl amine hydrochloride under neutral and basic conditions respectively. The new hybrid products were characterized by NMR and mass spectrometry. The purified *C*-glycosides based α, β-unsaturated ketone and their derivatives pyrazoline and isoxazole bridged indole *C*-glycoside hybrids have been screened for their anticancer activity against MCF-7, MDA-MB-453, MDA-MB-231 and MCF-10A. The detailed of these finding are reported in this paper.

## Results and Discussion

### Chemistry

For the efficient synthesis of carbohydrate linked pyrazoline and isoxazole bridged *C*-glycoside of indole hybrids, we identified α, β-unsaturated-*C*-β glyosidic ketone molecule which can be synthesize by using *C*-glycoside **1–3** as substrate. Then resulting α, β-unsaturated-*C*-β glyosidic ketone molecule can be coupled with hydrazine hydrate or hydroxyl amines at room temperature or elevated temperature conditions and get transformed to the designed pyrazoline or isoxazole bridged *C*-glycoside of indole through nucleophilic addition followed by dehydration reactions. When D-glucose was treated with 2,4-pentanedione in the presence of sodium carbonate in water at 90 °C for extended period of time (6–8 h) it undergoes Knoevenagel condensation and furnished selectively β-D-glucosyl-propan-2-ones **1** in very good yield^52^. Similarly, D-galactose and D-mannose were transformed into β-D-galactosyl-propan-2-ones **2** and β-D-mannosyl-propan-2-ones **3** respectively in good isolated yields (Supplementary Information, Scheme S1).

Freshly prepared diverse Indole 3-carboxaldehydes^53^ **5–8** were then coupled with β-D-glycosyl-propan-2-ones **1–3** in aldol condensation fashion using pyrrolidine as catalyst in ethanol at room temperature. The α, β-unsaturated ketone molecules as *C*-glycosides **9–20** were obtained in good to very good isolated yield (70–80%) when reaction mixture stirred for extended period of time (36 h) at room temperature^54^. The results of these reactions and isolated yields are summarized in Table 1. After purification, pure compounds were well characterized through NMR analysis. The E stereochemistry of double bond in these products **9–20** was confirmed through high coupling constant (*J* = 16 Hz) value of olefinic protons.

**Table 1:** Synthesis of *C*-glycosides of diverse indoles **9–20**.


Entry	Sugar	R^1^	R^2^	R^3^	R^4^	Product	Yield (%)
1		H	H	H	H	**9**	80
2		H	NO_2_	H	H	**10**	71
3		H	Br	H	H	**11**	81
4		H	Cl	H	H	**12**	77
5		H	H	H	H	**13**	79
6		H	NO_2_	H	H	**14**	81
7		H	Br	H	H	**15**	84
8		H	Cl	H	H	**16**	80
9		H	H	H	H	**17**	89
10		H	NO_2_	H	H	**18**	85
11		H	Br	H	H	**19**	87
12		H	Cl	H	H	**20**	83

After having gluco- galacto- and manno- linked α, β-unsaturated-*C*-β glycosidic ketone molecules **9–20** prepared and purified, it was planned to couple these with 1,2-dinucleophiles such as hydrazine hydrate and hydroxyl amine hydrochloride in order to get pyrazole/pyrazoline or isoxazole linked *C*-glycosides of indole. Therefore, for optimization purpose first gluco- linked α, β-unsaturated-*C*-β glycosidic ketone molecule **9** was treated with hydrazine hydrate in H_2_O (50 °C), methanol (65 °C), ethanol (rt and 70 °C), in all the reaction conditions pyrazoline was obtained. Reaction completions time was 2 h under heating whereas, it takes 6–7 h under room temperature conditions. Then similar reaction was undertaken in microwave at 70 °C (ethanol solvent) applying 100 W, and we found that, the reaction was completed within 15 min and furnished respective product **21** in 81% isolated yield. So, for the synthesis of pyrazoline compounds, we adopted microwave assisted condition. The structure of compound **21** was established through careful analysis of NMR spectra which shown all characteristic peaks of carbohydrates along with indole nucleus linked with pyrazoline. After, careful NMR and mass spectrometry analysis it was confirmed that the product **21** is having pyrazoline heterocycle formed not pyrazole^55^.

**Scheme 1:**
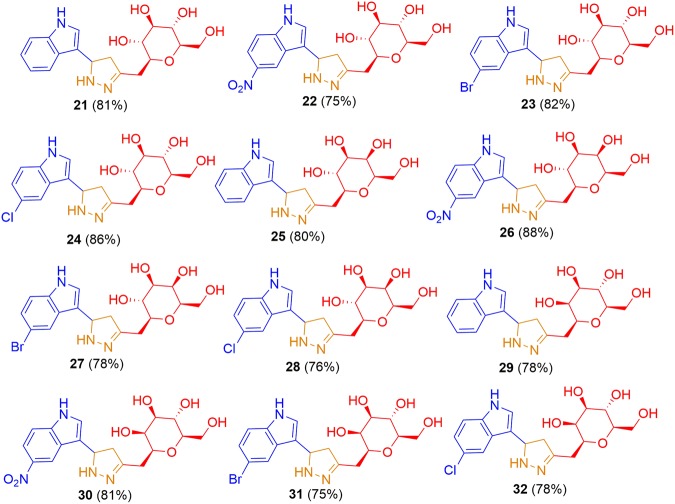
Synthesis of pyrazoline linked indole *C*-glycopyranosides **21–32**.

As shown in Scheme 1 various stereochemically diverse gluco- galacto- and manno- derived α, β-unsaturated-*C*-β glycosidic ketone molecules **9–20** were transformed into respective pyrazoline linked indole *C*-glycopyranosides **21–32** in good to very good yields under microwave reaction conditions.

When hydroxyl amine hydrochloride used as dinucleophile and a test reaction was conducted with gluco- linked α, β-unsaturated-*C*-β glyosidic ketone molecule **9** in ethanol at room temperature it does not give any product (only starting material recovered). Whereas same reaction heated at 60 °C in the presence of potassium carbonate it successfully given isoxazole product **33** in 80% isolated yield.

**Scheme 2:**
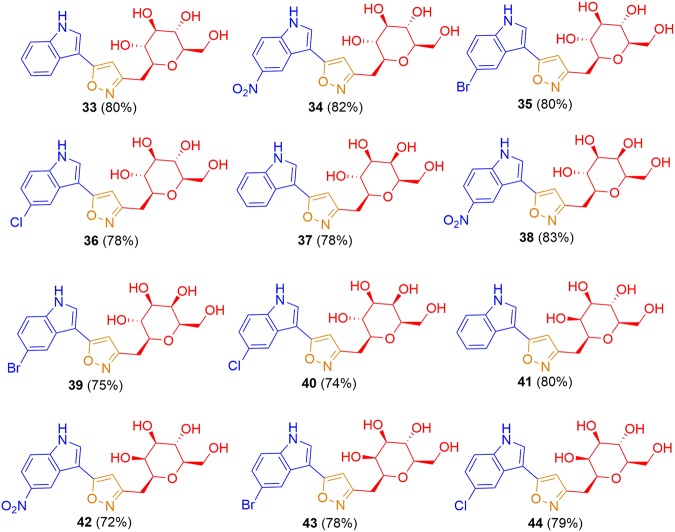
Synthesis of isoxazole linked indole *C*-glycopyranosides **33–44**.

After having optimized reaction condition for isoxazole linked *C*-glycosides, we were interested to investigate the scope of this transformation with different α, β-unsaturated-*C*-β glyosidic ketone molecules having substituted indoles and different sugars under optimized reaction condition. Therefore, various freshly prepared α, β-unsaturated-*C*-β glyosidic ketone molecules **9–20** were subjected for coupling with hydroxyl amine hydrochloride under optimized reaction condition (K_2_CO_3_, 60 °C, 2 h). As shown in Scheme 2, α, β-unsaturated-*C*-β glyosidic ketone molecules **9–20** were successfully coupled with various substituted indoles to afford respective gluco- galacto- and manno- linked isoxazole bridged *C*-glycosides of indole **33–44** in very good yields. The substituents at indole ring neither remarkable affected the yield nor the reaction completion time. All the synthesized products were characterized through detailed NMR, and mass spectrometry. The characteristic isoxazole and pyrazoline proton and carbon peak were observed into final products (Experimental section).

### Biology

Anticancer activity (in terms of growth inhibition/decreased cell viability w.r.t. control) of all synthesized α, β-unsaturated-*C*-glycoside of indoles (**9–20)**, respective pyrazoline bridged *C*-glycosides **21–32** and isoxazole bridged *C*-glycosides **33–44** (n = 36) was investigated by MTT assay in MDA-MB-231 (human breast cancer) cell line at different concentrations for 72 h^56,57^. Anticancer drugs YM155 and menadione were used as positive controls and activity are summarised in Table 2.

**Table 2:** Anticancer activity results.

Compounds	Cell Viability (%) ± SD			Compounds	Cell Viability (%) ± SD		
	50 µM	25 µM	10 µM		50 µM	25 µM	10 µM
**9**	**36.32 ± 4.29**	**54.03 ± 11.19**	71.14 ± 10.53	**27**	97.74 ± 10.12	90.81 ± 7.09	90.05 ± 7.70
**10**	85.37 ± 14.16	84.49 ± 6.74	91.40 ± 10.38	**28**	63.36 ± 17.49	95.16 ± 13.62	99.50 ± 10.20
**11**	107.54 ± 2.89	100.13 ± 8.93	100.25 ± 23.01	**29**	73.55 ± 8.45	76.56 ± 6.58	82.22 ± 7.65
**12**	74.75 ± 7.02	89.80 ± 8.75	79.24 ± 8.51	**30**	87.98 ± 1.10	92.06 ± 8.00	93.84 ± 5.85
**13**	84.22 ± 9.22	94.24 ± 1.96	97.58 ± 2.25	**31**	87.57 ± 6.27	90.13 ± 6.77	91.41 ± 3.62
**14**	**60.56 ± 5.61**	**58.98 ± 12.47**	73.61 ± 11.76	**32**	66.54 ± 7.27	78.49 ± 4.44	74.63 ± 4.70
**15**	67.46 ± 17.69	113.07 ± 23.87	125.13 ± 3.51	**33**	92.63 ± 15.25	106.38 ± 7.40	103.51 ± 12.72
**16**	56.70 ± 17.82	89.59 ± 12.91	75.04 ± 14.31	**34**	**43.40 ± 0.26**	**46.10 ± 3.32**	68.37 ± 5.36
**17**	79.43 ± 10.36	80.48 ± 2.58	84.10 ± 4.09	**35**	93.89 ± 9.08	95.31 ± 3.60	95.45 ± 4.91
**18**	69.81 ± 20.40	78.47 ± 6.73	71.68 ± 28.36	**36**	99.33 ± 4.28	96.80 ± 11.58	97.84 ± 11.61
**19**	71.48 ± 6.24	89.20 ± 28.63	102.51 ± 10.39	**37**	64.41 ± 7.77	76.39 ± 10.41	74.75 ± 10.73
**20**	**61.98 ± 4.91**	**54.28 ± 16.32**	56.70 ± 23.23	**38**	94.90 ± 4.49	94.45 ± 3.67	99.10 ± 5.63
**21**	75.04 ± 2.62	76.76 ± 2.43	73.46 ± 2.43	**39**	90.31 ± 2.88	89.42 ± 5.32	120.42 ± 17.62
**22**	70.33 ± 6.17	68.62 ± 11.05	70.40 ± 12.97	**40**	87.93 ± 4.71	96.03 ± 1.81	93.70 ± 2.68
**23**	81.53 ± 5.45	88.82 ± 2.22	104.77 ± 11.28	**41**	73.96 ± 17.57	81.22 ± 5.17	89.23 ± 3.70
**24**	77.74 ± 2.56	77.51 ± 8.96	82.23 ± 3.50	**42**	95.23 ± 13.64	93.37 ± 15.68	92.62 ± 11.51
**25**	**43.82 ± 13.04**	**54.17 ± 7.06**	97.58 ± 3.36	**43**	95.70 ± 3.26	85.24 ± 2.93	96.59 ± 3.98
**26**	99.50 ± 1.88	98.25 ± 0.34	100.58 ± 9.67	**44**	81.68 ± 3.45	82.94 ± 5.28	83.90 ± 4.08
YM155 (20 nM)	27.39 ± 4.93			Menadione (20 µM)	20.77 ± 8.70		

**Figure 2:**
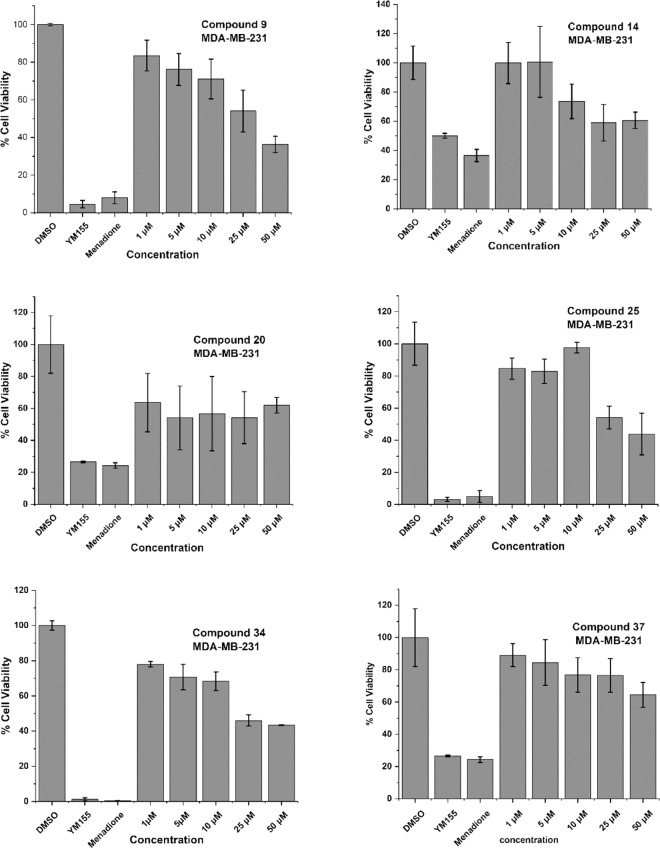
Anti-cancer activities at different dilutions for active compounds. *P values were found at 1 µM, 5 µM, 10 µM, 25 µM, 50 µM for compound **9** (0.0357, 0.0123, 0.0133, 0.0031, 0.0001), **14** (0.0863, 0.0660, 0.0246, 0.0041, 0.0012), **20** (0.0338, 0.0091, 0.0341, 0.0130, 0.0193), **25** (0.0872, 0.0600, 0.0999, 0.0095, 0.0097), **34** (0.0004, 0.0041, 0.0021, 0.0001, 0.0001) and **37** (0.0165, 0.0534, 0.0796, 0.0666, 0.0150) respectively compare to DMSO control.

The results summarized in Table 2 showed moderate growth inhibition at 10 μM concentration in all compounds except for **11**, **26**, **27**, **33**, **35**, **36**, **38**, **39**, **42** and **43**. Better inhibition of cell viability was observed with compounds **9**, **14**, **20**, **25** and **34** in MDA-MB-231 (breast cancer cells). These compounds show almost 50% inhibition at 25 µM concentration. These five compounds were further tested in serial dilution to find out IC_50_ and results are shown in Fig. 2.

**Figure 3:**
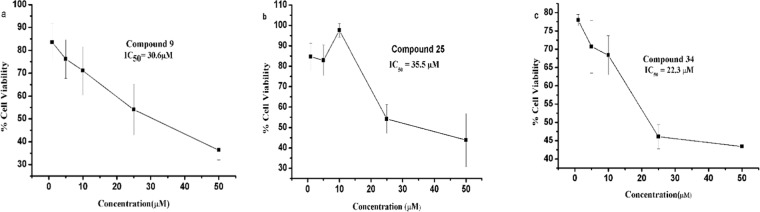
IC_50_ of the most active compounds. To calculate half maximal inhibitory concentration (IC_50_) of compounds **9, 25** and **34**, MDA-MB-231 cells were treated for 48 h with different concentrations (1, 5, 10, 25 and 50 µM) of the respective drugs and tested for cell viability by the MTT assay. IC_50_ values were determined by plotting values of percent cell viability against concentration of each of these compounds. (**a–c**) IC_50_ values for compounds **9, 25** and **34** against MDA-MB-231 cells were 30.6, 35.5, and 22.3 µM respectively. The experiments were performed in triplicates, n = 3 and ± SE value was calculated for each data point.

The IC_50_ values for most active compounds **9**, **25** and **34** were calculated and are presented in Fig. 3. The IC_50_ values for compound **9**, **25** and **34** against MDA-MB-231 breast cancer cells line were found 30.6, 35.5 and 22.3 µM, respectively.

MDA-MB-231 represents a specific subtype, known as the triple negative breast cancer (TNBC). We extended our investigation with these five hit compounds (**9**, **14**, **20**, **25**, **34** and **37**) in two other different cancer cell lines (MCF-7 and MDA-MB-453), each representing a separate class of breast cancers (MCF-7: hormone receptor/HR-positive; MDA-MB-453: human epidermal growth factor 2 receptor/HER2 positive). We also included normal mammary epithelial MCF-10A cells in our experiments to confirm if the growth-inhibitory activities of the selected compounds are truly cancer cell-specific. The activity data from three different cell lines (tested at 1, 10 and 25 μM) are summarised in Table 3.

**Table 3:** Anticancer activity screening results.

Compds	Cell Viability (%) ± SD								
	MCF-7			MDA-MB-453			MCF-10A		
	25 µM	10 µM	1 µM	25 µM	10 µM	1 µM	25 µM	50 µM	100 µM
**9**	**53.7 ± 4.0**	62.3 ± 3.6	79.7 ± 1.18	96.1 ± 35.7	76.4 ± 5.4	80.6 ± 8.0	87.6 ± 1.5	81.3 ± 2.9	84.2 ± 3.0
**14**	66.8 ± 3.0	69.8 ± 1.8	100.0 ± 4.5	65.3 ± 5.8	82.2 ± 11.5	80.8 ± 3.2	86.8 ± 2.8	84.6 ± 0.7	80.0 ± 1.5
**20**	**47.3 ± 7.6**	58.7 ± 2.4	79.2 ± 2.6	66.4 ± 3.2	74.8 ± 1.5	105 ± 8.1	85.8 ± 5.3	78.8 ± 3.0	36.5 ± 3.4
**25**	**19.9 ± 2.9**	34.0 ± 2.5	66.0 ± 4.7	69.6 ± 5.3	78.3 ± 4.7	88.8 ± 5.2	87.6 ± 2.2	84.6 ± 1.7	82.4 ± 2.6
**34**	**23.1 ± 4.4**	28.4 ± 0.9	41.3 ± 3.1	66.8 ± 1.7	80.9 ± 1.2	92.5 ± 4.6	87.6 ± 1.5	80.1 ± 2.8	84.2 ± 3.0
**37**	**19.1 ± 3.5**	21.2 ± 3.8	30.8 ± 4.2	96.1 ± 35.7	76.4 ± 5.4	95.4 ± 4.8	86.8 ± 2.8	83.2 ± 5.8	81.0 ± 1.5
YM155 (20 nM)	37.2 ± 1.7	37.2 ± 1.7	37.2 ± 1.7	33.2 ± 1.5	33.2 ± 1.5	33.2 ± 1.5	48.2 ± 1.6	48.2 ± 1.6	48.2 ± 1.6

The IC_50_ values for most active compounds **9**, **20**, **25**, **34** and **37** were calculated using five different concentration (1.0, 5.0, 10.0, 25 and 50 µM) and are presented in Table 4. The IC_50_ values for compound **9, 20,** **25**, **34** and **37** against MCF-7 breast cancer cells line were found 30.0, 20.99, 4.67, 0.71 and 0.67 µM, respectively. These compounds did not reach up to IC_50_ value against MDA-MB-453 cells. Furthermore, none of these compounds did have any growth inhibitory activity against normal breast epithelial cell (MCF-10A). The IC_50_ values for these compounds against MDA-MB-231 cells (Fig. 2) are also included in Table 4 for comparison.

**Table 4:** IC_50_ value of most active compounds. **25, 34** and **37**. (Nd = not determind).

Cancer Cells	Compounds and IC_50_ (µM)				
	9	20	25	34	37
MCF-7	30.0	20.99	**4.67**	**0.71**	**0.67**
MDA-MB-231	30.6	Nd	35.5	22.3	Nd

**Figure 4:**
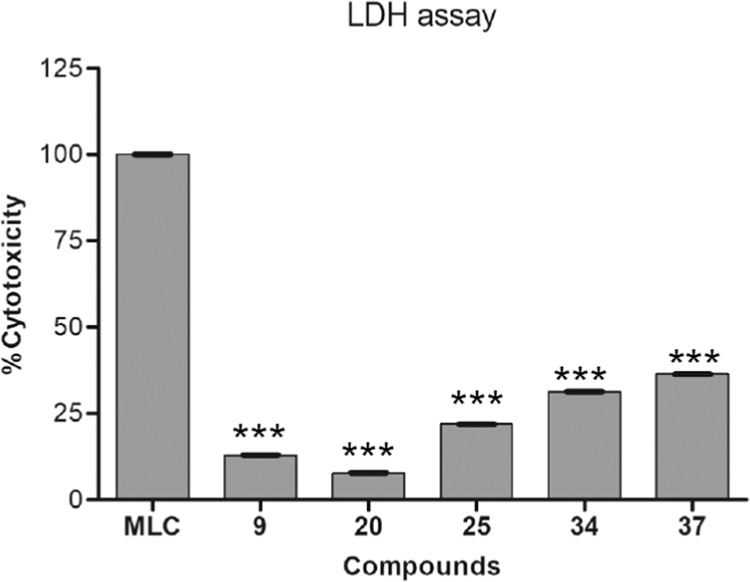
LDH Assay of the most active compounds (25 µM). *P values for compound **9**, **20**, **25**, **34** and **37** were found <0.0001 compare to MLC.

When living cells treated with a cytotoxic compound, they could either stop growing and dividing, or die through either of two distinct processes i.e. necrosis or apoptosis^58^. Basically, cells undergoing necrosis (accidental cell death) swell and lose membrane integrity before shutting down and releasing their intracellular contents into the surrounding environment. This type of cell death is usually triggered by external factors such as toxic chemical or traumatic physical events. When the cell membranes are damaged or compromised in any way, lactate dehydrogenase (LDH), a soluble yet stable enzyme found inside every living cell, gets released into the surrounding extracellular space. The presence of this enzyme in the culture medium can be used as a cell death marker. The relative amounts of live and dead cells within the medium can then be quantitated by measuring the amount of released LDH using a colorimetric or fluorometric LDH cytotoxicity assay. Herein we confirmed cytotoxicity of active compounds **9**, **20**, **25**, **34** and **37** by fluorometric LDH cytotoxicity assay using maximum LDH control (MLC) which shows % of cytotoxicity as 13, 8, 22, 31 and 36 respectively at 25 µM concentration (Fig. 4).

In order to understand the cytotoxicity mechanism of the active compounds, COX-2 assay was performed. The *in-vitro* COX-2 inhibitory activity of the active compounds (**9**, **20**, **25**, **34** and **37)** was evaluated using celecoxib as a reference with a fluorescence-based COX-2 assay (“COX-2 Fluorescent Inhibitor Screening Assay Kit”, Item no. K547, Bio Vision, USA). In this method we detect the fluorescence compound PGG2 which can easily be analysed with an excitation wavelength of 540 nm and emission wavelength of 590 nm. The result of COX-2 inhibitory activity has been summarised in Table 5 at two different concentrations. It’s very clear from results shown in Table 5 that active compounds showing some inhibition and maximum COX-2 inhibition shown by compound **37** which is in consistence with its cytotoxic activity against MCF-7 cells (Table 4).

**Table 5:** COX-2 assay results (Fluorescence-based COX-2 assay and % inhibition).

Concentration	Compounds and % inhibition				
	9	20	25	34	37
25 μM	25	13.7	6.7	4.9	61
50 μM	71	39	27	59	95

**Figure 5:**
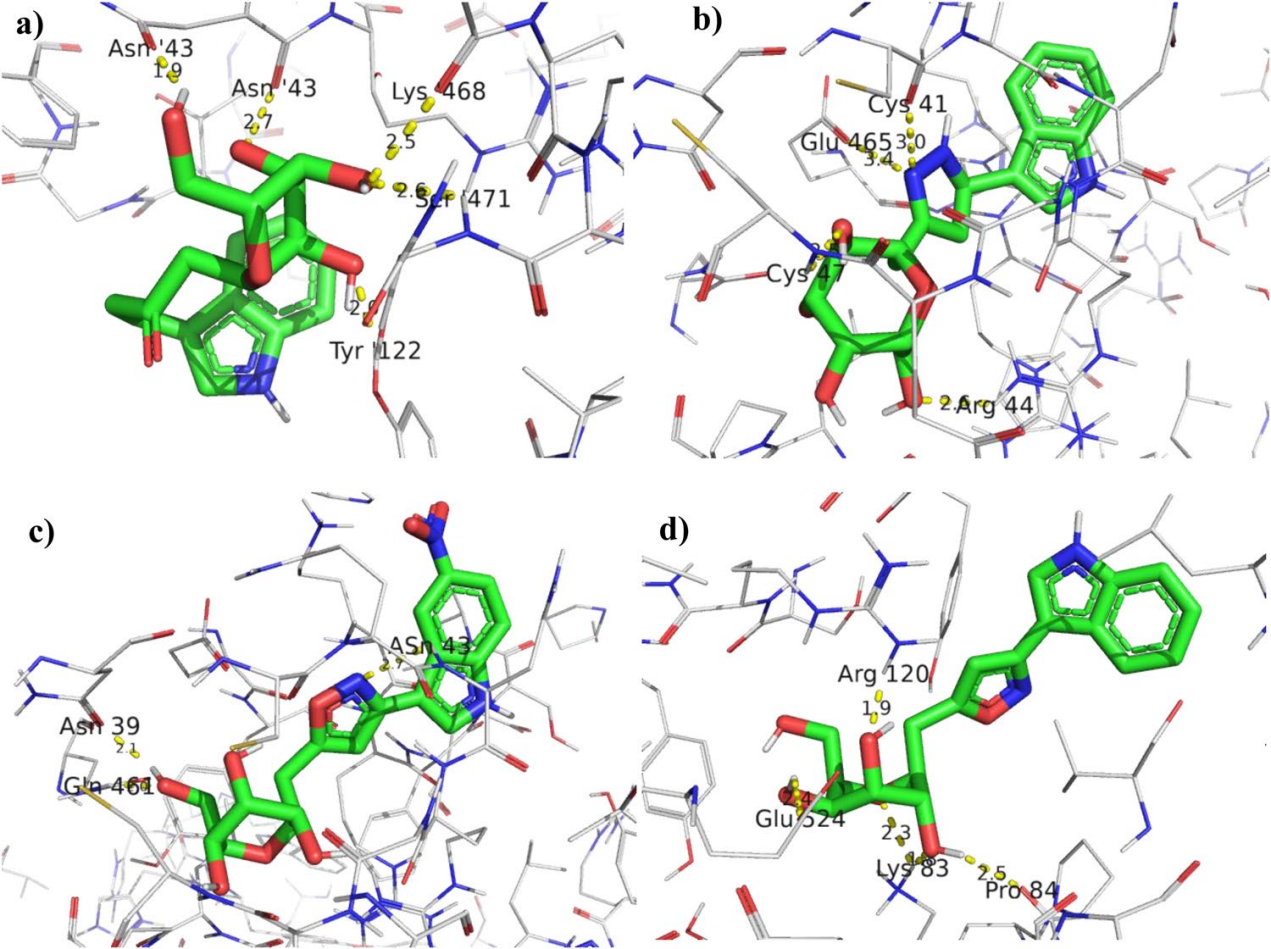
Three-dimensional (3D) orientation of the docked compounds **9**, **25**, **34** and **37** (**a**–**d** respectively) with COX-2 enzyme (H bonds interactions are shown as yellow dotted lines).

To undertand the COX-2 inhibition and molecular interaction of the active compounds with the COX-2 enzyme (PDB code: 1CX2), molecular docking studies were performed using Autodock-Vina software^59^ (for conversion to supported docking format) and PyMol software (for visualization and analysis of the interaction of ligands with the protein after docking). The three-dimensional orientation of the docked compounds showing important binding interaction through hydrogen bonds with carbohydrate moiety and hydrophobic interactions as shown Fig. 5.

## Conclusions

In summary, we report an efficient synthesis of pyrazoline and isoxazole bridged *C*-glycoside of indoles. The stereochemical diversity in these products was inherited from natural carbohydrates. Gluco- galacto- and manno- linked with diversely substituted indoles, α, β-unsaturated ketones were successfully prepared in good yield under moderate reaction conditions. The method involves robust synthesis of pyrazoline and isoxazole bridged *C*-glycoside of indoles, starting from freshly prepared α, β-unsaturated ketone and dinucleophiles (hydrazine hydrate and hydroxyl amine hydrochlorides) in very good yields. The method is endowed with several unique merits including benign reaction conditions, stereochemical diversity, broad substrate scope etc. The method has successfully applied for synthesis of a diverse library of thirty-six carbohydrate-linked hybrid molecules. Subsequently, we demonstrated that five of these gluco- galacto- and manno- linked *C*-glycosides possess anticancer activity at micromolar levels (22.3–35.5 µM) in MDA-MB-231. It showing very less cytotoxicity for MDA-MB-453.Two of these hit compounds were found active against MCF-7 at nanomolar range (670–467 nM) and they were less toxic to healthy cell line (MCF-10A). So, these compounds are more potent for MCF-7. It has also observed that active molecules inhibiting COX-2 enzymes, which may be the reason of cytotoxicity. The docking study reveals that carbohydrate moiety is important and involved in hydrogen bonded interaction with COX-2 enzyme. The Cytotoxicity has been confirmed by LDH assay.

## Experimental

### General experimental methods

All experiments were performed in an oven-dried apparatus. High resolution mass spectra obtained from a quadrupole/TOF mass spectrometer with an ESI source. Solvents were distilled by standard distillation procedure and stored in 4Å and 3Å molecular sieves. ^1^H (400 MHz), ^13^C (100 MHz) NMR spectra was recorded with a Bruker AMX-400 MHz instrument. ^1^H and ^13^C chemical shifts are referenced to the solvents residual signals D_2_O ^1^H NMR δ 4.79, DMSO ^1^H 2.50 and δ 39.52 for ^13^C and CD_3_OD,^1^H NMR δ 4.87, 3.31 and ^13^C δ 49.00 reported in parts per million (ppm) at 25 °C. Coupling constants are expressed in hertz (Hz). Reactions were monitored by thin-layer chromatography carried out on 0.25 mm E. Merck silica gel plates (60F-254), Spots were visualized by phosphomolybdic acid and 10% H_2_SO_4_ in ethanol. *C*-glycosides, α, β-unsaturated ketones were freshly prepared in laboratory. Organocatalysts used in this study were purchased from Sigma-Aldrich and Alfa-aesar. The IC_50_ values were computed by using origin software. The grid box dimension used for the docking studies was the standard size x = y = z = 40Å.

### General experimental procedure for synthesis of indole linked α-β-unsaturated ketones (Chalcones) **9–20**

A mixture of α, β-unsaturated ketone **1** (300 mg, 1.36 mmol), indole-3-carboxaldehyde **5** (197 mg, 1.36 mmol) and pyrrolidine **(**22 μL, 19.34 mg, 0.272 mmol) was stirred at rt for 24–36 h. The progress of reaction was monitored by TLC (3:7 = Methanol: Ethyl acetate, v/v). After completion, reaction mixture was concentrated on rotary evaporator to make slurry. The crude product was purified by flash column chromatography which furnished the pure products **9** in 80% (378 mg) isolated yield (Table 1). The similar reaction protocol was adopted for the synthesis of other similar compounds (**10–20)**.

#### (E)-4-(1H-indol-3-yl)-1-((2S,3R,4R,5S,6R)-3,4,5-trihydroxy-6-(hydroxymethyl) tetrahydro-2H-pyran-2-yl)but-3-en-2-one (**9**)

Yellow amorphous solid, R_f_ = 0.6, (3:7, methanol: ethyl acetate, v/v), ^1^H NMR (400 MHz, CD_3_OD): δ 8.01 (d, *J* = 16 Hz, 1H), 7.95 (dd, *J* = 2.4 Hz, *J* = 6 Hz, 1H), 7.78 (s, 1H), 7.48 (dd, *J* = 2 Hz, 6.8 Hz, 1H), 7.30–7.21 (m, 2H), 6.91 (d, *J* = 16 Hz, 1H), 3.83 (d, *J* = 2.4 Hz, 1H), 3.81–3.78 (m, 1H), 3.69–3.62 (m, 1H), 3.43–3.39 (m, 1H), 3.29–3.25 (m, 2H), 3.22 (d, *J* = 9.2 Hz, 1H), 3.17 (dd, *J* = 2.4 Hz, *J* = 15.6 Hz, 1H), 2.91 (dd, *J* = 8.8 Hz, *J* = 15.6 Hz, 1H); ^13^C NMR (100 MHz, CD_3_OD): δ 200.3, 139.3, 137.9, 125.2, 123.6, 122.6, 122.2, 120.9, 120.4, 119.8, 112.7, 111.9, 111.7, 80.2, 78.3, 76.5, 75.8, 73.8, 70.3, 62.9, 61.4, 42.5. HRMS (ESI), *m/z* calcd for C_18_H_21_NO_6_ [M + H]^+^ 348.1442; Found: 348.1468.

#### (E)-4-(5-nitro-1H-indol-3-yl)-1-((2S,3R,4R,5S,6R)-3,4,5-trihydroxy-6-(hydroxymethyl) tetrahydro-2H-pyran-2-yl)but-3-en-2-one (**10**)

Yellow amorphous solid, R_f_ = 0.52 (3:7, methanol: ethyl acetate, v/v), ^1^H NMR (400 MHz, CD_3_OD): δ 8.79 (d, *J* = 2.0 Hz, 1H), 8.13 (dd, *J* = 2.0 Hz, *J* = 9.2 Hz, 1H), 7.97 (d, *J* = 14.8 Hz, 1H), 7.93 (s, 1H), 7.58 (d, *J* = 8.8 Hz, 1H), 6.90 (d, *J* = 16.4 Hz, 1H), 3.84–3.82 (m, 1H), 3.81–3.79 (m, 1H), 3.71–3.68 (m, 1H), 3.67–3.64 (m, 1H), 3.62 (t, *J* = 2.4 Hz, 1H), 3.12–3.07 (m, 1H), 2.98–2.94 (m, 1H), 2.91 (dd, *J* = 4.8 Hz, *J* = 15.6 Hz, 1H), 2.62 (dd, *J* = 9.2 Hz, *J* = 16.0 Hz, 1H); ^13^C NMR (100 MHz, CD_3_OD): δ 200.0, 142.5, 140.7, 136.7, 133.7, 124.7, 122.6, 117.8, 116.2, 114.3, 112.1, 80.2, 78.3, 78.2, 76.3, 75.8, 73.8, 73.7, 70.3, 61.4, 45.7. HRMS (ESI), *m/z* calcd for C_18_H_20_N_2_O_8_ [M + H]^+^ 393.1304; Found: 393.1292.

#### (E)-4-(5-bromo-1H-indol-3-yl)-1-((2S,3R,4R,5S,6R)-3,4,5-trihydroxy-6-(hydroxymethyl)tetrahydro-2H-pyran-2-yl)but-3-en-2-one (**11**)

Yellow amorphous solid, R_f_ = 0.71 (3:7, methanol: ethyl acetate, v/v), ^1^H NMR (400 MHz, CD_3_OD): δ 7.99 (d, *J* = 1.2 Hz, 1H), 7.88 (d, *J* = 16.4 Hz, 1H), 7.75 (s, 1H), 7.35–7.30 (m, 1H), 7.30 (dd, *J* = 1.6 Hz, 8.8 Hz, 1H), 6.77 (d, *J* = 16.4 Hz, 1H), 3.77 (d, *J* = 2.4 Hz, 1H), 3.75–3.72 (m, 1H), 3.62 (dd, *J* = 4.80 Hz, 12.0Hz, 1H), 3.38–3.30 (m, 2H), 3.33–3.30 (m, 1H), 3.23–3.19 (m, 1H), 3.10 (dd, *J* = 2.8 Hz, *J* = 15.6 Hz, 1H), 2.86 (dd, *J* = 9.2 Hz, *J* = 15.2 Hz, 1H); ^13^C NMR (100 MHz, CD_3_OD): δ 200.0, 138.0, 136.5, 132.4, 126.9, 125.4, 122.2, 121.0, 114.1, 113.4, 112.2, 80.2, 78.3, 76.4, 73.8, 70.2, 61.3, 42.6 HRMS (ESI), *m/z* calcd for C_18_H_20_BrNO_6_ [M + H]^+^ 426.0547; Found: 426.1925.

#### (E)-4-(5-chloro-1H-indol-3-yl)-1-((2S,3R,4R,5S,6R)-3,4,5-trihydroxy-6-(hydroxymethyl) tetrahydro-2H-pyran-2-yl)but-3-en-2-one (**12**)

Light yellow solid, R_f_ = 0.81 (3:7, methanol: ethyl acetate, v/v), ^1^H NMR (400 MHz, CD_3_OD): δ 8.03 (d, *J* = 1.2 Hz, 1H), 7.92 (d, *J* = 16.0 Hz, 1H), 7.53 (t, *J* = 8.0 Hz, 1H), 7.37 (d, *J* = 8.8 Hz, 1H), 7.33 (dd, *J* = 1.6 Hz, *J* = 8.8 Hz, 1H), 6.80 (d, *J* = 16.0 Hz, 1H), 3.80 (d, *J* = 2.0 Hz, 1H), 3.77 (d, *J* = 2.4 Hz, 1H), 3.42–3.38 (m, 1H), 3.36–3.33 (m, 1H), 3.66–3.65 (m, 1H), 3.63 (dd, *J* = 2.4 Hz, *J* = 8.0 Hz, 1H), 3.19 (d, *J* = 9.2 Hz, 1H), 3.13 (dd, *J* = 2.4 Hz, *J* = 15.6 Hz, 1H), 2.88 (dd, *J* = 9.2 Hz, *J* = 15.2 Hz, 1H); ^13^C NMR (100 MHz, CD_3_OD): δ 200.1, 138.1, 132.4, 129.6, 128.1, 126.9, 125.4, 122.2, 121.0, 114.1, 113.4, 80.1, 78.3, 76.4, 73.8, 70.2, 61.3, 42.5. HRMS (ESI), *m/z* calcd for C_18_H_20_ClNO_6_O_3_ [M + H]^+^ 382.1052; Found: 382.1052.

#### (E)-4-(1H-indol-3-yl)-1-((2S,3R,4R,5R,6R)-3,4,5-trihydroxy-6-(hydroxymethyl) tetrahydro-2H-pyran-2-yl)but-3-en-2-one (**13**)

Reddish brown solid^,^ R_f_ = 0.66 (3:7, methanol: ethyl acetate, v/v) ^1^H NMR (400 MHz, CD_3_OD): δ 7.98 (d, *J* = 16.0 Hz, 1H), 7.91 (d, *J* = 7.2 Hz, 1H), 7.74 (s, 1 H), 7.46 (d, *J* = 7.6 Hz, 1H), 7.25–7.19 (m, 2H), 6.88 (d, *J* = 16 Hz, 1H), 3.94–3.90 (m, 1H), 3.78–3.74 (m, 1H), 3.70–3.67 (m, 2H), 3.56–3.51 (m, 1H), 3.15 (m, 1H), 3.00–2.95 (m, 1H), 2.89 (dd, *J* = 17.2 Hz, *J* = 14.8 Hz, 1H), 2.66 (dd, *J* = 9.2 Hz, *J* = 15.6 Hz, 1H); ^13^C NMR (100 MHz, CD_3_OD): δ 200.4, 139.1, 137.9, 131.8, 125.2, 122.6, 120.9, 120.5, 119.8, 112.7,111.8, 78.7, 77.0,74.9, 71.0, 69.5, 61.2, 42.4. HRMS (ESI), *m/z* calcd for C_18_H_21_NO_6_ [M + Na]^+^ 370.1261; Found: 370.1282.

#### (E)-4-(5-nitro-1H-indol-3-yl)-1-((2S,3R,4R,5R,6R)-3,4,5-trihydroxy-6-(hydroxymethyl) tetrahydro-2H-pyran-2-yl)but-3-en-2-one (**14**)

Yellow colour sticky solid, R_f_ = 0.75 (3:7, methanol: ethyl acetate, v/v), ^1^H NMR (400 MHz, CD_3_OD): δ 8.78 (d, *J* = 2.0 Hz, 1H), 8.14 (dd, *J* = 2 Hz, *J* = 9.2 Hz, 1H), 7.95 (d, *J* = 16.4 Hz, 2H), 7.58 (d, *J* = 8.8 Hz, 1H), 6.89 (d, *J* = 16 Hz, 1H), 3.95 (d, *J* = 2.4 Hz, 1H), 3.72–3.70 (m, 2H), 3.69–3.67 (m, 1H), 3.62 (s, 1H), 3.57–3.54 (m, 2H), 3.18 (dd, *J* = 2.4 Hz, *J* = 16 Hz, 1H), 3.03 (dd, *J* = 9.2 Hz, *J* = 15.6 Hz, 1H); ^13^C NMR (100 MHz, CD_3_OD): δ 200.1, 142.5, 140.8, 136.4, 133.5, 122.7, 117.7, 116.1, 114.4, 112.1, 111.4, 78.7, 76.9, 74.9, 70.9, 69.5, 61.2, 42.5. HRMS (ESI), *m/z* calcd for C_18_H_20_N_2_O_8_ [M + H]^+^ 393.1304; Found: 393.1292.

#### (E)-4-(5-bromo-1H-indol-3-yl)-1-((2S,3R,4R,5R,6R)-3,4,5-trihydroxy-6-(hydroxy methyl) tetrahydro-2H-pyran-2-yl)but-3-en-2-one (**15**)

Light brown solid, R_f_ = 0.73 (3:7, methanol: ethyl acetate, v/v), ^1^H NMR (400 MHz, CD_3_OD): δ 8.04 (d, *J* = 1.2 Hz, 1H), 7.93 (d, *J* = 16 Hz, 1H), 7.80 (s, 1H), 7.40 (d, *J* = 8.4 Hz, 1H), 7.35 (dd, *J* = 2 Hz, *J* = 8.4 Hz, 1H), 6.82 (d, *J* = 16.0 Hz, 1H), 3.95 (d, *J* = 2 Hz, 1H), 3.77 (td, *J* = 2 Hz, 8.4 Hz, 17.2 Hz,1H), 3.72 (d, *J* = 2.4 Hz, 1H), 3.70 (d, *J* = 1.2 Hz, 1H), 3.56–3.54 (m, 2H), 3.34 (dt, *J* = 1.6 Hz, 3.2 Hz, 1H), 3.15 (dd, *J* = 2.8 Hz, *J* = 15.2 Hz, 1H), 3.00 (dd, *J* = 3.6 Hz, *J* = 15.2 Hz, 1H); ^13^C NMR (100 MHz, CD_3_OD): δ 200.2, 137.9, 136.5, 132.2, 127.0, 125.3, 122.2, 121.2, 114.1, 113.4, 112.2, 78.7, 77.1, 74.9, 71.0, 69.5, 61.2, 42.6. HRMS (ESI), *m/z* calcd for C_18_H_20_BrNO_6_ [M + H]^+^ 426.0547; Found: 426.1925.

#### (E)-4-(5-chloro-1H-indol-3-yl)-1-((2S,3R,4R,5R,6R)-3,4,5-trihydroxy-6-(hydroxyl methyl) tetrahydro-2H-pyran-2-yl)but-3-en-2-one (**16**)

Light brown solid, R_f_ = 0.81 (3:7, methanol: ethyl acetate), ^1^H NMR (400 MHz, CD_3_OD): δ 7.93 (t, *J* = 16 Hz, 1H), 7.89 (d, *J* = 1.6 Hz, 1H), 7.80 (s, 1H), 7.43 (d, *J* = 8.8 Hz, 1H), 7.21 (dd, *J* = 2.0 Hz, *J* = 8.8 Hz, 1H), 6.82 (d, *J* = 16 Hz, 1H), 3.92 (d, *J* = 2.0 Hz, 1H), 3.74 (td, *J* = 2.0 Hz, *J* = 8.8 Hz, 1H), 3.68–3.65 (m, 2H), 3.55–3.52 (m, 1H), 3.50–3.46 (m, 2H), 3.12 (dd, *J* = 2.4 Hz, *J* = 15.6 Hz, 1H), 2.97 (dd, *J* = 9.2 Hz, *J* = 15.6 Hz, 1H) ^13^C NMR (100 MHz, CD_3_OD): δ 200.3, 138.0, 132.5, 126.6, 122.7, 121.1, 119.1, 113.0, 112.3, 78.7, 77.0, 74.9, 71.0, 69.4, 61.1, 42.6. HRMS (ESI), *m/z* calcd for C_18_H_20_ClNO_6_ [M + Na]^+^ 404.0871; Found: 404.0863.

#### (E)-4-(1H-indol-3-yl)-1-((2S,3S,4R,5S,6R)-3,4,5-trihydroxy-6-(hydroxymethyl) tetrahydro-2H-pyran-2-yl)but-3-en-2-one (**17**)

Reddish brown sticky solid, R_f_ = 0.71 (3:7, methanol: ethyl acetate, v/v), ^1^H NMR (400 MHz, CD_3_OD): δ 7.98 (d, *J* = 16.0 Hz, 1H), 7.91 (dd, *J* = 2.0 Hz, *J* = 6.4 Hz, 1H), 7.74 (s, 1H), 7.46 (dd, *J* = 1.2 Hz, *J* = 7.2 Hz, 1H), 7.26–7.19 (m, 2H), 6.84 (d, *J* = 16.0 Hz, 1H), 4.05 (t, *J* = 6.4 Hz, 1H), 3.86–3.83 (m, 2H), 3.73 (dd, *J* = 5.6 Hz, *J* = 11.6 Hz, 1H), 3.65 (t, *J* = 9.2 Hz, 1H), 3.56 (dd, *J* = 3.6 Hz, 1H), 3.29–3.24 (m, 1H), 3.16 (dd, *J* = 7.2 Hz, *J* = 16.4 Hz, 1H), 2.98 (dd, *J* = 6.0 Hz, 16.4 Hz, 1H); ^13^C NMR (100 MHz, CD_3_OD): δ 199.6, 139.1, 137.9, 131.8, 125.2, 122.6, 120.9, 120.5, 119.8, 112.6, 111.8, 80.6, 75.1, 74.9, 71.3, 67.2, 61.6, 41.0. HRMS (ESI) *m/z* calcd for C_18_H_21_NO_6_ [M + H]^+^ 348.1442; Found: 348.1468.

#### (E)-4-(5-nitro-1H-indol-3-yl)-1-((2S,3R,4R,5S,6R)-3,4,5-trihydroxy-6-(hydroxyl methyl) tetrahydro-2H-pyran-2-yl) but-3-en-2-one (**18**)

Yellow brown solid, R_f_ = 0.72 (3:7, methanol: ethyl acetate, v/v), ^1^H NMR (400 MHz, CD_3_OD): δ 8.77 (d, *J* = 2 Hz, 1H), 8.13 (dd, *J* = 2 Hz, 8.8 Hz, 1H), 7.95 (d, *J* = 17.6 Hz, 2H), 7.57 (d, *J* = 8.8 Hz, 1H), 6.85 (d, *J* = 16.4 Hz, 1H), 4.09 (t, *J* = 6.4 Hz, 1H), 3.87–3.86 (m, 1H), 3.83 (dd, *J* = 2.4 Hz, *J* = 10.0 Hz, 1H), 3.76–3.70 (m, 1H), 3.67–3.62 (m, 1H), 3.58–3.54 (m, 1H), 3.30–3.26 (m, 1H), 3.22 (dd, *J* = 5.2 Hz, 16.4 Hz, 1H), 3.00 (dd, *J* = 5.6 Hz, *J* = 16.4 Hz, 1H); ^13^C NMR (100 MHz, CD_3_OD): δ 199.2, 142.5, 140.7, 136.6, 133.5, 124.7, 122.6, 117.7, 116.1, 114.3,112.1, 80.6, 75.1, 74.8, 71.3, 67.2, 61.6, 41.1. HRMS (ESI), *m/z* calcd for C_18_H_20_N_2_O_8_ [M + H]^+^ 393.1304, Found: 393.1292.

#### (E)-4-(1H-indol-3-yl)-1-((2S,3S,4R,5S,6R)-3,4,5-trihydroxy-6-(hydroxymethyl) tetrahydro-2H-pyran-2-yl) but-3-en-2-one (**19**)

Reddish brown solid, R_f_ = 0.77 (3:7, methanol: ethyl acetate, v/v), ^1^H NMR (400 MHz, CD_3_OD): δ 8.02 (d, *J* = 1.6 Hz, 1H), 7.92 (d, *J* = 16.4 Hz, 1H), 7.78 (s, 1H), 7.38 (dd, *J* = 1.6 Hz, *J* = 8.8 Hz, 1H), 7.33 (dd, *J* = 1.6 Hz, *J* = 8.8 Hz 1H), 6.77 (d, *J* = 16.0 Hz, 1H), 3.85–3.83 (m, 1H), 3.82–3.79 (m, 1H), 3.74–3.73 (m, 1H), 3.71–3.69 (m, 1H), 3.67–3.64 (m, 1H), 3.60 (d, *J* = 2.0 Hz, 1H), 3.53–3.50 (m, 1H), 2,96 (dd, *J* = 5.6 Hz, *J* = 16.4 Hz, 1H), 2.72 (dd, *J* = 5.2 Hz, *J* = 17.2 Hz, 1H); ^13^C NMR (100 MHz, CD_3_OD): δ 199.5, 138.1, 132.4, 127.0, 125.4, 122.2, 121.1, 114.1, 113.5, 112.1, 80.6, 75.0, 74.9, 74.1, 71.3, 71.0, 67.2, 61.5, 41.0 HRMS (ESI), *m/z* calcd for C_18_H_20_BrNO_6_ [M + H]^+^ 426.0547; Found: 426.1925.

#### (E)-4-(5-chloro-1H-indol-3-yl)-1-((2S,3S,4R,5S,6R)-3,4,5-trihydroxy-6-(hydroxy methyl) tetrahydro-2H-pyran-2-yl)but-3-en-2-one (**20**)

Reddish brown solid, R_f_ = 0.52(3:7, methanol: ethyl acetate, v/v), ^1^H NMR (400 MHz, CD_3_OD): δ 7.72 (d, *J* = 20.8 Hz, 1H), 7.45 (s, 1H), 7.34 (d, *J* = 8.0 Hz, 1H), 7.18–7.06 (m, 1H), 6.80 (d, *J* = 16.4 Hz, 1H), 6.32 (dd, *J* = 2.8 Hz, *J* = 7.2 Hz, 1H), 3.95–3.87 (m, 1H), 3.82–3.78 (m, 1H), 3.74–3.71 (m, 1H), 3.67–3.61 (m, 1H), 3.46–3.37 (m, 2H), 3.33–3.32 (m, 1H), 3.14–3.12 (m, 1H), 2.76–2.65 (m, 1H); ^13^C NMR (100 MHz, CD_3_OD): δ 199.6, 154.3, 153.3, 135.8, 125.8, 121.7, 119.5, 118.5, 113.8, 112.8, 108.5, 106.6, 73.3, 71.5, 67.0, 63.3, 62.9, 45.5; HRMS (ESI), *m/z* calcd for C_18_H_20_ClNO_6_ [M + H]^+^ 382.1052; Found: 382.1052.

### General experimental procedure for the synthesis of Pyrazoline derivatives** 21–32**

In a microwave vial, was taken α, β-unsaturated ketone (100 mg, 0.288 mmol) in 2 mL of ethanol then added hydrazine hydrate (144 mg, 2.88 mmol, 141 μL) to it. Reaction mixture was heated in a microwave vial at 70 °C (100 W) for 15 minutes under microwave condition, the progress of reaction was monitored by TLC (3:7, methanol: ethyl acetate, v/v). After completion, reaction mixture was concentrated on rotary evaporator to get crude residue. The crude residue was purified by flash column chromatography to obtain the pure compound **21** (81%) as isolated yield. Similar reaction protocol was followed for preparation of rest of the pyrazoline compounds **22–32**.

#### (2S,3R,4R,5S,6R)-2-((5-(1H-indol-3-yl)-4,5-dihydro-1H-pyrazol-3-yl)methyl)-6-(hydroxy-methyl)tetrahydro-2H-pyran-3,4,5-triol (**21**)

Brown solid, R_f_ = 0.52 (3:7, methanol: ethyl acetate, v/v), ^1^H NMR (400 MHz, D_2_O): δ 7.53–7.48 (m, 2H), 7.26–7.20 (m, 2H), 7.15–7.12 (m, 1H), 3.71 (s, 1H), 3.58–3.57 (m, 1H), 3.55–3.52 (m, 1H), 3.50–3.48 (m, 1H), 3.36–3.34 (m, 1H), 3.31 (brs, 1H), 2.29–3.22 (m, 2H), 2.97–2.92 (m, 1H), 2.88–2.86 (m, 1H), 2.64–2.55 (m, 1H); ^13^C NMR (100 MHz, D_2_O): δ 173.1, 159.9, 136.6, 122.5, 122.1, 119.3, 118.7, 115.1, 112.0, 79.5, 77.3, 73.5, 69.7, 62.6, 60.7, 41.9, 41.6, 32.2. HRMS (ESI), *m/z* calcd for C_18_H_23_N_3_O_5_ [M + H]^+^ 362.1716; Found: 362.1733.

#### (2R,3S,4R,5R,6S)-2-(hydroxymethyl)-6-((5-(5-nitro-1H-indol-3-yl)-4,5-dihydro-1H-pyrazol-3-yl)methyl)tetrahydro-2H-pyran-3,4,5-triol (**22**)

Yellow solid, R_f_ = 0.47 (3:7, methanol: ethyl acetate, v/v), ^1^H NMR (400 MHz, CD_3_OD) δ 8.56 (d, *J* = 11.2 Hz, 1H), 8.15 (dd, *J* = 3.2 Hz, *J* = 6.0 Hz, 1H), 7.99 (d, *J* = 9.2 Hz, 1H), 7.43 (dd, *J* = 6.8 Hz, *J* = 8.0 Hz, 1 H), 3.78–3.65 (m, 1 H), 3.59–3.54 (m, 1 H), 3.48–3.44 (m, 1 H), 3.34–3.31 (m, 1 H), 3.22–3.18 (m, 2 H), 3.15–3.07 (m, 1 H), 2.94 (dd, *J* = 8.0 Hz, *J* = 16.4 Hz, 1 H), 2.55 (dd, *J* = 9.2 Hz, 1 H), 2.05 (s, 1 H), 1.22 (brs, 2 H); ^13^C NMR (100 MHz, CD_3_OD): δ 170.8, 156.9, 140.9, 132.2, 125.3, 118.5, 116.7, 116.0, 111.4, 121.1, 80.3, 78.2, 77.8, 77.3, 74.0, 73.8, 70.2, 63.0, 61.4, 42.6, 32.5. HRMS (ESI), *m/z* calcd for C_18_H_22_N_4_O_7_ [M + H]^+^ 407.1561; Found: 407.1594.

#### (2 S,3 R,4 R,5 S,6 R)-2-((5-(5-bromo-1H-indol-3-yl)-4,5-dihydro-1H-pyrazol-3-yl)methyl)-6-(hydroxymethyl)tetrahydro-2H-pyran-3,4,5-triol (**23**)

Yellowish brown solid, R_f_ = 0.48 (3:7, methanol: ethyl acetate, v/v) ^1^H NMR (400 MHz, CD_3_OD): δ 7.70 (dd, *J* = 2.0 Hz, *J* = 11.6 Hz, 1 H), 7.25 (d, *J* = 4.0 Hz, 1 H), 7.22 (s, 1 H), 7.18 (dd, *J* = 2.0 Hz, *J* = 8.4 Hz, 1 H), 3.81 (dd, *J* = 2.4 Hz, *J* = 12.0 Hz, 1 H), 3.73 (dd, *J* = 2.4 Hz, 12.0 Hz, 1 H), 3.64–3.58 (m, 1 H), 3.47–3.43 (m, 1 H), 3.37–3.34 (m, 2 H), 3.32–3.29 (m, 1 H), 3.27–3.25 (m, 1 H), 3.24–3.20 (m, 1 H), 2.97–2.87 (m, 2 H), 2.55 (dd, *J* = 9.6 Hz, *J* = 14.4 Hz, 1 H); ^13^C NMR (CD_3_OD, 100 MHz): δ 157.1, 135.8, 135.7, 127.6, 127.3, 124.0, 123.2, 121.0, 120.9, 112.8, 111.7, 80.2, 78.2, 77.7, 77.2, 74.0, 73.8, 70.3, 61.6, 61.4, 42.4, 42.0, 32.5. HRMS (ESI), *m/z* calcd for C_18_H_22_BrN_3_O_5_ [M + H]^+^ 440.0816; Found: 440.0806.

#### (2 S,3 R,4 R,5 S,6 R)-2-((5-(5-chloro-1H-indol-3-yl)-4,5-dihydro-1H-pyrazol-3-yl)methyl)-6-(hydroxymethyl)tetrahydro-2H-pyran-3,4,5-triol (**24**)

Brown yellow solid, R_f_ = 0.71 (3:7, methanol: ethyl acetate, v/v), ^1^H NMR (400 MHz, CD_3_OD) δ 7.71 (dd, *J* = 1.6 Hz, *J* = 12.8 Hz, 1 H), 7.26 (t, *J* = 8.8 Hz, 2 H), 7.19 (dd, *J* = 1.6 Hz, *J* = 8.8 Hz, 1 H), 3.86–3.79 (m, 1 H), 3.73–3.69 (m, 1 H), 3.66–3.57 (m, 2 H), 3.47–3.41 (m, 1 H), 3.20–3.16 (m, 1 H), 3.14–3.09 (m, 1 H), 2.94 (dd, *J* = 6.8 Hz, 1 H), 2.61–2.52 (m, 1 H), 1.93–1.85 (m, 2 H), 1.24–1.21 (m, 1 H); ^13^C NMR δ (100 MHz, CD_3_OD): δ 172.9, 136.6, 123.4, 122.2, 119.6, 118.5, 112.0, 78.5, 77.3, 73.8, 70.8, 69.0, 61.0, 48.8, 46.6, 32.4. HRMS (ESI), *m/z* calcd for C_18_H_22_ClN_3_O_5_ [M + H^+^ 396.1321; Found: 396.1339.

#### (2 S,3 R,4 R,5 R,6 R)-2-((5-(1H-indol-3-yl)-4,5-dihydro-1H-pyrazol-3-yl)methyl)-6-(hydroxy-methyl)tetrahydro-2H-pyran-3,4,5-triol (**25**)

Brown amorphous solid, R_f_=0.48 (3:7, methanol: ethyl acetate, v/v), ^1^H NMR (400 MHz, D_2_O): δ 7.59 (t, *J* = 7.2 Hz, 1 H), 7.52 (d, *J* = 8.4 Hz, 1 H), 7.26 (s, 1 H), 7.25 (d, *J* = 6.8 Hz, 1 H), 7.16 (t, *J* = 7.6 Hz , 1 H), 3.96–3.94 (m, 1 H), 3.66 (brs, 1 H), 3.65–3.62 (m, 1 H), 3.59–3.52 (m, 1 H), 3.5 5–3.53 (m, 1 H), 3.52–3.50 (m, 1 H), 3.21–3.12 (m, 1 H), 2.98 (dd, *J* = 3.6 Hz, *J* = 13.2 Hz, 1 H), 2.62 (dd, *J* = 5.2 Hz, *J* = 14.4 Hz, 1 H), 2.11–2.10 (m, 1 H)), 1.28 (t, *J* = 7.2 Hz, 2 H); ^13^C NMR (100 MHz, CD_3_OD): δ 162.4, 158.7, 140.9,122.7, 122.1, 119.4, 118.7, 112.0, 78.3, 73.8, 69.0, 61.0, 54.9, 41.6, 32.5; HRMS (ESI), m/z calcd for C_18_H_23_N_3_O_5_ [M + H]^+^ 362.1716; Found: 362.1739.

#### (2 S,3 R,4 R,5 R,6 R)-2-((5-(5-nitro-1H-indol-3-yl)-4,5-dihydro-1H-pyrazol-3-yl)methyl)-6-(hydroxymethyl)tetrahydro-2H-pyran-3,4,5-triol (**26**)

Yellow amorphous solid, R_f_ = 0.91 (3 :7, methanol: ethyl acetate, v/v), ^1^H NMR (400 MHz, CD_3_OD): δ 8.49 (dd, *J* = 2.0 Hz, *J* = 7.6 Hz, 1 H), 7.93 (dd, *J* = 2.0 Hz, *J* = 9.2 Hz, 1 H) 7.42–7.38 (m, 1 H), 7.37 (s, 1 H), 3.84 (brs, 1 H), 3.62–3.60 (m, 1 H), 3.58–3.57 (m, 1 H), 3.55–3.54 (m, 1 H), 3.41–3.40 (m, 1 H), 3.39–3.36 (m, 1 H), 3.20–3.12 (m, 1 H), 2.89 (dd, *J* = 4.8 Hz, *J* = 17.7 Hz, 1 H), 2.58 (dd, *J* = 10.8 Hz, *J* = 12.4 Hz, 1 H), 2.89 (s, 1 H), 1.12 (t, J = 7.2 Hz, 2 H); ^13^C NMR (100 MHz, CD_3_OD): δ157.3, 153.3, 153.0, 140.9, 140.2, 140.0, 125.7, 125.1, 124.9, 118.8, 118.6, 116.7, 116.6, 115.9, 115.8, 111.3, 78.9, 78.3, 78.0, 74.9, 74.8, 71.1, 69.5, 61.42.5, 32.5 HRMS (ESI), *m/z* calcd for C_18_H_22_N_4_O_7_ [M + H^+^ 407.1561; Found: 407.1594.

#### (2 S,3 R,4 R,5 R,6 R)-2-((5-(5-bromo-1H-indol-3-yl)-4,5-dihydro-1H-pyrazol-3-yl) methyl)-6-(hydroxymethyl)tetrahydro-2H-pyran-3,4,5-triol (**27**)

Yellowish brown solid, R_f_ = 0.68 (3:7, methanol: ethyl acetate, v/v), ^1^H NMR (400 MHz, CD_3_OD): δ 7.70 (d, *J* = 8.4 Hz, 1 H), 7.27 (s, 1 H), 7.24 (d, *J* = 10.0 Hz, 1 H), 7.17 (d, *J* = 10.4 Hz, 1 H), 3.87 (d, *J* = 2.8 Hz, 1 H), 3.70–3.64 (m, 1 H), 3.62–3.57 (m, 1 H), 3.50–3.48 (m, 1 H), 3.40–3.36 (m, 1 H), 3.29–3.28 (m, 1 H), 3.21–3.10 (m, 1 H), 2.90 (dd, *J* = 8.4 Hz, *J* = 16.4 Hz, 1 H), 2.60 (dd, *J* = 9.2 Hz, *J* = 15.6 Hz, 1 H), 2.17–1.83 (m, 2 H), 1.16 (t, *J* = 7.2 Hz, 1 H); ^13^C NMR (100 MHz, CD_3_OD): δ 157.6, 135.7, 135.6, 127.6, 127.3, 123.9, 123.3, 121.0, 120.9, 115.7, 115.4, 112.8, 111.7, 78.8, 78.7, 77.9, 74.8, 71.0, 69.5, 61.4, 42.2, 41.9, 32.5. HRMS (ESI), *m/z* calcd for C_18_H_22_BrN_3_O_5_ [M + H]^+^ 440.0816; Found: 440.0806.

#### (2 S,3 R,4 R,5 R,6 R)-2-((5-(5-chloro-1H-indol-3-yl)-4,5-dihydro-1H-pyrazol-3-yl) methyl)-6-(hydroxymethyl)tetrahydro-2H-pyran-3,4,5-triol (**28**)

Light brown solid, R_f_ = 0.59 (3:7, methanol: ethyl acetate, v/v), ^1^H NMR (400 MHz, CD_3_OD): δ 7.52 (dd, *J* = 2.4 Hz, *J* = 8.8 Hz, 1 H), 7.29 (d, *J* = 10.0 Hz, 1 H), 7.22 (s, 1 H), 7.04–7.01 (m, 1 H), 3.87 (d, *J* = 2.8 Hz, 1 H), 3.60–3.61 (m, 2 H), 3.60–3.55 (m, 1 H), 3.45 (brs, 1 H), 3.40–3.36 (m, 1 H), 3 .19–3.08 (m, 1 H), 2.91 (dd, *J* = 6.8 Hz, *J* = 18.0 Hz, 1 H), 2.59 (dd, *J* = 9.2 Hz, *J* = 14.4 Hz, 1 H), 1.86 (d, *J* = 37.2 Hz, 2 H), 1.14 (t, *J* = 6.8 Hz, 1 H); ^13^C NMR (100 MHz, CD_3_OD): δ 157.5, 126.9, 126.7, 124.2, 123.5, 121.4, 117.9, 117.8, 115.7, 115.5, 112.4, 112.3, 78.8, 78.2, 77.9, 74.8, 71.1, 69.5, 61.3, 42.2, 41.8, 32.5. HRMS (ESI), *m/z* calcd for C_18_H_22_ClN_3_O_5_ [M + H^+^ 396.1321; Found: 396.1339.

#### (2 S,3 S,4 R,5 S,6 R)-2-((5-(1H-indol-3-yl)-4,5-dihydro-1H-pyrazol-3-yl)methyl)-6-(hydroxy-methyl)tetrahydro-2H-pyran-3,4,5-triol (**29**)

Light yellow solid, R_f_ = 0.40 (3:7, methanol: ethyl acetate, v/v), ^1^H NMR (400 MHz, DMSO-*d6*): δ 9.02 (brs, 1 H, NH), 7.53 (d, *J* = 8.0 Hz, 1 H), 7.34 (d, *J* = 8.0 Hz, 1 H), 7.22 (t, *J* = 2.4 Hz, 1 H), 7.06 (t, *J* = 7.6 Hz, 1 H), 6.95 (t, *J* = 7.6 Hz, 1 H), 3.63 (d, *J* = 8.4 Hz, 1 H), 3.59 (d, *J* = 6.8 Hz 1 H), 3.51–3.47 (m, 1 H), 3.45–3.41 (m, 1 H), 3.37–3.35 (m, 1 H), 3.34–3.31 (m, 2 H), 2.82 (dd, *J* = 7.2 Hz, *J* = 18.4 Hz, 1 H), 2.68 (dd, *J* = 4.8 Hz, *J* = 16.4 Hz, 1 H), 1.82 (brs, 2 H), 1.22 (brs, 1 H); ^13^C NMR (100 MHz, DMSO-*d6*): δ 168.9, 152.2, 137.1, 126.2, 137.1, 126.2, 122.6, 118.7, 111.9, 81.6, 76.5, 75.4, 70.7, 67.7, 61.9, 49.0, 33.6. HRMS (ESI), *m/z* calcd for C_18_H_23_N_3_O_5_ [M + H]^+^ 362.1716; Found: 362.1739.

#### (2 S,3 S,4 R,5 S,6 R)-2-((5-(5-nitro-1H-indol-3-yl)-4,5-dihydro-1H-pyrazol-3-yl)methyl)-6-(hydroxymethyl)tetrahydro-2H-pyran-3,4,5-triol (**30**)

Yellow amorphous solid, R_f_ = 0.76 (3:7, methanol: ethyl acetate, v/v), ^1^H NMR (400 MHz, D_2_O): δ 7.70 (dd, *J* = 2.0 Hz, *J* = 11.6 Hz, 1 H), 7.25 (dd, *J* = 8.8 Hz, *J* = 21.6,  2H), 7.18 (dd, *J* = 2.0 Hz, *J* = 8.8 Hz, 1 H), 3.81 (dd, *J* = 2.0 Hz, *J* = 14.0 Hz, 1 H), 3.64–3.59 (m, 1 H), 3.50–3.43 (m, 1 H), 3.42–3.37 (m, 1 H), 3.36–3.34 (m, 1 H), 3.27–3.20 (m, 2 H), 2.97–2.93 (m, 1 H), 2.90 (dd, *J* = 2.8 Hz, *J* = 14.4 Hz, 1 H), 2.56 (dd, *J* = 6.4 Hz, *J* = 10.4 Hz, 1 H);. ^13^C NMR (100 MHz, D_2_O): δ 157.1, 135.8, 135.7, 127.6, 127.3, 124.0, 123.2, 121.0, 120.9, 112.8, 111.7, 80.2, 78.2, 78.6, 77.7, 77.2, 74.0, 73.8, 70.3, 61.6, 61.4, 42.4, 42.0, 32.5 HRMS (ESI), *m/z* calcd for C_18_H_22_N_4_O_37_ [M + Na]^+^ 429.1381; Found: 429.2400.

#### (2 S,3 S,4 R,5 S,6 R)-2-((5-(5-bromo-1H-indol-3-yl)-4,5-dihydro-1H-pyrazol-3-yl)methyl)-6-(hydroxymethyl)tetrahydro-2H-pyran-3,4,5-triol (**31**)

Brown amorphous solid, R_f_ = 0.42 (3:7, methanol: ethyl acetate, v/v), ^1^H NMR (400 MHz, CD_3_OD): δ 7.64 (d, *J* = 9.2 Hz, 1 H), 7.22 (d, *J* = 8.4 Hz, 1 H), 7.18 (d, *J* = 5.2 Hz, 1 H), 7.12 (d, *J* = 8.8 Hz, 1 H), 3.73 (d, *J* = 10.0 Hz,  2H), 3.64 (brs, 1 H), 3.63 (brs, 1 H), 3.54–3.52 (m, 1 H), 3.45–3.42 (m, 1 H), 3.13–3.08 (m, 1 H), 2.77 (dd, *J* = 9.6 Hz, *J* = 13.6 Hz, 1 H), 2.53 (dd, *J* = 16.0 Hz, *J* = 8.0 Hz, 1 H), 2.02–1.98 (m, 1 H), 1.77 (s, 2 H); ^13^C NMR (100 MHz, CD_3_OD): δ 171.0, 156.8, 135.7, 131.0, 128.4, 123.9, 123.3. 121.0, 115.6, 112.8, 111.7, 80.6, 76.3, 76.1, 75.0, 71.2, 67.3, 30.2 HRMS (ESI), *m/z* calcd for C_18_H_22_BrN_3_O_5_ [M + H]^+^ 440.0816; Found: 440.0806.

#### (2 S,3 S,4 R,5 S,6 R)-2-((5-(5-chloro-1H-indol-3-yl)-4,5-dihydro-1H-pyrazol-3-yl) methyl)-6-(hydroxymethyl)tetrahydro-2H-pyran-3,4,5-triol (**32**)

Light brown Solid, R_f_ = 0.42 (3:7, methanol: ethyl acetate, v/v), ^1^H NMR (400 MHz, CD_3_OD): δ 7.64 (d, *J* = 7.2 Hz, 1 H), 7.30 (d, *J* = 8.4 Hz, 1 H), 7.21 (s, 1 H), 7.06 (d, *J* = 8.0 Hz, 1 H), 3.94–3.89 (m, 1 H), 3.79–3.74 (m, 1 H), 3.69–3.66 (m, 1 H), 3.64–3.61 (m, 1 H), 3.58 (brs, 1 H), 3.50–3.47 (m, 2 H), 3.15–3.13 (m, 1 H), 2.74–2.73 (m, 1 H), 1.88 (brs, 2 H), 1.27 (brs, 1 H); ^13^C NMR (100 MHz, CD_3_OD): δ 164.6, 154.8, 135.6, 125.4, 124.3, 121.4, 118.1, 116.0, 112.2, 81.0, 76.1, 75.0, 70.5, 67.2, 62.9, 45.1, 30.4 HRMS (ESI) *m/z* calcd for C_18_H_22_ClN_3_O_5_ [M + H]^+^ 396.1321; Found: 396.1339.

### General experimental procedure for the synthesis of isoxazole derivatives **33–44**

In a 25 mL round bottom flask, was taken 100 mg (0.288 mmol) α, β-unsaturated ketone **9** in 20 mL of ethanol, then hydroxylamine hydrochloride (200 mg, 2.88 mmol) and potassium carbonate (398 mg, 2.88 mmol) were added to it and heated at 78–80 °C. The progress of reaction was monitored by thin layer chromatography (3:7, methanol: ethyl acetate, v/v). After completion, reaction mixture was concentrated on rotary evaporator to get crude residue. The crude residue was purified by flash column chromatography to get pure compound **33** in 80% isolated yield. Similar reaction protocol was adopted for the preparation of rest of the isoxazole derivatives **34–44** using respective substrates.

#### (2 S,3 R,4 R,5 S,6 R)-2-((5-(1H-indol-3-yl)isoxazol-3-yl)methyl)-6-(hydroxymethyl) tetrahydro-2H-pyran-3,4,5-triol (**33**)

Brown colour solid, R_f_ = 0.63 (3:7, methanol: ethyl acetate, v/v), ^1^H NMR (400 MHz, CD_3_OD): δ 7.92 (dd, *J* = 2.8 Hz, *J* = 6.0 Hz, 1 H), 7.80 (s, 1 H), 7.46 (dd, *J* = 1.6 Hz, *J* = 6.8 Hz, 1 H), 7.22 (dd, *J* = 1.6 Hz, *J* = 6.8 Hz, 1 H), 7.19 (dd, *J* = 1.2 Hz, *J* = 8.0 Hz, 1 H), 6.66 (s, 1 H), 3.87–3.84 (m, 1 H), 3.77 (t, *J* = 4.8 Hz, 1 H), 3.68–3.64 (m, 1 H), 3.54–3.46 (m, 1 H), 3.43–3.33 (m, 2 H), 3.25 (d, *J* = 2.0 Hz, 1 H), 3.19 (t, *J* = 8.8 Hz, 1 H), 2.85 (dd, *J* = 8.8 Hz, *J* = 14.8 Hz, 1 H); ^13^C NMR (400 MHz, CD_3_OD): δ 167.2, 156.4, 137.2, 124.9, 123.2, 122.2, 121.4, 120.5, 119.3, 118.8, 118.6, 111.6, 111.3, 97.7, 80.1, 80.0, 78.1, 77.1, 75.7, 73.9, 70.3, 62.8, 61.4, 42.6, 37.6. HRMS (ESI), *m/z* calcd for C_20_H_20_N_2_O_6_ [M + H]^+^ 361.1394; Found: 361.1401.

#### (2 R,3 S,4 R,5 R,6 S)-2-(hydroxymethyl)-6-((5-(5-nitro-1H-indol-3-yl)isoxazol-3-yl)methyl) tetrahydro-2H-pyran-3,4,5-triol (**34**)

Yellow colour amorphous solid, R_f_ = 0.57 (3:7, methanol: ethyl acetate, v/v), ^1^H NMR (400 MHz, CD_3_OD): δ 8.38 (d, *J* = 18.8 Hz, 1 H), 8.02 (s, 1 H), 7.58 (dd, s, 1 H), 7.50 (d, *J* = 11.2 Hz, 1 H), 6.34 (s, 1 H), 3.87 (s, 1 H), 3.83 (s, 1 H), 3.70–3.67 (m, 1 H), 3.50–3.48 (m,  2H), 3.46–3.45 (m, 1 H), 3.23 (dd, *J* = 4.0 Hz, *J* = 14.4 Hz, 1 H), 2.75 (dd, *J* = 2.0 Hz, *J* = 6.0 Hz, 1 H); ^13^C NMR (100 MHz, CD_3_OD): δ 165.7, 157.5,147.7, 142.5, 139.6, 128.5, 117.6, 117.3, 116.8, 116.4, 98.9, 80.2, 78.2, 78.0, 76.1, 74.4, 73.9, 73.4, 70.4,. 70.1, 61.1,45.8, 45.2, 37.3 HRMS (ESI), *m/z* calcd for C_18_H_19_N_3_O_8_ [M + H]^+^ 406.1245; Found: 406.1265.

#### (2 S,3 R,4 R,5 S,6 R)-2-((5-(5-bromo-1H-indol-3-yl)isoxazol-3-yl)methyl)-6-(hydroxy-methyl)tetrahydro-2H-pyran-3,4,5-triol (**35**)

Brown colour solid, R_f_ = 0.66 (3:7, methanol: ethyl acetate, v/v), ^1^H NMR (400 MHz, CD_3_OD): δ 8.09 (d, *J* = 1.6 Hz, 1 H), 7.86 (s, 1 H), 7.41 (d, *J* = 8.8 Hz,1 H), 7.34 (dd, *J* = 1.6 Hz, *J* = 8.8 Hz, 1 H), 6.68 (s, 1 H), 3.88 (d, *J* = 12.0 Hz, 1 H), 3.70–3.66 (m, 2 H), 3.55–3.50 (m, 1 H), 3.27 (d, *J* = 2.4 Hz, 1 H), 3.23–3.17 (m, 2 H), 2.88 (dd, *J* = 5.2 Hz, *J* = 15.2 Hz, 1 H), 2.77 (dd, *J* = 6.0 Hz, *J* = 17.2 Hz, 1 H);.^13^C NMR (100 MHz, CD_3_OD): δ 166.4, 161.9, 135.4, 125.2, 125.1, 121.9, 113.7, 113.2, 103.9, 98.0, 80.6, 80.3, 78.3, 78.0, 74.9, 73.4, 70.4, 70.2, 61.6, 42.8, 40.6. HRMS (ESI), *m/z* calcd for C_18_H_19_BrN_2_O_6_ [M + H]^+^ 441.0481; Found: 441.0626.

#### (2 S,3 R,4 R,5 S,6 R)-2-((5-(5-chloro-1H-indol-3-yl)isoxazol-3-yl)methyl)-6-(hydroxy methyl) tetrahydro-2H-pyran-3,4,5-triol (**36**)

Brown colour solid, R_f_ = 0.71 (3:7, methanol: ethyl acetate, v/v), ^1^H NMR (400 MHz, CD_3_OD): δ 7.52 (dd, *J* = 1.6 Hz, *J* = 8.0 Hz, 1 H), 7.38 (s, 1 H), 7.36 (d, *J* = 7.6 Hz, 1 H), 7.10 (d, *J* = 8.8 Hz, 1 H), 5.79 (t, *J* = 10.0 Hz, 1 H), 3.93 (d, *J* = 14.0 Hz, 1 H), 3.74 (d, *J* = 5.6 Hz, 1 H), 3.71–3.68 (m, 1 H), 3.54–3.50 (m, 2 H), 3.33–3.31 (m, 1 H), 3.06–3.00 (m, 1 H), 2.70 (dd, *J* = 8.8 Hz, *J* = 16.4 Hz, 1 H), 2.36 (dd, *J* = 9.2 Hz, *J* = 14.4 Hz, 1 H); ^13^C NMR (100 MHz, CD_3_OD): δ 158.7, 157.1, 156.8, 135.6, 135.5, 126.6, 126.5, 124.7, 124.6, 121.7, 118.0, 114.0, 113.7, 112.6, 78.7, 78.6, 78.0, 77.8, 77.6, 76.3, 76.2, 74.8, 74.7, 69.4, 61.3, 61.2, 42.9, 42.6, 37.6 HRMS (ESI), *m/z* calcd for C_18_H_19_ClN_2_O_6_ [M + H]^+^ 395.1004; Found: 395.1044.

#### (2 S,3 R,4 R,5 R,6 R)-2-((5-(1H-indol-3-yl)isoxazol-3-yl)methyl)-6-(hydroxymethyl) tetrahydro-2H-pyran-3,4,5-triol (**37**)

Brown colour solid, R_f_ = 0.84 (3:7, methanol: ethyl acetate, v/v), ^1^H NMR (400 MHz, CD_3_OD): δ 7.60 (t, *J* = 8.0 Hz, 1 H), 7.43 (d, *J* = 8.4 Hz, 1 H), 7.35 (d, *J* = 10.8 Hz, 1 H), 7.18 (t, *J* = 8.0 Hz, 1 H), 7.10 (ddd, *J* = 0.8 Hz, *J* = 2.8 Hz, 1 H), 5.87 (t, *J* = 10.0 Hz, 1 H), 3.98–3.96 (m, 1 H), 3.78–3.72 (m, 2 H), 3.58–3.52 (m, 1 H), 3.50–3.45 (m, 2 H), 3.43–3.39 (m, 1 H), 3.07 (dd, *J* = 11.2 Hz, *J* = 16.0 Hz, 1 H), 2.74 (dd, *J* = 8.8 Hz, *J* = 15.2 Hz, 1 H); ^13^C NMR (100 MHz, CD_3_OD): δ 158.6, 158.5, 137.3, 125.4, 123.2, 123.1, 121.5, 118.9, 118.7, 118.6, 114.0, 113.8, 111.3, 111.2, 78.8, 78.7, 78.0, 77.6, 76.8, 76.7, 74.8, 70.9, 69.4, 61.3, 42.9, 42.6, 37.6 HRMS (ESI) *m/z* calcd for C_18_H_20_N_2_O_6_ [M + H]^+^ 361.1394; Found: 361.1392.

#### (2 R,3 R,4 R,5 R,6 S)-2-(hydroxymethyl)-6-((5-(5-nitro-1H-indol-3-yl)isoxazol-3-yl) methyl) tetrahydro-2H-pyran-3,4,5-triol (**38**)

Yellow amorphous solid, R_f_ = 0.62 (3:7, methanol: ethyl acetate, v/v), ^1^H NMR (400 MHz, CD_3_OD): δ 8.59 (s, 1 H), 8.59–8.55 (m, 1 H), 8.19–8.00 (m, 1 H), 7.65–7.53 (m, 1 H), 6.81(s, 1 H), 3.89 (brs, 1 H), 3.70–3.67 (m, 1 H), 3.62 (brs, 2 H), 3.45 (brs, 2 H), 3.37 (brs, 1 H), 2.76 (d, *J* = 14.0 Hz, 1 H), 2.35 (dd, *J* = 8.8 Hz, *J* = 14.0 Hz, 1 H); ^13^C NMR (100 MHz, CD_3_OD): δ 170.4, 165.5, 162.3, 156.9, 156.6, 142.1, 141.6, 117.9, 117.5, 117.4, 117.2, 116.4, 116.2, 112.1, 111.7, 100.3, 98.9, 78.6, 77.8, 77.3, 74.8, 74.6, 71.6, 71.1, 69.5, 62.9, 61.3, 37.6. HRMS (ESI) *m/z* calcd for C_18_H_19_N_3_O_8_[M + H]^+^ 406.1245; Found: 406.1239.

#### (2 S,3 R,4 R,5 R,6 R)-2-((5-(5-bromo-1H-indol-3-yl)isoxazol-3-yl)methyl)-6-(hydroxy methyl) tetrahydro-2H-pyran-3,4,5-triol (**39**)

Brown sticky solid, R_f_ = 0.62 (3:7, methanol: ethyl acetate, v/v), ^1^H NMR (400 MHz, CD_3_OD): δ 7.69 (dd, *J* = 0.8 Hz, 8.0 Hz, 1 H), 7.41 (d, *J* = 9.2 Hz, 1 H), 7.36–7.35 (m, 1 H), 7.24 (d, *J* = 8.4 Hz, 1 H), 5.82 (t, *J* = 10.0 Hz, 1 H), 3.97 (s, 1 H), 3.76 (d, *J* = 6.4 Hz, 1 H), 3.73–3.70 (m, 1 H), 3.59–3.55 (m, 2 H), 3.50–3.45 (m, 1 H), 3.09–3.02 (m, 1 H), 2.72 (dd, *J* = 8.8 Hz, *J* = 15.2 Hz, 1 H), 2.38 (dd, *J* = 8.8 Hz, *J* = 14.4 Hz, 1 H); ^13^C NMR (100 MHz, CD_3_OD): δ 168.9, 158.8, 135.9, 135.8, 127.3, 127.1, 124.5, 124.2, 121.1, 113.9, 113.7, 113.0, 112.0, 78.6, 78.0, 77.8, 77.6, 76.3, 76.2, 74.8, 74.7, 71.0, 69.5, 69.4, 61.4, 42.9, 42.6, 37.6. HRMS (ESI), *m/z* calcd for C_18_H_19_BrN_2_O_6_ [M + H]^+^ 441.0481; Found: 441.0606.

#### (2 S,3 R,4 R,5 R,6 R)-2-((5-(5-chloro-1H-indol-3-yl)isoxazol-3-yl )methyl)-6-(hydroxy methyl) tetrahydro-2H-pyran-3,4,5-triol (**40**)

Brown amorphous solid, R_f_ = 0.66 (3:7, methanol: ethyl acetate, v/v), ^1^H NMR (400 MHz, CD_3_OD): δ 7.92–7.87 (m, 1 H), 7.52–7.43 (m, 1 H), 7.35–7.33 (m, 1 H), 7.20 (dd, *J* = 2.0 Hz, *J* = 8.8 Hz, 1 H), 6.67 (s, 1 H), 3.87–3.84 (m, 1 H), 3.80–3.72 (m, 1 H), 3.53–3.48 (m, 1 H), 3.45–3.40 (m, 1 H), 3.20–3.12 (m, 2 H), 3.08–3.04 (m, 1 H), 2.86 (dd, *J* = 8.40, *J* = 14.8 Hz, 1 H), 2.28 (dd, *J* = 9.2 Hz, *J* = 14.4 Hz, 1 H); ^13^C NMR (100 MHz, CD_3_OD): δ 158.8, 155.0, 135.8, 130.9, 129.3, 126.2, 125.8, 122.1, 121.8, 118.8, 112.7, 111.0, 78.6, 78.2, 74.6, 74.5, 72.0, 71.4, 69.6, 69.5, 69.3, 61.3, 32.6. HRMS (ESI), m/z calcd for C_18_H_19_ClN_2_O_6_ [M + H]^+^ 395.1004; Found: 395.1044.

#### (2 S,3 S,4 R,5 S,6 R)-2-((5-(1H-indol-3-yl)isoxazol-3-yl)methyl)-6-(hydroxymethyl) tetrahydro-2H-pyran-3,4,5-triol (**41**)

Brown amorphous solid, R_f_ = 0.60 (3:7, methanol: ethyl acetate, v/v), ^1^H NMR (400 MHz, CD_3_OD): δ 7.48 (t, *J* = 7.6 Hz, 1 H), 7.34 (d, *J* = 8.4 Hz, 1 H), 7.25 (s, 1 H), 7.08 (t, *J* = 7.6 Hz, 1 H), 6.99 (t, *J* = 7.2 Hz, 1 H), 5.80 (t, *J* = 9.6 Hz, 1 H), 3.83–3.79 (m, 1 H), 3.76 (brs, 1 H), 3.72–3.66 (m, 1 H), 3.59–3.53 (m, 1 H), 3.50–3.45 (m,1 H), 3.43 (brs, 1 H), 3.37–3.30 (m, 1 H), 2.85 (dd, *J* = 9.6 Hz, *J* = 13.6 Hz, 1 H),2.41 (dd, *J* = 4.8 Hz, *J* = 14.8 Hz, 1 H); ^13^C NMR (100 MHz, CD_3_OD): δ 165.2, 158.3, 137.3, 125.3, 123.1, 122.9, 121.5, 120.5, 118.9, 118.8, 118.6, 118.5, 111.3, 80.6, 76.8, 76.7, 76.0, 75.5, 74.9, 71.2, 67.1, 61.4, 42.8, 42.4, 36.5. HRMS (ESI), *m/z* calcd for C_18_H_20_N_2_O_6_ [M + Na] ^+^ 383.1214; Found: 383.1209.

#### (2 R,3 S,4 R,5 S,6 S)-2-(hydroxymethyl)-6-((5-(5-nitro-1H-indol-3-yl)isoxazol-3-yl) methyl) tetrahydro-2H-pyran-3,4,5-triol (**42**)

Yellow amorphous solid, R_f_ = 0.61 (3:7, methanol: ethyl acetate, v/v), ^1^H NMR (400 MHz, CD_3_OD): δ 8.87 (d, *J* = 2.0 Hz, 1 H), 8.20 (s, 1 H), 8.04 (dd, *J* = 2.0 Hz, *J* = 8.8 Hz, 1 H), 7.52 (s, 1 H), 7.47 (d, *J* = 8.8 Hz, 1 H), 3.77 (s, 1 H), 3.73–3.71 (m, 1 H), 3.62 (d, *J* = 9.2 Hz, 1 H), 3.57 (d, *J* = 6.8 Hz, 1 H), 3.52–3.50 (m, 1 H), 3.42 (d, *J* = 7.2 Hz, 1 H), 3.39–3.37 (m, 1 H), 2.59 (dd, *J* = 8.0 Hz, *J* = 14.4 Hz, 1 H), 2.46 (dd, *J* = 5.6 Hz, *J* = 14.4 Hz, 1 H); ^13^C NMR (100 MHz, CD_3_OD): δ 161.8, 155.6, 141.0, 139.8, 126.9, 125.0, 117.0, 116.6, 111.1, 81.1, 80.6, 79.3, 75.9, 75.1, 71.3, 71.0, 69.4, 67.5, 67.2, 61.7, 61.2, 46.0, 42.9, 36.7. HRMS (ESI), *m/z* calcd for C_18_H_19_N_3_O_8_O_3_ [M + H]^+^ 406.1245; Found: 406.1239.

#### (2 S,3 S,4 R,5 S,6 R)-2-((5-(5-bromo-1H-indol-3-yl)isoxazol-3-yl)methyl)-6-(hydroxy methyl) tetrahydro-2H-pyran-3,4,5-triol (**43**)

Brown solid, R_f_ = 0.42 (3: 7, methanol: ethyl acetate, v/v), ^1^H NMR (400 MHz, CD_3_OD): δ 7.59 (d, *J* = 9.6 Hz, 1 H), 7.29 (s, 1 H), 7.26 (s, 1 H). 7.15 (d, *J* = 8.4 Hz, 1 H), 5.74 (s, 1 H), 3.79 (s, 1 H), 3.72–3.69 (m, 2 H), 3.60–3.56 (m, 1 H), 3.53–3.48 (m, 2 H), 3.15 (brs, 1 H), 2.86–2.76 (m, 1 H), 2.65–2.62 (m, 1 H); ^13^C NMR (100 MHz, CD_3_OD) δ 158.4, 135.9, 128.2, 127.0, 124.5, 124.2, 121.1, 113.9, 113.5, 112.0, 80.5, 76.4, 76.2, 75.5, 74.9, 71.2, 67.0, 61.2, 42.9, 42.5, 36.6. HRMS (ESI), *m/z* calcd for C_18_H_19_BrN_2_O_6_ [M + H]^+^ 441.0481; Found: 441.0626.

#### (2 S,3 S,4 R,5 S,6 R)-2-((5-(5-chloro-1H-indol-3-yl)isoxazol-3-yl)methyl)-6-(hydroxy methyl) tetrahydro-2H-pyran-3,4,5-triol (**44**)

Brown amorphous solid, R_f_ = 0.48 (3:7, methanol: ethyl acetate, v/v), ^1^H NMR (400 MHz, CD_3_OD): δ 7.87 (s, 1 H), 7.50 (dd, *J* = 2.0 Hz, 1 H), 7.36 (dd, *J* = 6.8 Hz, *J* = 8.4 Hz, 1 H), 7.10 (dd, *J* = 1.6 Hz, *J* = 8.8 Hz, 1 H), 5.81 (t, *J* = 10.0 Hz, 1 H), 3.83 (d, *J* = 3.6 Hz, 1 H), 3.80–3.79 (m, 1 H), 3.74 (d, *J* = 2.8 Hz, 1 H), 3.57–3.52 (m, 1 H), 3.48–3.44 (m, 1 H), 3.71–3.68 (m, 2 H), 2.57 (dd, *J* = 8.0 Hz, *J* = 14.4 Hz, 1 H), 2.43 (dd, *J* = 5.6 Hz, *J* = 14.4 Hz, 1 H); ^13^C NMR (100 MHz, CD_3_OD): δ 181.4, 159.2, 140.4, 134.8, 130.7, 130.5, 126.3, 119.4, 118.3, 112.1, 105.2, 80.0, 74.9, 74.1, 70.6, 67.1, 61.2, 48.8, 36.5. HRMS (ESI) *m/z* calcd for C_18_H_19_ClN_2_O_6_ [M + H]^+^ 419.0802; Found: 419.0975.

### Cell culture

The human breast cancer cell line MDA-MB-231 were cultured in Dulbecco’s Modified Eagle’s Medium (DMEM) (Gibco, St. Louis, USA) supplemented with 10% heat inactivated FBS (Fetal Bovine Serum, Gibco, USA) and 1% penicillin-streptomycin (Gibco Canada) in humidified CO_2_ incubator at 37 °C. The cells were maintained as a monolayer in 100 mm culture plate. The cells used for each experiment were of less than 8 passage number. Sub culturing of cells was performed every third day, followed by trypsin EDTA treatment.

### Cell viability assay

Cell viability assay was performed as per standard protocols. After 72 h of cell incubation in the presence or absence of each compound, cell viability was evaluated by using MTT ((3-(4,5-dimethylthiazol-2-yl)-2,5-diphenyltetrazolium bromide)) (Himedia Pvt. Ltd, India). In brief, this is a homogeneous, colorimetric method for determining the number of viable cells in proliferation, cytotoxicity or chemosensitivity assays. MTT is bio reduced by cells into a formazan product that is soluble in the tissue culture medium. After 72 h of compound treatment, the medium was removed and 100 μL of MTT (0.5 mg ml^−1^ in media) was added to the cells and kept in the dark for 3 h at 37 °C in the CO_2_ incubator and the formazan formed was dissolved in 100 μL DMSO (Dimethyl Sulfoxide). The absorbance of the formazan product at 595 nm was measured directly from 96-well assay plates without additional processing using a multimode plate reader (Bio-Rad) (iMark, India), as absorbance is directly proportional to the number of viable cells in culture. The percentage of viable cells in each group is determined with respect to the untreated control cells.

### LDH cytotoxicity assays

Cytotoxicity was assessed with an LDH-cytotoxicity detection kit (Hi media, CCK036), Assays was performed according to assay protocol. Compound was taken at 25 μM concentration. The % of Cytotoxicity was calculated by this formula.

% Cytotoxicity = 100 × A − C/B − C, Where, A = Average absorbance of test. Average absorbance of background control (or vehicle control if applicable), B = Average absorbance of maximum LDH control − Average absorbance of volume correction control C = Average absorbance of untreated control − Average absorbance of background control.

### Assay for cyclooxygenases (COXs) inhibition

Assay for cyclooxygenases (COXs) inhibition for COX-2 activity was determined by a fluorescence-based method using a COX-2 inhibitor screening assay kit (Bio vision, catalog # k547–100, S. millipitas Blvd, USA) in a total volume of 100 μL according to the directions provided with the kit manufacturer. The inhibitory activities of the tested compounds were based on the fluorometric detection of Prostaglandin G2 the intermediate product generated by the COX enzyme. The enzymes were preincubated for 5 min at 25 °C with the test compounds prior to addition of arachidonic acid. Celecoxib was used as positive control. Fluorescence was measured (Ex/Em = 540/590 nm) kinetically at 25 °C for 5–10 minutes. Two time point was chosen in the linear range of the plot and obtained the corresponding values (RFU1 and RFU2) The COX-2 inhibitory activity was calculated according to the equation. % Relative inhibition = Slope of EC − Slope of S/Slope of EC × 100. Where EC is the enzyme control and S is sample screen and slope of all the sample is calculated by dividing the net ΔRFU(RFU_2_ − RFU_1_) values by the ΔT (T_2_ − T_1_).

## Supplementary information

Supplementary information.
