# Publisher Correction: Design and efficient synthesis of pyrazoline and isoxazole bridged indole *C*-glycoside hybrids as potential anticancer agents

**DOI:** 10.1038/s41598-020-67068-5

**Published:** 2020-06-17

**Authors:** Priti Kumari, Vishnu S. Mishra, Chintam Narayana, Ashish Khanna, Anindita Chakrabarty, Ram Sagar

**Affiliations:** 1 Department of Chemistry, School of Natural Sciences, Shiv Nadar University (SNU), NH91, Tehsil-Dadri, Gautam Buddha Nagar, Uttar Pradesh 201314 India; 2 Department of Life Sciences, School of Natural Sciences, Shiv Nadar University (SNU), NH91, Tehsil-Dadri, Gautam Buddha Nagar, Uttar Pradesh 201314 India; 3 Department of Chemistry, Institute of Science, Banaras Hindu University, Varanasi, Uttar Pradesh 221005 India

Correction to: *Scientific Reports* 10.1038/s41598-020-63377-x, published online 20 April 2020

This Article contains an incorrect version of Scheme 1 and Scheme 2. The correct version of Scheme 1 and Scheme 2 appears below.

**Scheme 1:**
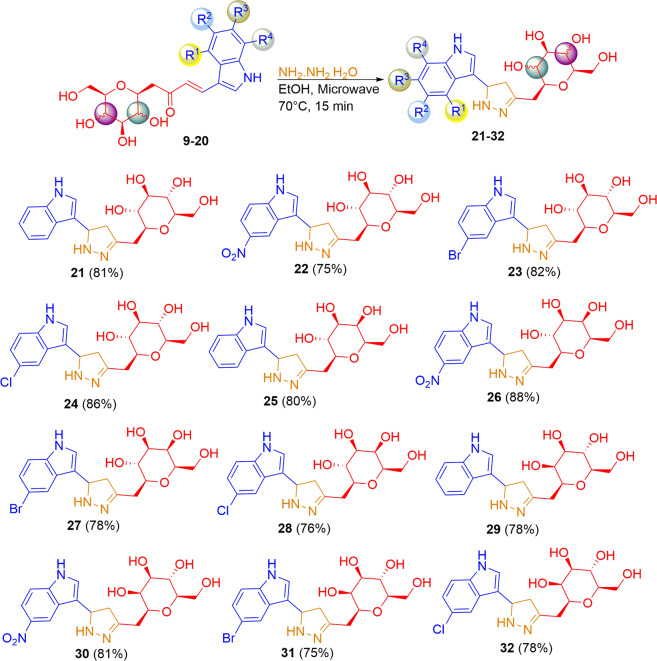
.

**Scheme 2:**
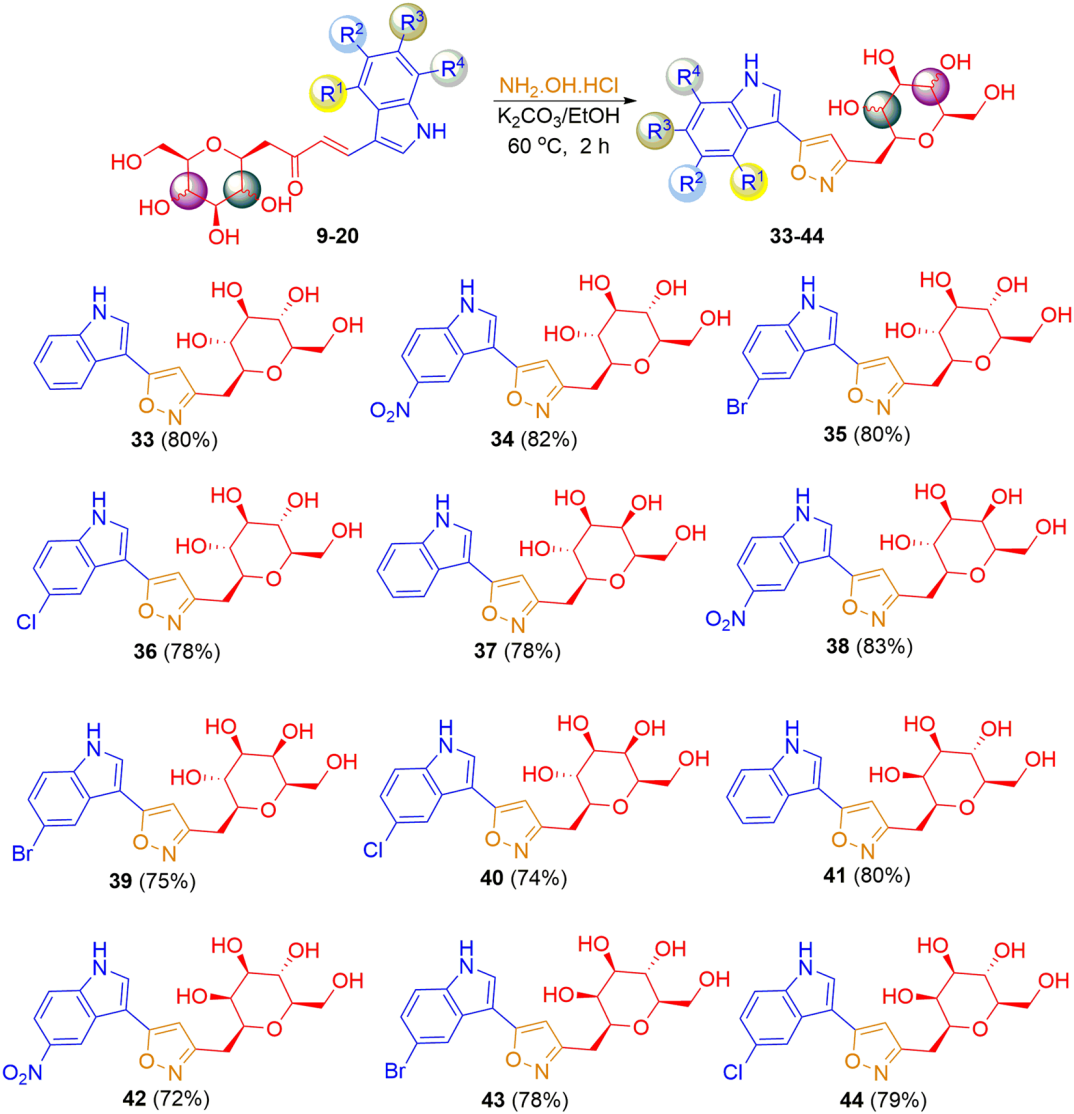
.

