# Supplementary material for: Design and efficient synthesis of pyrazoline and isoxazole bridged indole C-glycoside hybrids as potential anticancer agents
Source: Sci Rep. 2020 Apr 20;10:6660. doi: 10.1038/s41598-020-63377-x (PMC7170901; doi:10.1038/s41598-020-63377-x)
Supplement: Supplementary file 1 — Supplementary information. [file 41598_2020_63377_MOESM1_ESM.pdf]

Supplementary Information

Design and efficient synthesis of pyrazoline and isoxazole bridged indoles *C*-glycoside hybrids as potential anticancer agents

Priti Kumari,[1] Vishnu S. Mishra,[2] Chintam Narayana,[1] Ashish Khanna,[3] Anindita Chakrabarty[2] and Ram Sagar*[1,3]

[1]Department of Chemistry, School of Natural Sciences, Shiv Nadar University (SNU), NH91, Tehsil-Dadri, Gautam Buddha Nagar, Uttar Pradesh, 201314, India.
[2]Department of Life Sciences, School of Natural Sciences, Shiv Nadar University (SNU), NH91, Tehsil-Dadri, Gautam Buddha Nagar, Uttar Pradesh, 201314, India.
[3]Department of Chemistry, Institute of Science, Banaras Hindu University, Varanasi, Uttar Pradesh, 221005, India.

***E-mail (Corresponding author): ram.sagar@bhu.ac.in**

S. No.	Contents	Page No.
1	Synthesis and spectral data of compounds **1-3**	S2-S3
2	Copies of ^{1}H NMR and ^{13}C NMR of compounds **1-3**	S4-S6
3	Copies of ^{1}H NMR and ^{13}C NMR of indole linked α-β-unsaturated ketones **9-20**	S7-S18
3	Copies of ^{1}H NMR and ^{13}C NMR of pyrazoline and isoxazole bridged indoles *C*-glycoside **21-44**	S19-S42
4	Details of LDH Assays	S43-S45
5	Table S1 representing H-bonding interactions with active compounds	S45

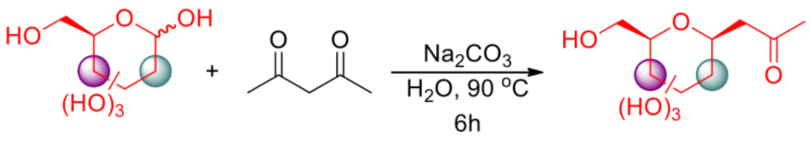

Scheme S1. Synthesis of β-*C*-glycosides **1-3**

1. ***General Experimental Procedure for the Synthesis of β-selective C-Glycoside 1-3***: To a 100 mL of round bottom flask, **1a** (10 gm, 55.5 mmol) and sodium carbonate (5.88 gm, 55.5 mmol) was taken in water (100 ml) followed by addition of acetylacetone (6.88 ml, 66.6 mmol) and heated at 90 °C. The progress of reaction was monitored by TLC (R_f = 0.56, 3: 7 = methanol: ethyl acetate, v/v). After completion, reaction mixture was passed through acidic resin and washed by dichloromethane. The aqueous layer concentrated on rotary evaporator to get crude product. The crude product was purified by flash column chromatography which furnished the designed product **1** in 93% yield (11.36 gm) as sticky white solid. Similar reaction protocol has been followed for preparation of compound **2** and **3.**

*1.1. 1-((2S,3R,4R,5S,6R)-3,4,5-trihydroxy-6-(hydroxymethyl)tetrahydro-2H-pyran-2-yl) propan-2-one (**1**)*: White Sticky solid Rf = 0.5 (3:7, methanol: ethyl acetate), ^{1}H NMR (400 MHz, D_2O): δ 3.86 (dd, *J* = 1.6 Hz, *J* = 10.8 Hz, 1H, H-1), 3.80 (td, *J* = 3.2 Hz, *J* = 12.4 Hz, 1H, H-5), 3.68 (dd, *J* = 5.2 Hz, *J* = 12 Hz, 1H, H-6a), 3.50 (t, *J* = 8.8 Hz, 1H, H-6b), 3.433.40 (m, 1H, H-4), 3.37 (m, 1H, H-3), 3.24 (t, *J* = 9.2Hz, 1H, H-2), 3.03 (dd, *J* = 2.8 Hz, *J* = 16.4 Hz, 1H, H-1a), 2.73 (dd, *J* = 9.2 Hz, *J* = 16.8 Hz, 1H, H-1b), 2.28 (s, 3H, CH_3); ^{13}C NMR (100 MHz, D_2O): δ 213.2 (C-2'), 79.5, 77.2, 75.2, 73.0, 69.7, 60.7, 45.6, 29.8. HRMS (ESI), *m/z* calcd for $C_9H_{16}O_6$ $[M+Na]^+$ 243.0839; found: 243.0861.

*1.2. 1-((2S,3R,4R,5R,6R)-3,4,5-trihydroxy-6-(hydroxymethyl)tetrahydro-2H-pyran-2-yl) propan-2-one (**2**)*: White Sticky solid, Rf= 0.45 (3:7, methanol: ethyl acetate, v/v), ^{1}H NMR (400 MHz, D_2O): δ 3.97 (d, *J* = 3.2 Hz, 1H, H-1), 3.74-3.70 (m, 1H, H-5), 3.67 (m, 1H, H-6), 3.64 (dd, *J* = 3.2 Hz, 1H, H-4), 3.46 (t, *J* = 9.6 Hz, 1H, H-2), 3.35 (brs, 1H, H-3), 3.03 (dd, *J* = 2.8 Hz, *J* = 16.8 Hz), 2.75 (dd, *J* = 9.2 Hz, *J* = 16.8 Hz), 2.28 (s, 3H, CH_3); ^{13}C NMR (100 MHz, D_2O): δ 213.4, 78.5, 75.7, 73.8, 70.4, 69.1, 61.2, 45.8, 29.8. HRMS (ESI), *m/z* calcd for $C_9H_{16}O_6$ $[M+H]^+$ 243.0839; found: 243.0861.

*1.3. 1-((2S,3S,4R,5S,6R)-3,4,5-trihydroxy-6-(hydroxymethyl)tetrahydro-2H-pyran-2-yl) propan-2-one (**3**):* Off white solid, Rf = 0.4 (3:7, methanol: ethyl acetate, v/v), ^{1}H NMR (400 MHz, CD_3OD): δ 3.92 (m,1H), 3.81 (dd, *J* = 2.4 Hz, 11.6 Hz, 1H), 3.74 (d, *J* = 2.8 Hz), 3.66 (dd, *J* = 5.6 Hz, *J* = 12 Hz, 1H), 3.57-3.52 (m, 1H), 3.50 (dd, *J* = 3.2 Hz, *J* = 9.2 Hz, 1H), 3.22-318 (m,1H), 2.89 (dd, *J* = 7.6 Hz, *J* = 17.2 Hz), 2.72 (dd, *J* = 5.6 Hz, *J* = 16.8 Hz, 1H), 2.19 (s, 3H, CH_3) ^{13}C NMR (100 MHz, CD_3OD): δ 208.2, 80.6, 74.9, 74.1, 71.0, 67.1, 61.5, 44.2, 29.1. HRMS (ESI) *m/z* calcd for $C_9H_{16}O_6$ $[M+K]^+$; 259.0578; found: 259.0601.

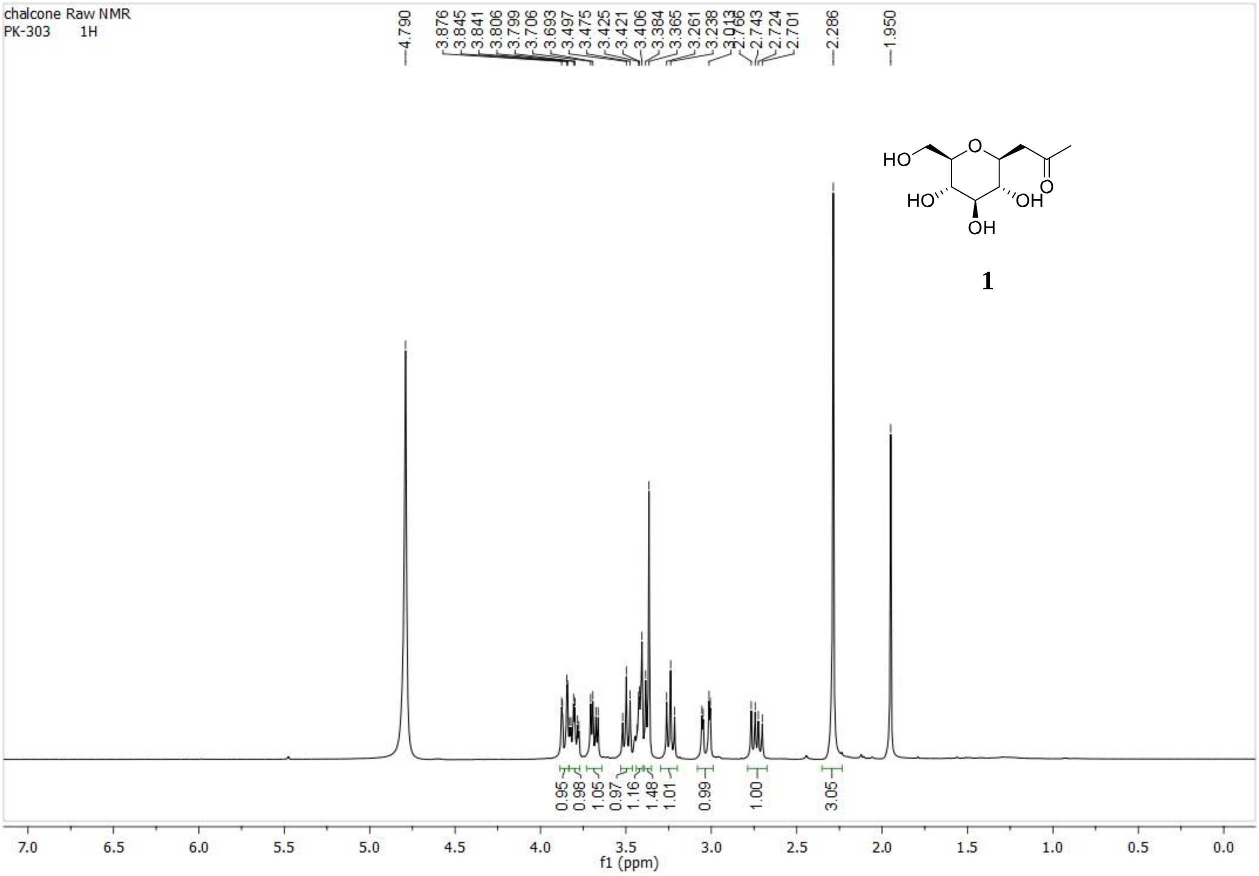
chalcone Raw NMR
PK-303 1H
4.790
3.876
3.845
3.841
3.806
3.799
3.706
3.693
3.497
3.475
3.425
3.421
3.406
3.384
3.365
3.261
3.238
3.013
2.766
2.743
2.724
2.701
2.286
1.950
HO
O
HO
OH
OH
O
1
0.95
0.98
1.05
0.97
1.16
1.48
1.01
0.99
1.00
3.05
7.0 6.5 6.0 5.5 5.0 4.5 4.0 3.5 3.0 2.5 2.0 1.5 1.0 0.5 0.0
f1 (ppm)

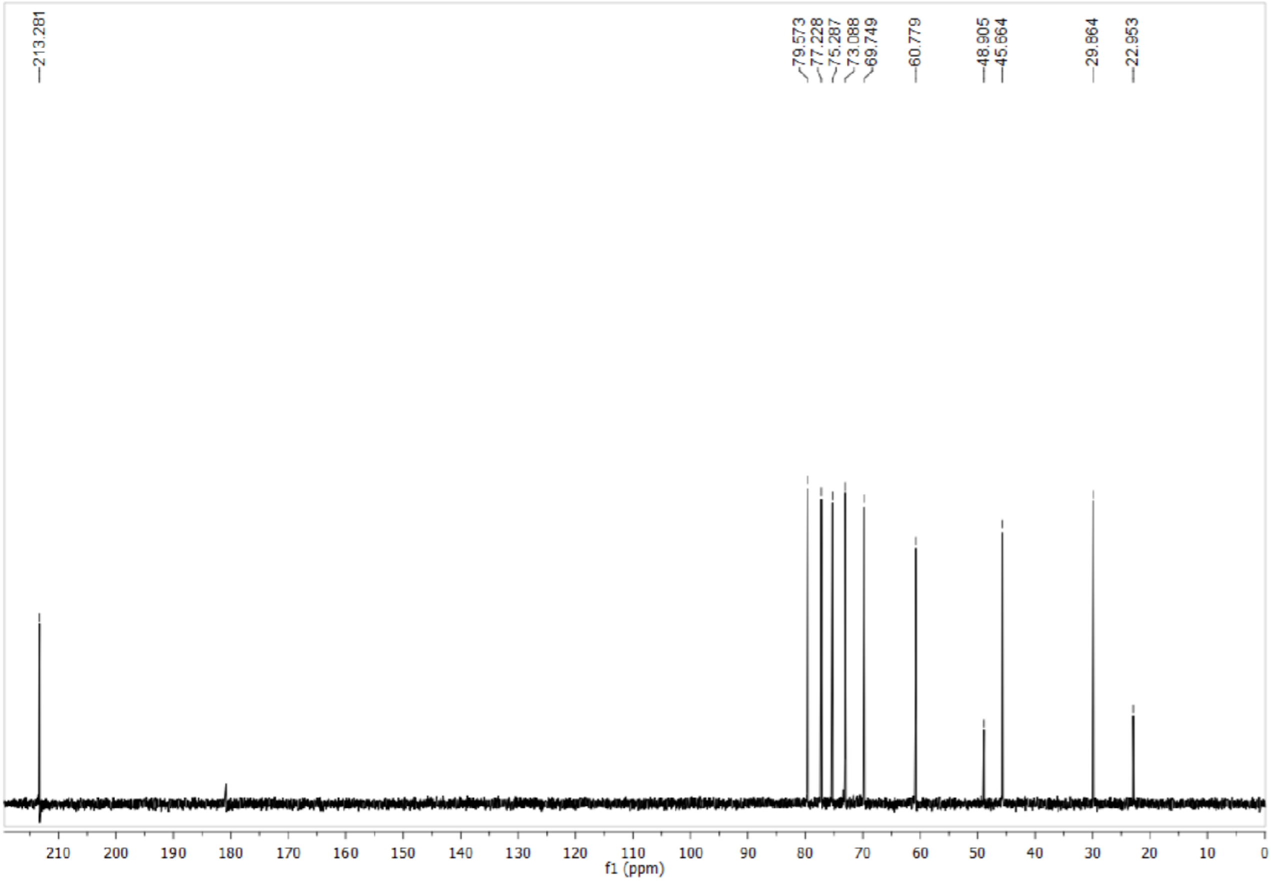
213.281
79.573
77.228
75.287
73.088
69.749
60.779
48.905
45.664
29.864
22.953
210 200 190 180 170 160 150 140 130 120 110 100 90 80 70 60 50 40 30 20 10 0
f1 (ppm)

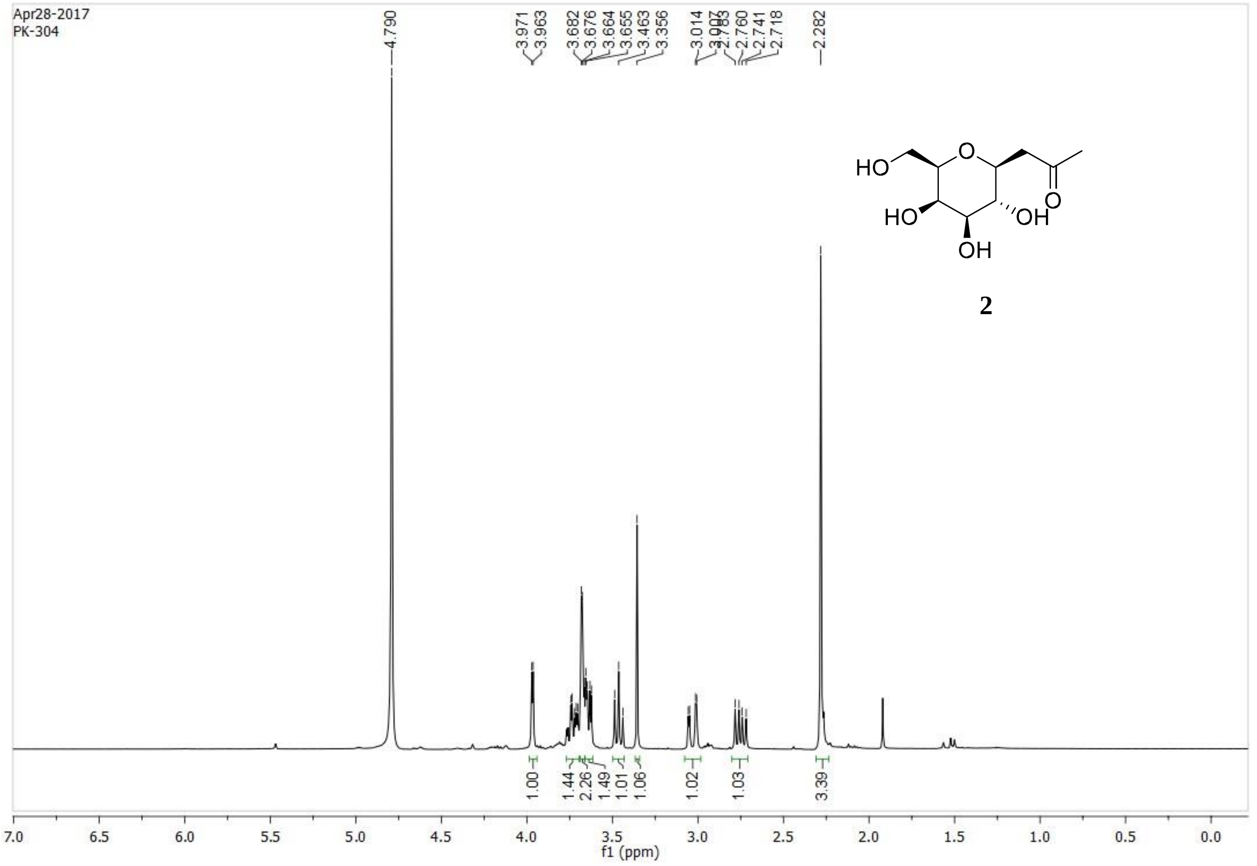

Apr28-2017
PK-304
4.790
3.971
3.963
3.682
3.676
3.664
3.655
3.463
3.356
3.014
2.760
2.741
2.718
2.282
HO
O
O
HO
OH
OH
2
1.00
1.44
2.26
1.49
1.01
1.06
1.02
1.03
3.39
7.0 6.5 6.0 5.5 5.0 4.5 4.0 3.5 3.0 2.5 2.0 1.5 1.0 0.5 0.0
f1 (ppm)

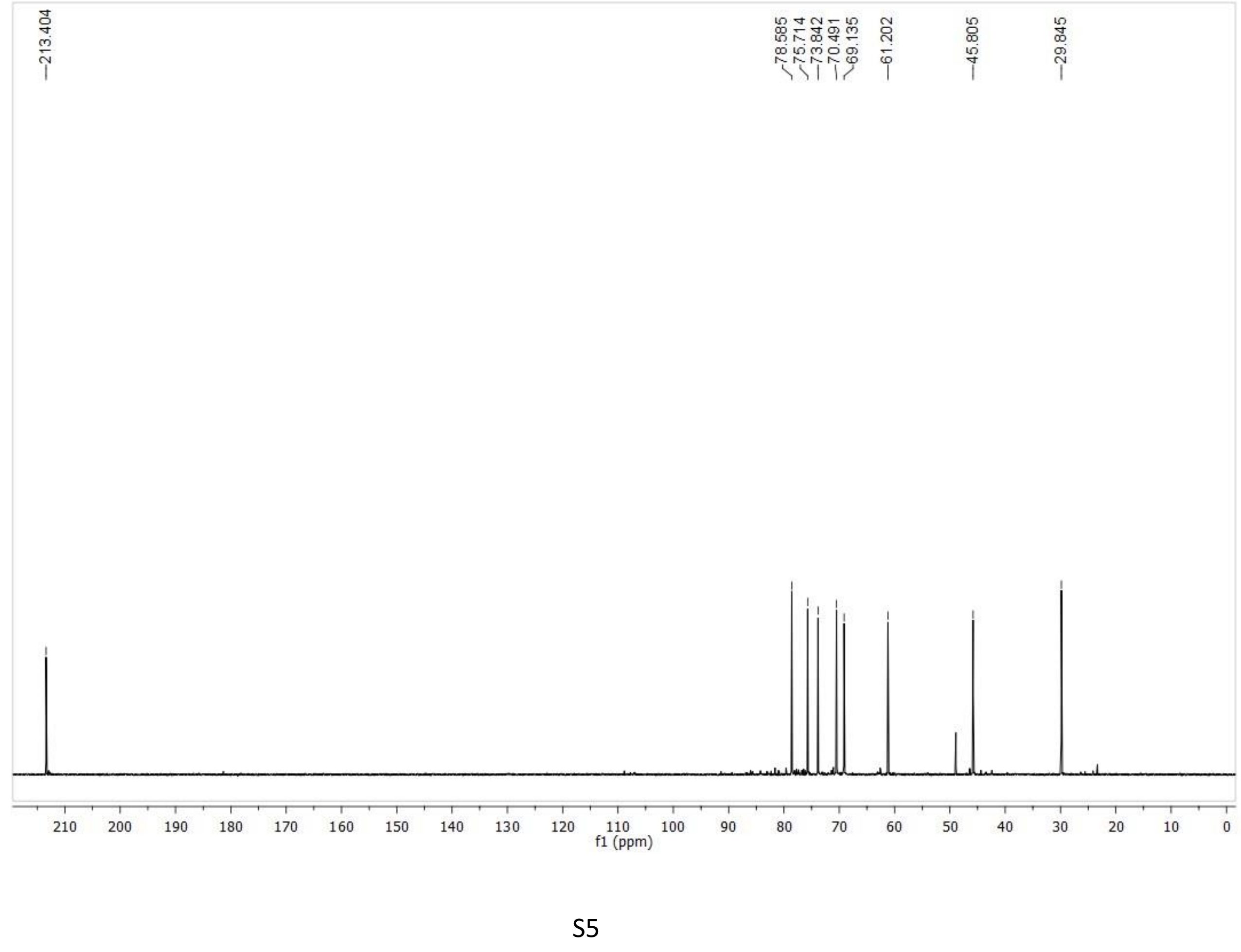

213.404
78.585
75.714
73.842
70.491
69.135
61.202
45.805
29.845
210 200 190 180 170 160 150 140 130 120 110 100 90 80 70 60 50 40 30 20 10 0
f1 (ppm)

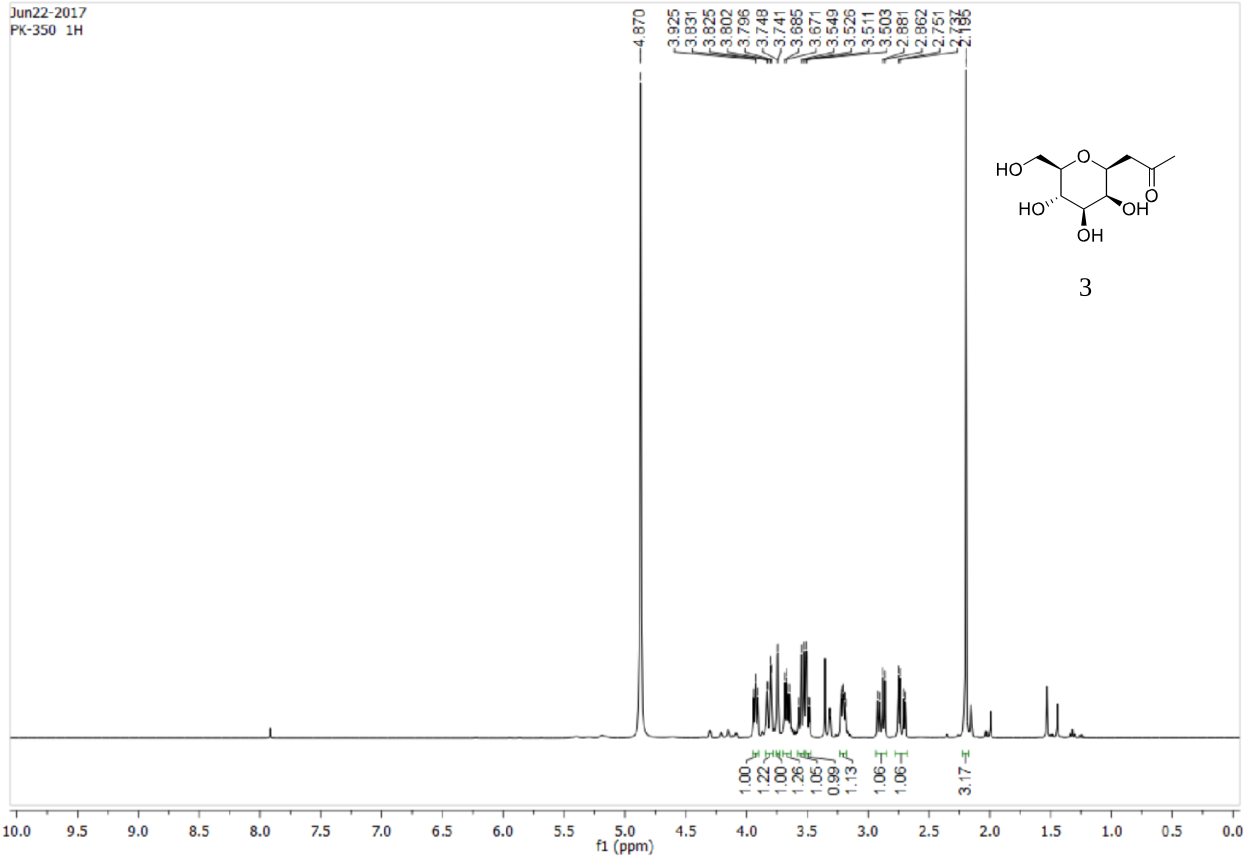
Jun22-2017
PK-350 1H
4.870
3.925
3.831
3.825
3.802
3.796
3.748
3.741
3.685
3.671
3.549
3.526
3.511
3.503
2.881
2.862
2.751
2.737
2.195
HO
O
HO
OH
OH
O
3
1.00
1.22
1.00
1.26
1.05
0.99
1.13
1.06
1.06
3.17
10.0
9.5
9.0
8.5
8.0
7.5
7.0
6.5
6.0
5.5
5.0
4.5
4.0
3.5
3.0
2.5
2.0
1.5
1.0
0.5
0.0
f1 (ppm)

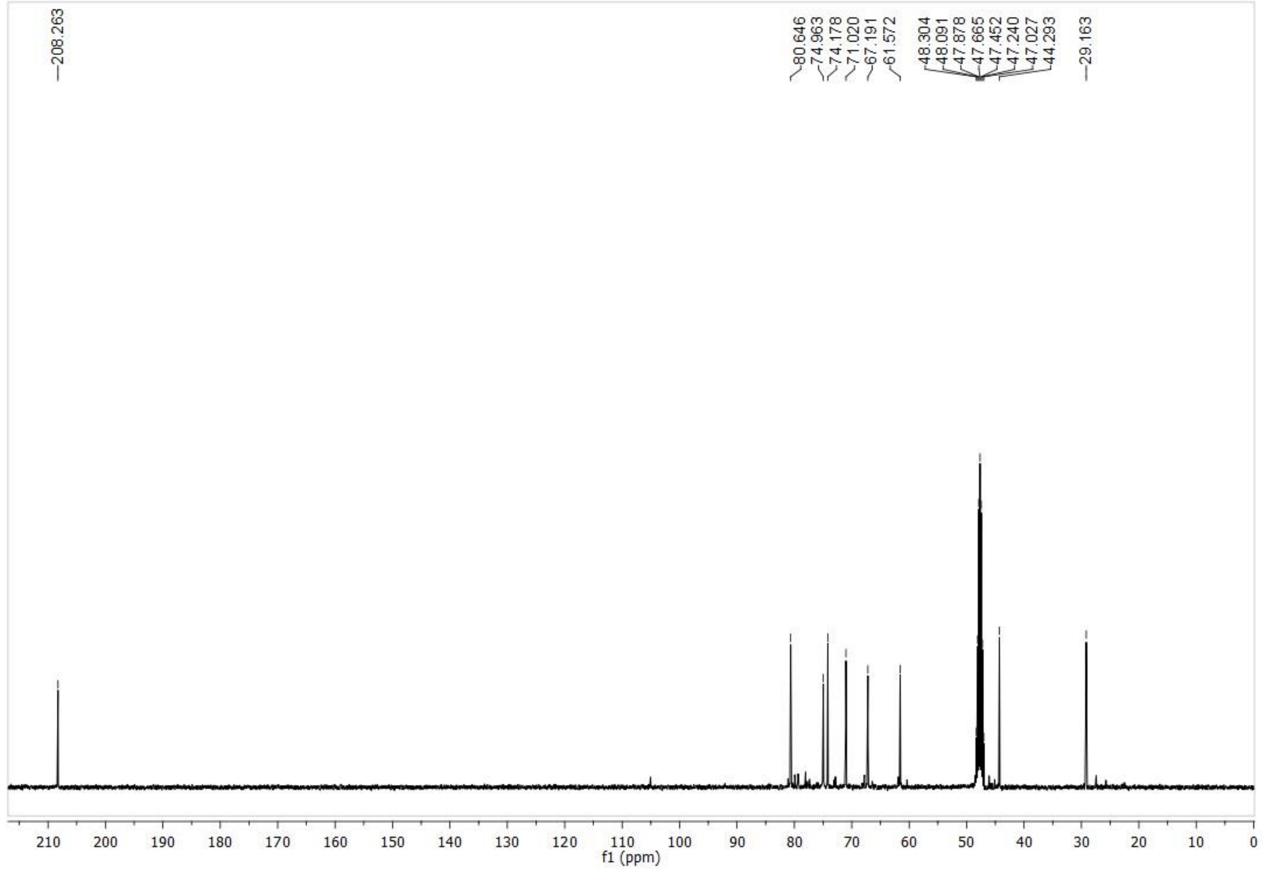
208.263
80.646
74.963
74.178
71.020
67.191
61.572
48.304
48.091
47.878
47.665
47.452
47.240
47.027
44.293
29.163
210
200
190
180
170
160
150
140
130
120
110
100
90
80
70
60
50
40
30
20
10
0
f1 (ppm)

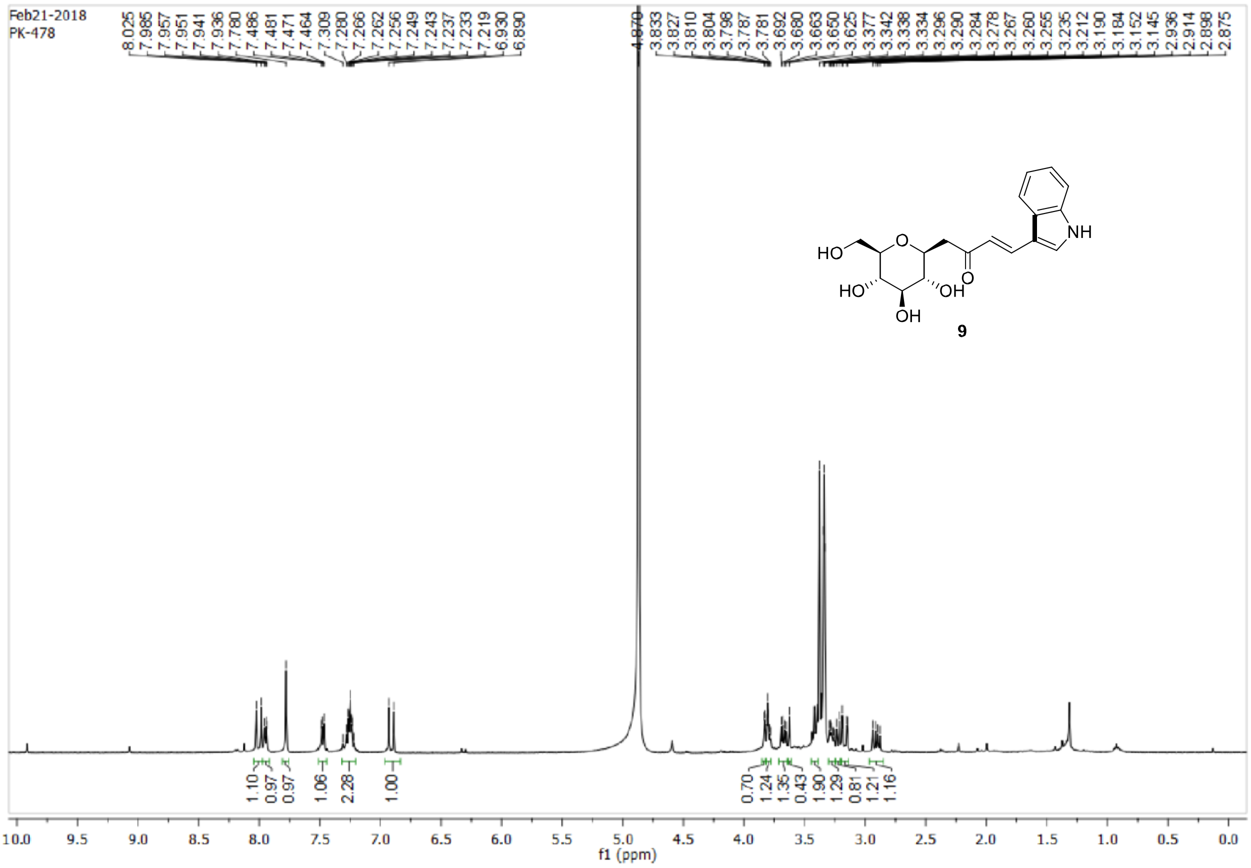
Feb21-2018
PK-478
8.025
7.985
7.957
7.951
7.941
7.936
7.780
7.486
7.481
7.471
7.464
7.309
7.280
7.266
7.262
7.256
7.249
7.243
7.237
7.233
7.219
6.930
6.890
4.870
3.833
3.827
3.810
3.804
3.798
3.787
3.781
3.692
3.680
3.663
3.650
3.625
3.377
3.342
3.338
3.334
3.296
3.290
3.284
3.278
3.267
3.260
3.255
3.235
3.212
3.190
3.184
3.152
3.145
2.936
2.914
2.898
2.875
HO
HO
OH
OH
O
O
NH
9
1.10
0.97
0.97
1.06
2.28
1.00
0.70
1.24
1.35
0.43
1.90
1.29
0.81
1.21
1.16
f1 (ppm)

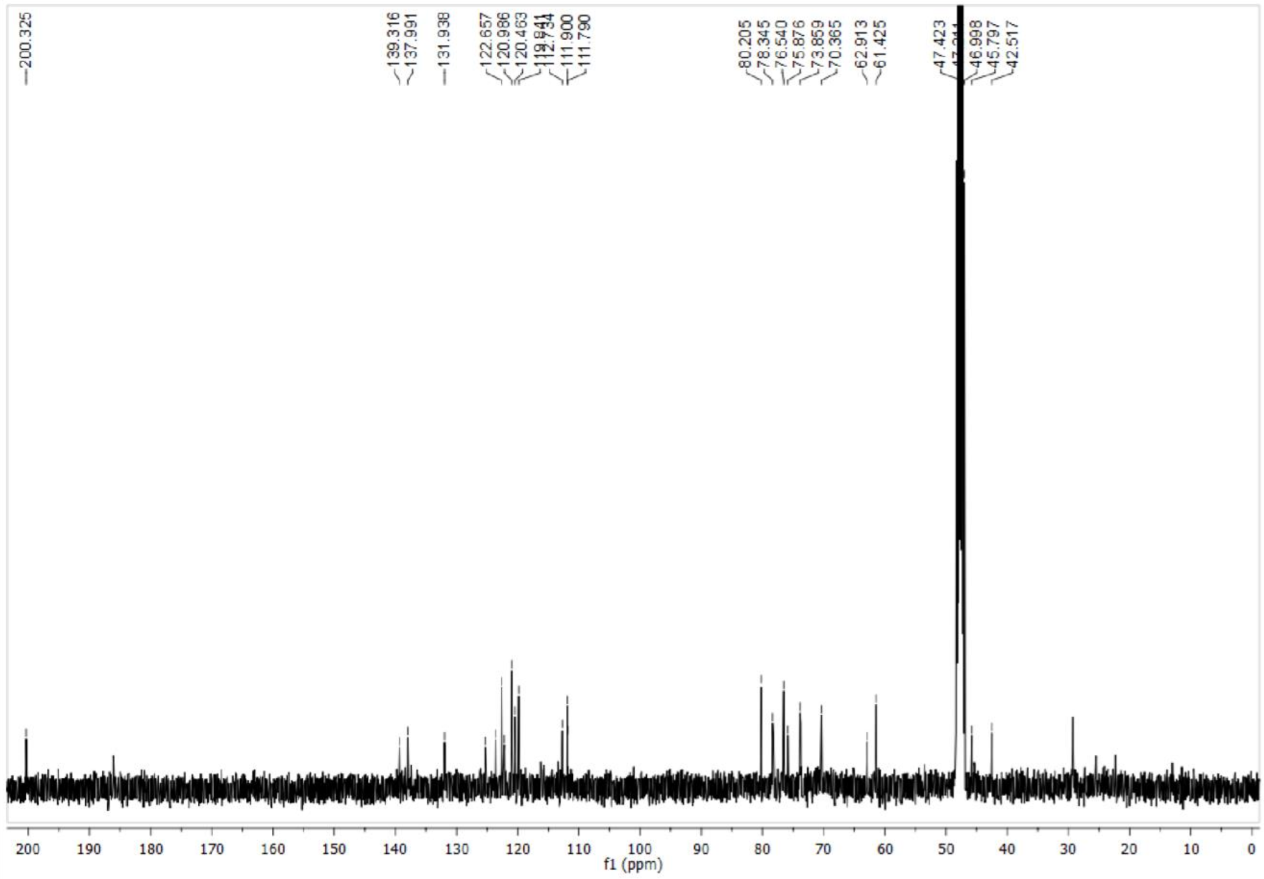
200.325
139.316
137.991
131.938
122.657
120.986
120.463
111.900
111.790
80.205
78.345
76.540
75.876
73.859
70.365
62.913
61.425
47.423
46.998
45.797
42.517
f1 (ppm)

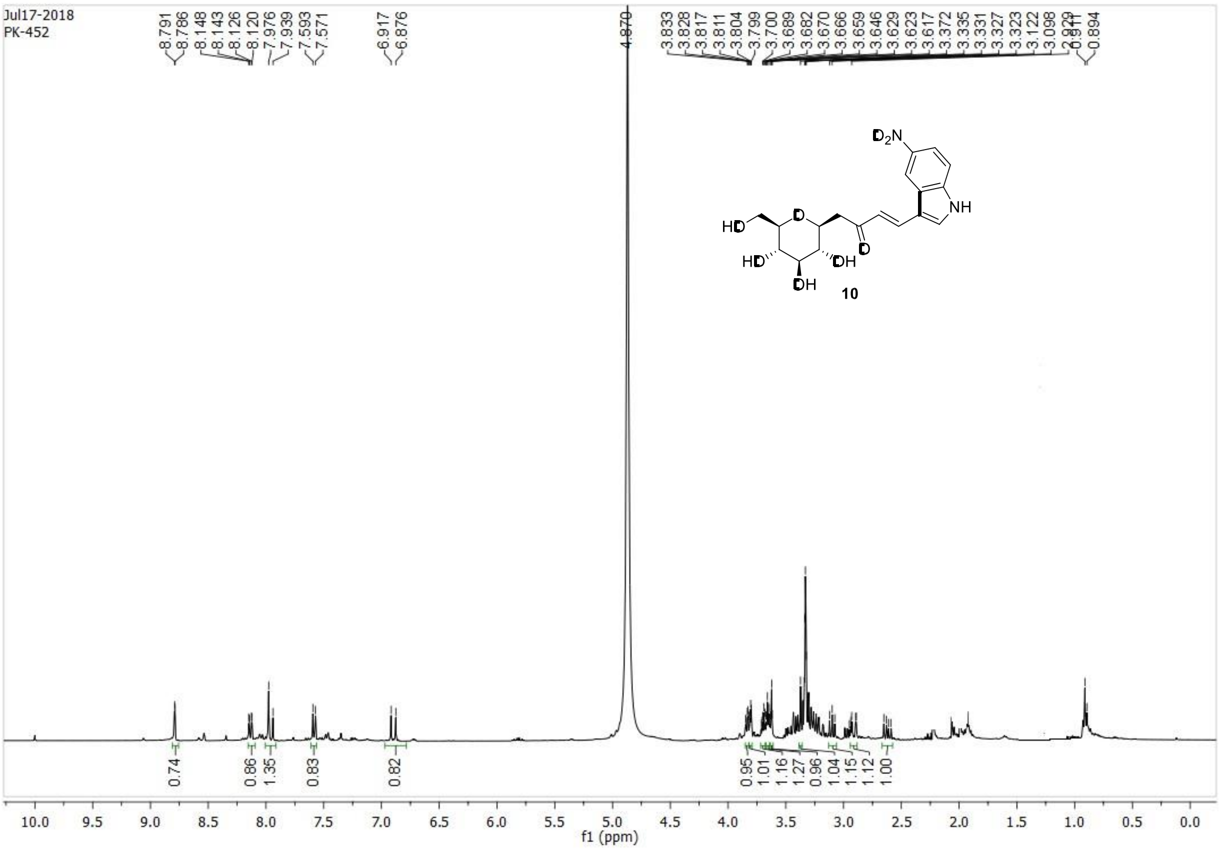
Jul17-2018
PK-452
8.791
8.786
8.148
8.143
8.126
8.120
7.976
7.939
7.593
7.571
6.917
6.876
4.870
3.833
3.828
3.817
3.811
3.804
3.799
3.700
3.689
3.682
3.670
3.666
3.659
3.646
3.629
3.623
3.617
3.372
3.335
3.331
3.327
3.323
3.122
3.098
0.911
0.894
10
0.74
0.86
1.35
0.83
0.82
0.95
1.01
1.16
1.27
0.96
1.04
1.15
1.12
1.00
f1 (ppm)

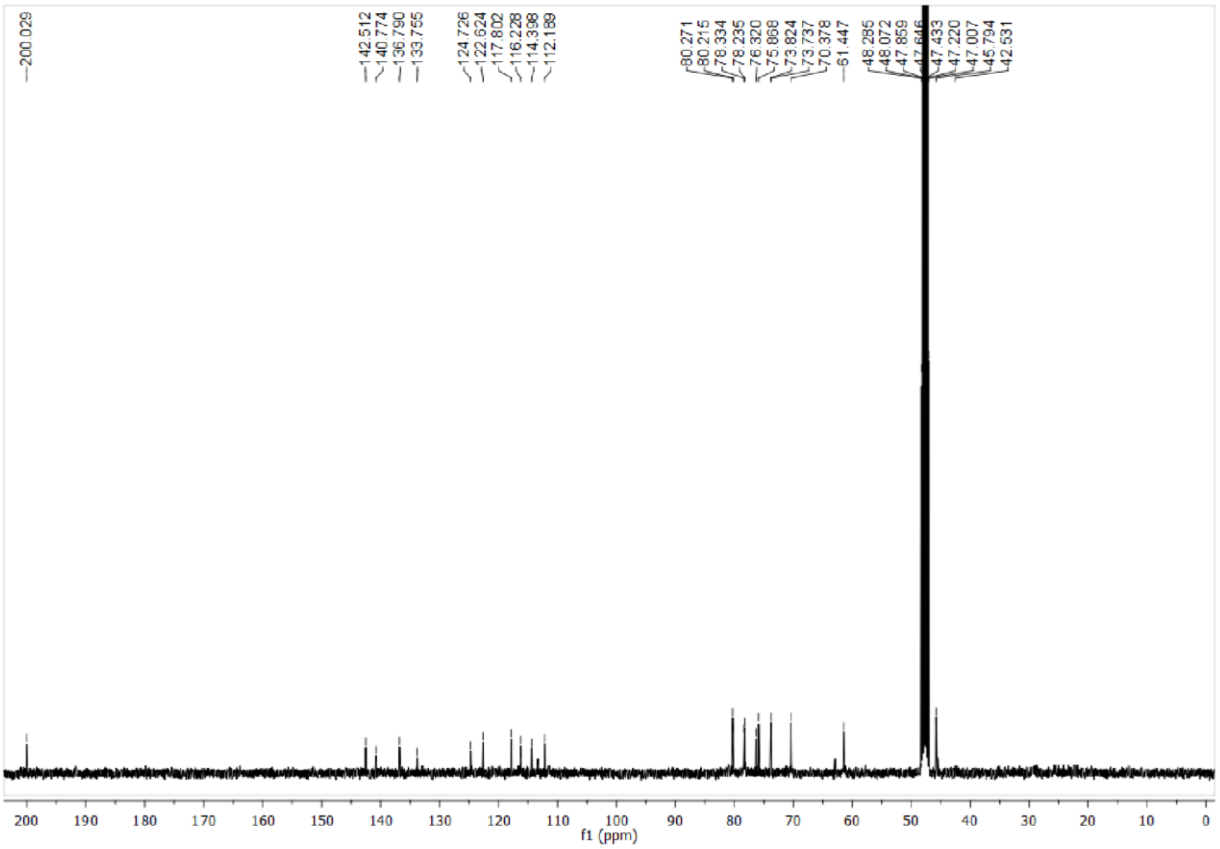
200.029
142.512
140.774
136.790
133.755
124.726
122.624
117.802
116.228
114.398
112.189
80.271
80.215
78.334
78.235
76.320
75.868
73.824
73.737
70.378
61.447
48.285
48.072
47.859
47.433
47.220
47.007
45.794
42.531
f1 (ppm)

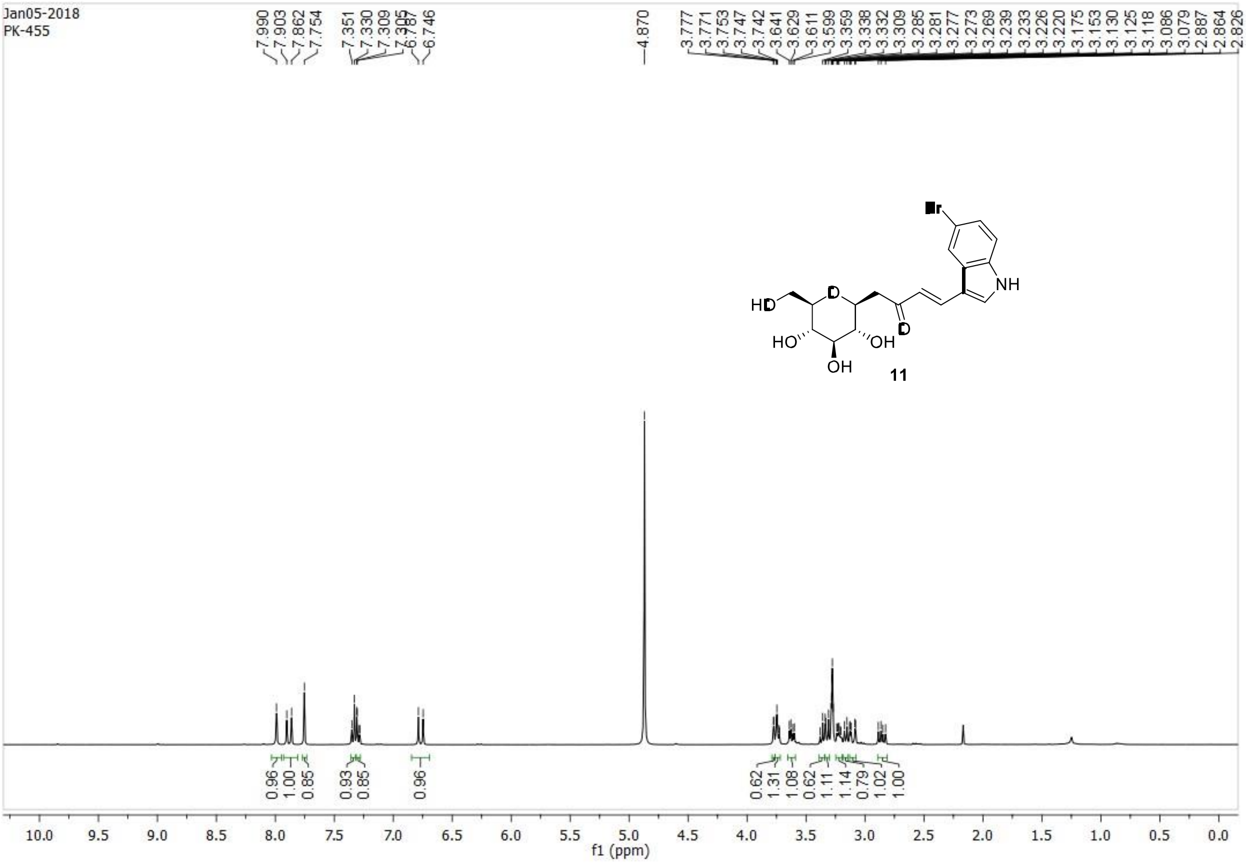

Jan05-2018
PK-455
7.990
7.903
7.862
7.754
7.351
7.330
7.309
7.305
6.787
6.746
4.870
3.777
3.771
3.753
3.747
3.742
3.641
3.629
3.611
3.599
3.359
3.338
3.332
3.309
3.285
3.281
3.277
3.273
3.269
3.239
3.233
3.226
3.220
3.175
3.153
3.130
3.125
3.118
3.086
3.079
2.887
2.864
2.826
HO
O
NH
HO
OH
OH
O
11
0.96
1.00
0.85
0.93
0.85
0.96
0.62
1.31
1.08
0.62
1.11
1.14
0.79
1.02
1.00
f1 (ppm)

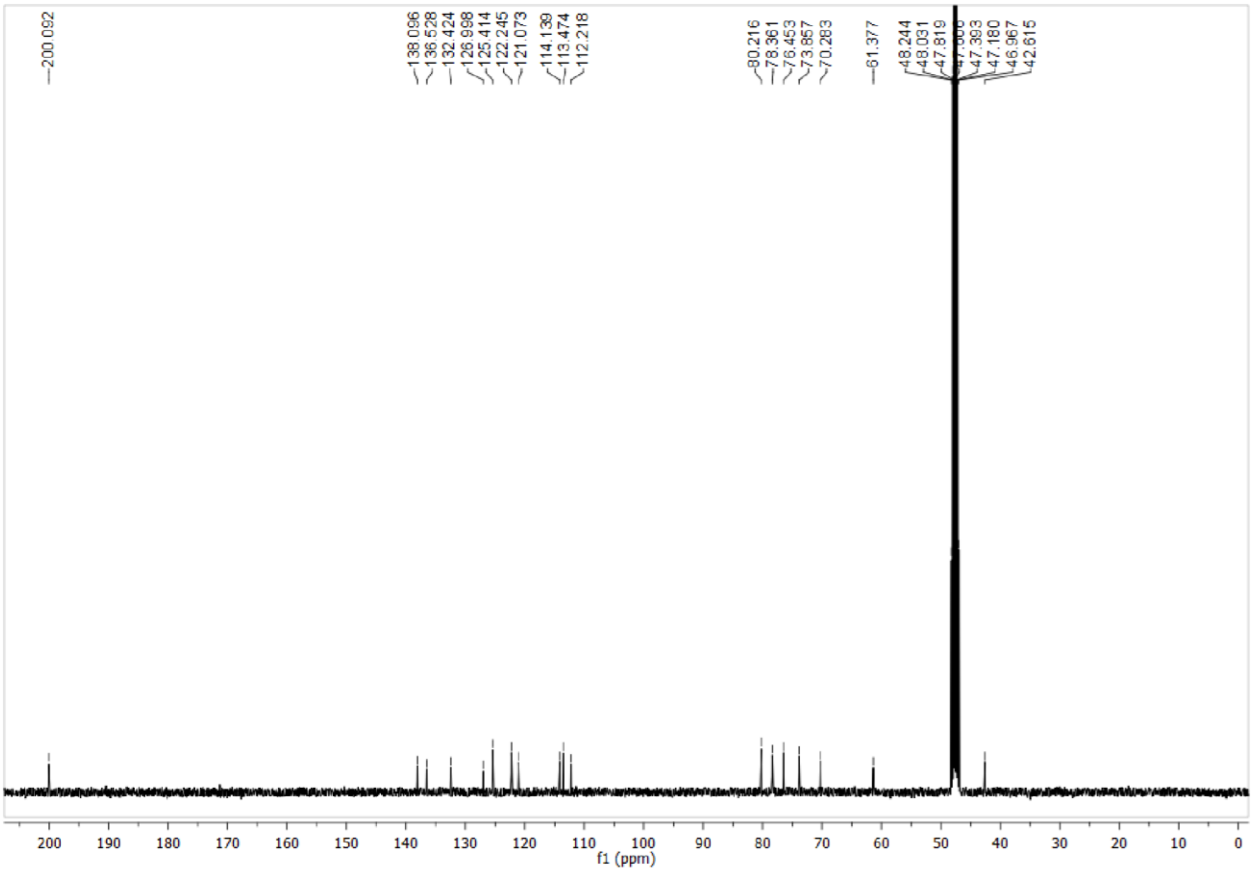

200.092
138.096
136.528
132.424
126.998
125.414
122.245
121.073
114.139
113.474
112.218
80.216
78.361
76.453
73.857
70.283
61.377
48.244
48.031
47.819
47.393
47.180
46.967
42.615
f1 (ppm)

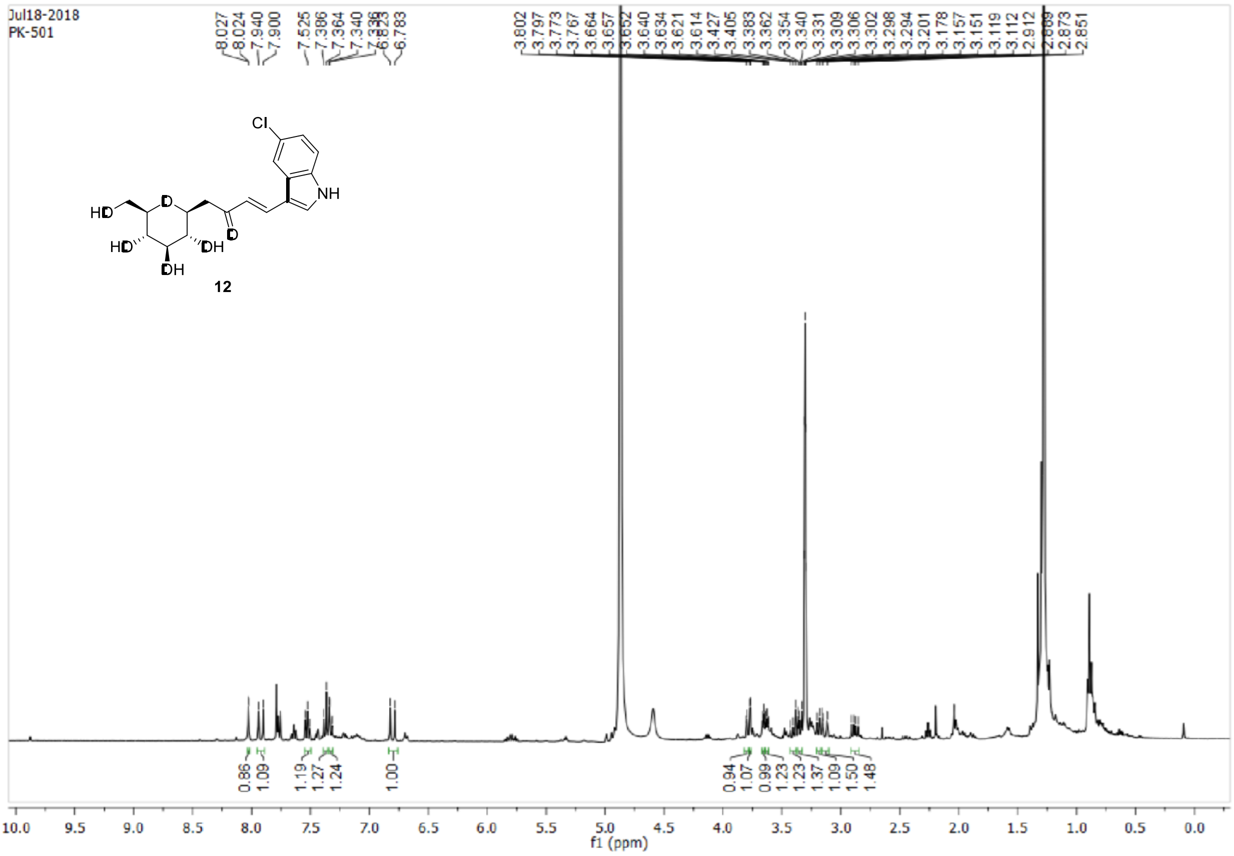
Jul18-2018
PK-501
Cl
NH
HO
O
OH
12
8.027
8.024
7.940
7.900
7.525
7.386
7.364
7.340
6.823
6.783
3.802
3.797
3.773
3.767
3.664
3.657
3.652
3.640
3.634
3.621
3.614
3.427
3.405
3.383
3.362
3.354
3.340
3.331
3.309
3.306
3.302
3.298
3.294
3.201
3.178
3.157
3.151
3.119
3.112
2.912
2.873
2.851
0.86
1.09
1.19
1.27
1.24
1.00
0.94
1.07
0.99
1.23
1.23
1.37
1.09
1.50
1.48
f1 (ppm)

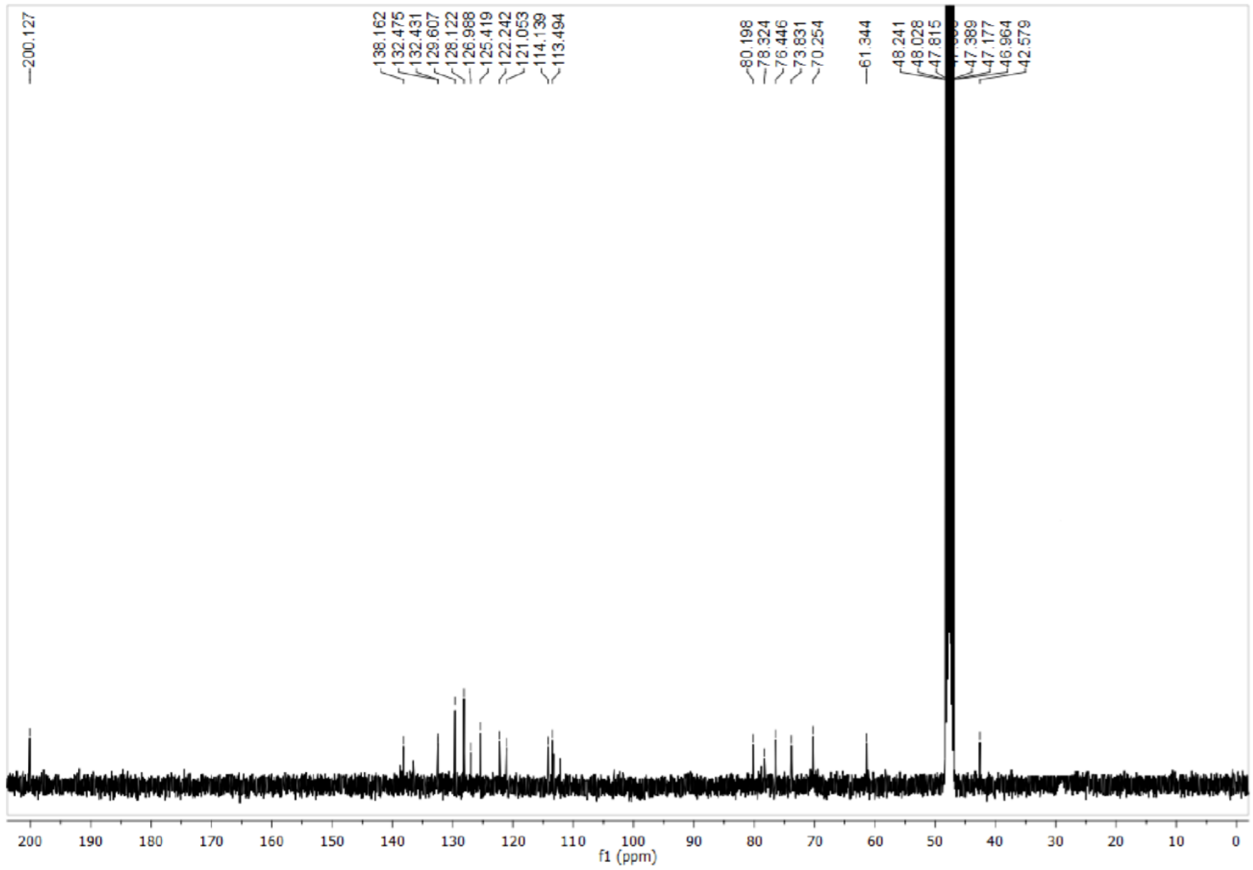
200.127
138.162
132.475
132.431
129.607
128.122
126.988
125.419
122.242
121.053
114.139
113.494
80.198
78.324
76.446
73.831
70.254
61.344
48.241
48.028
47.815
47.389
47.177
46.964
42.579
f1 (ppm)

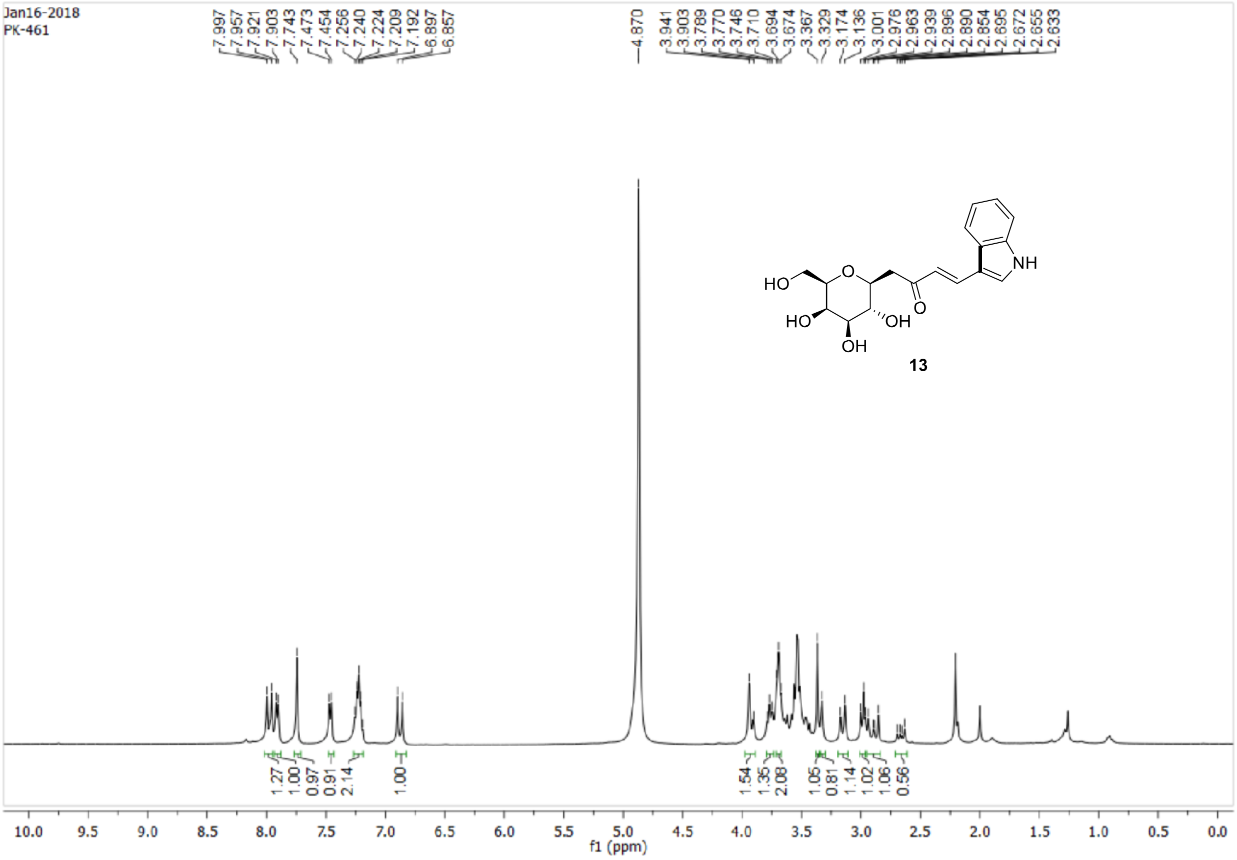
Jan16-2018
PK-461
7.997 7.957 7.921 7.903 7.743 7.473 7.454 7.256 7.240 7.224 7.209 7.192 6.897 6.857
4.870
3.941 3.903 3.789 3.770 3.746 3.710 3.694 3.674 3.367 3.329 3.174 3.136 3.001 2.976 2.963 2.939 2.896 2.890 2.854 2.695 2.672 2.655 2.633
1.27 1.00 0.97 0.91 2.14 1.00
1.54 1.35 2.08 1.05 0.81 1.14 1.02 1.06 0.56
HO
O
HO
OH
OH
O
NH
13
f1 (ppm)

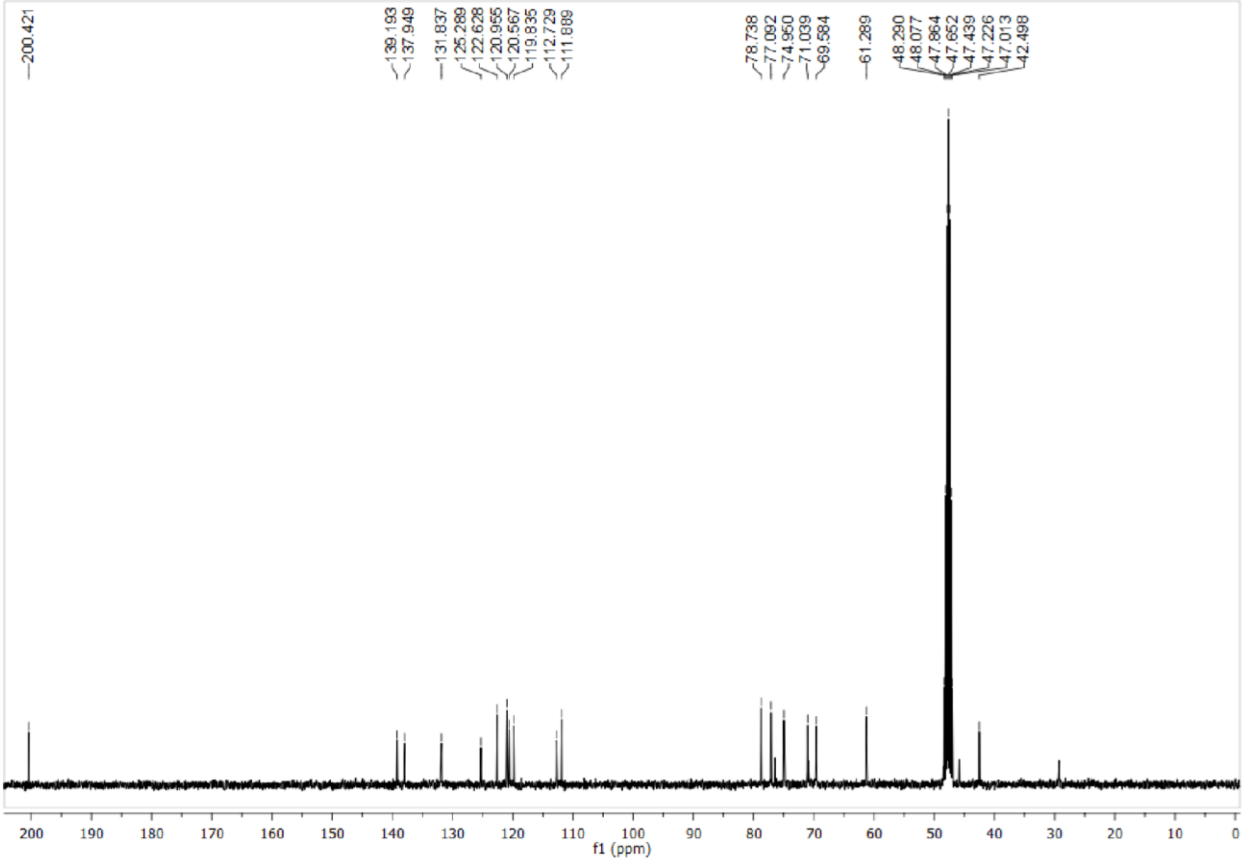
200.421
139.193 137.949 131.837 125.289 122.628 120.955 120.567 119.835 112.729 111.889
78.738 77.092 74.950 71.039 69.584
61.289
48.290 48.077 47.864 47.652 47.439 47.226 47.013 42.498
f1 (ppm)

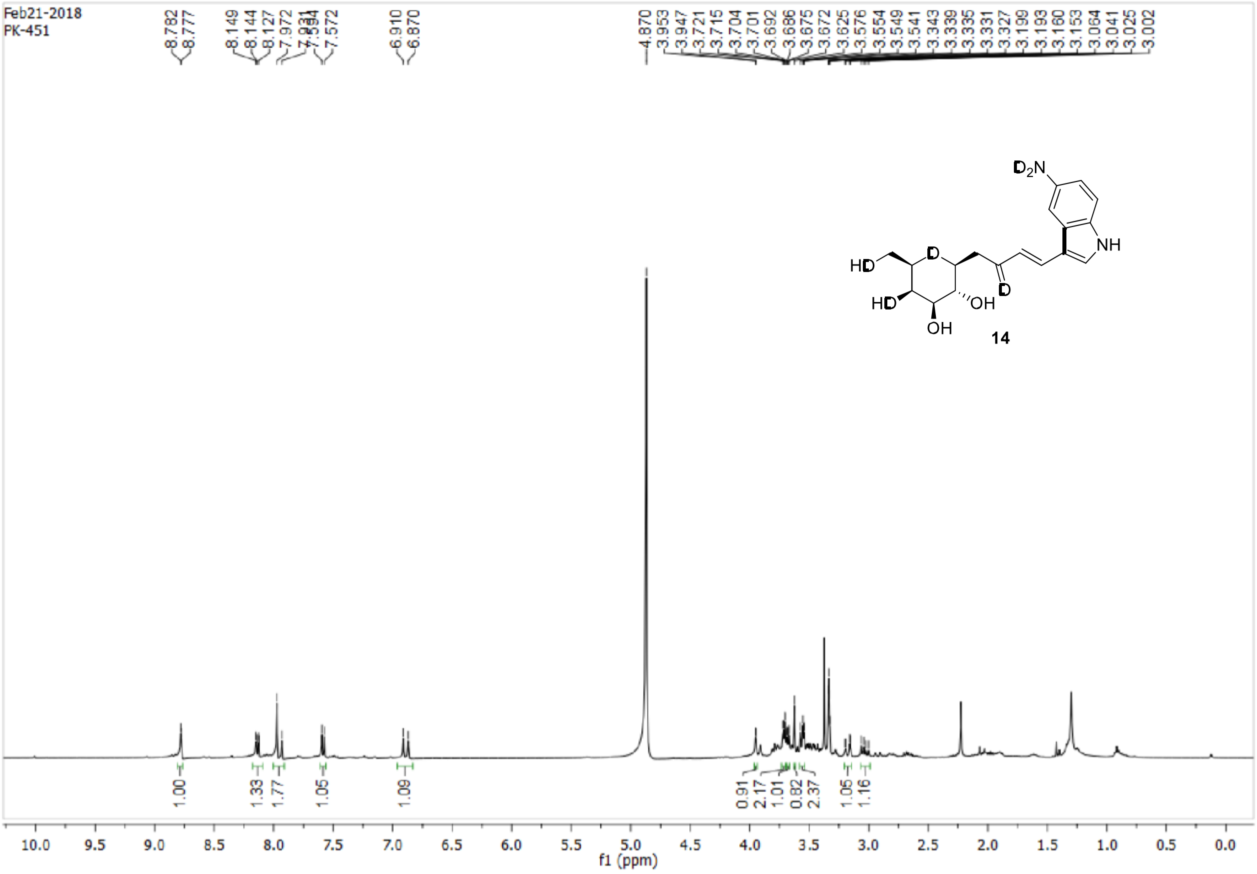
Feb21-2018
PK-451
D2N
NH
HD
HD
OH
OH
D
D
14
f1 (ppm)

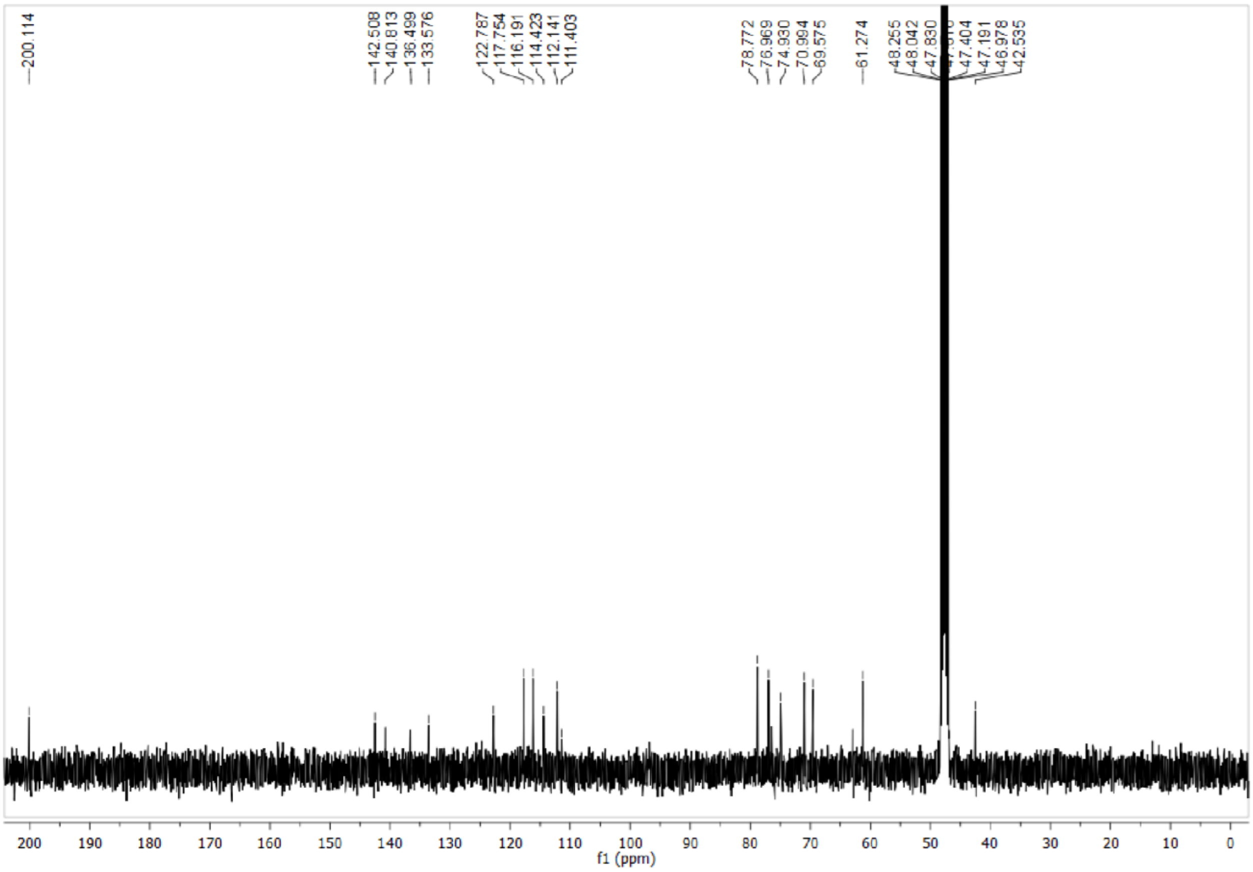
f1 (ppm)

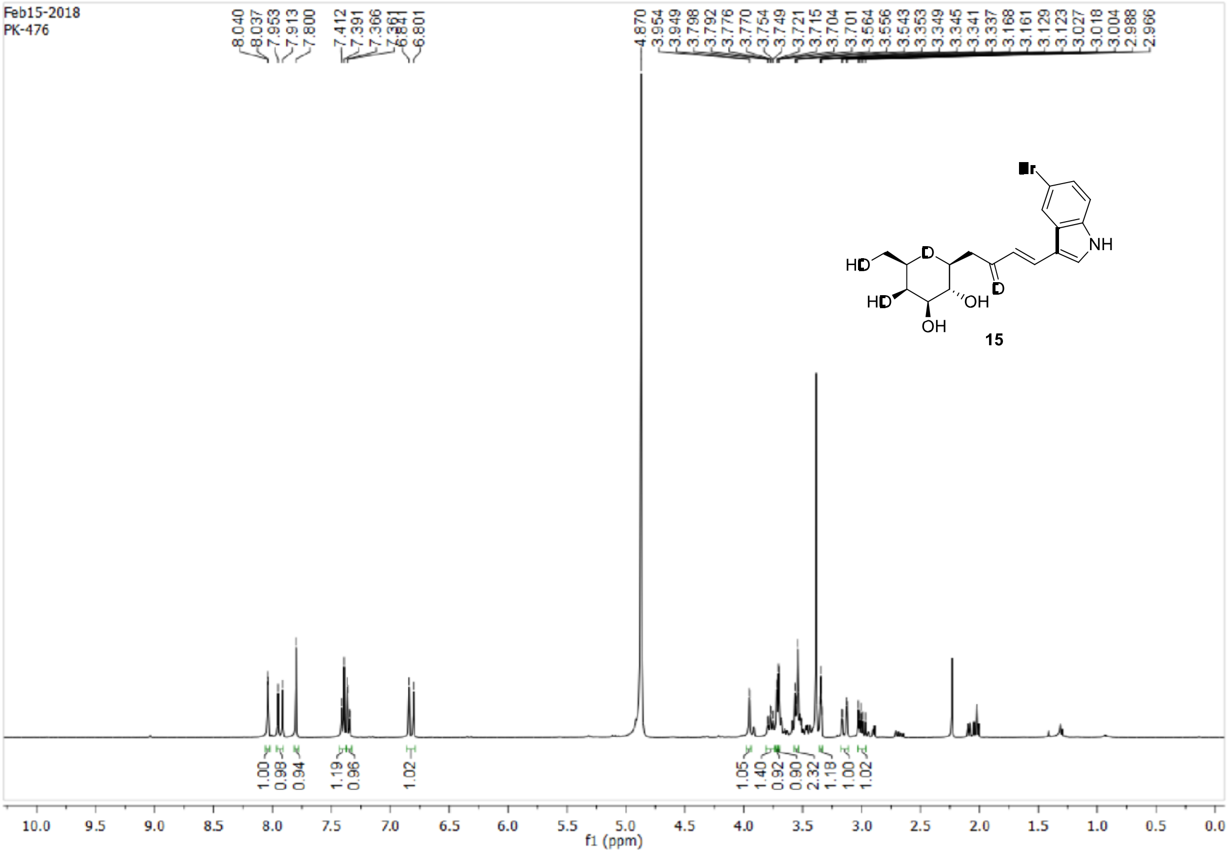

Feb15-2018
PK-476
15
f1 (ppm)

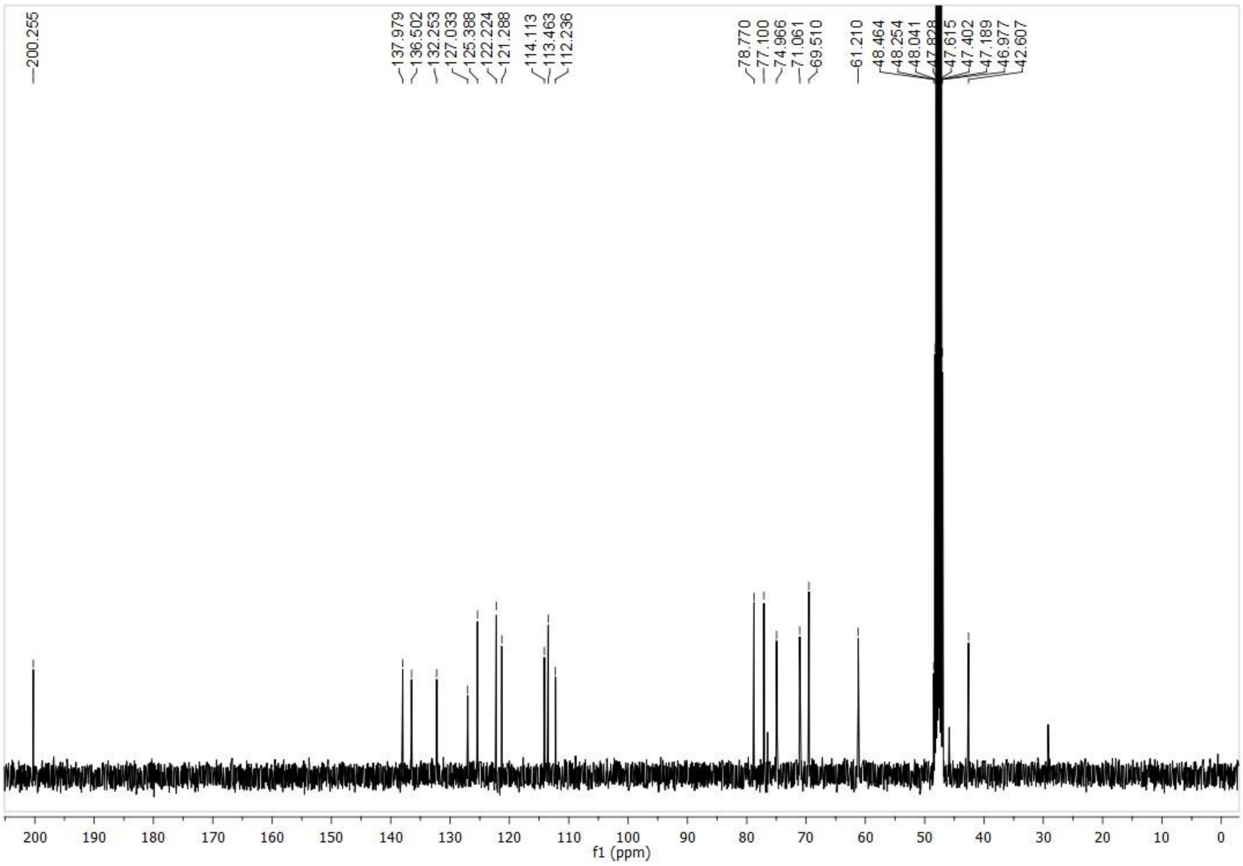

f1 (ppm)

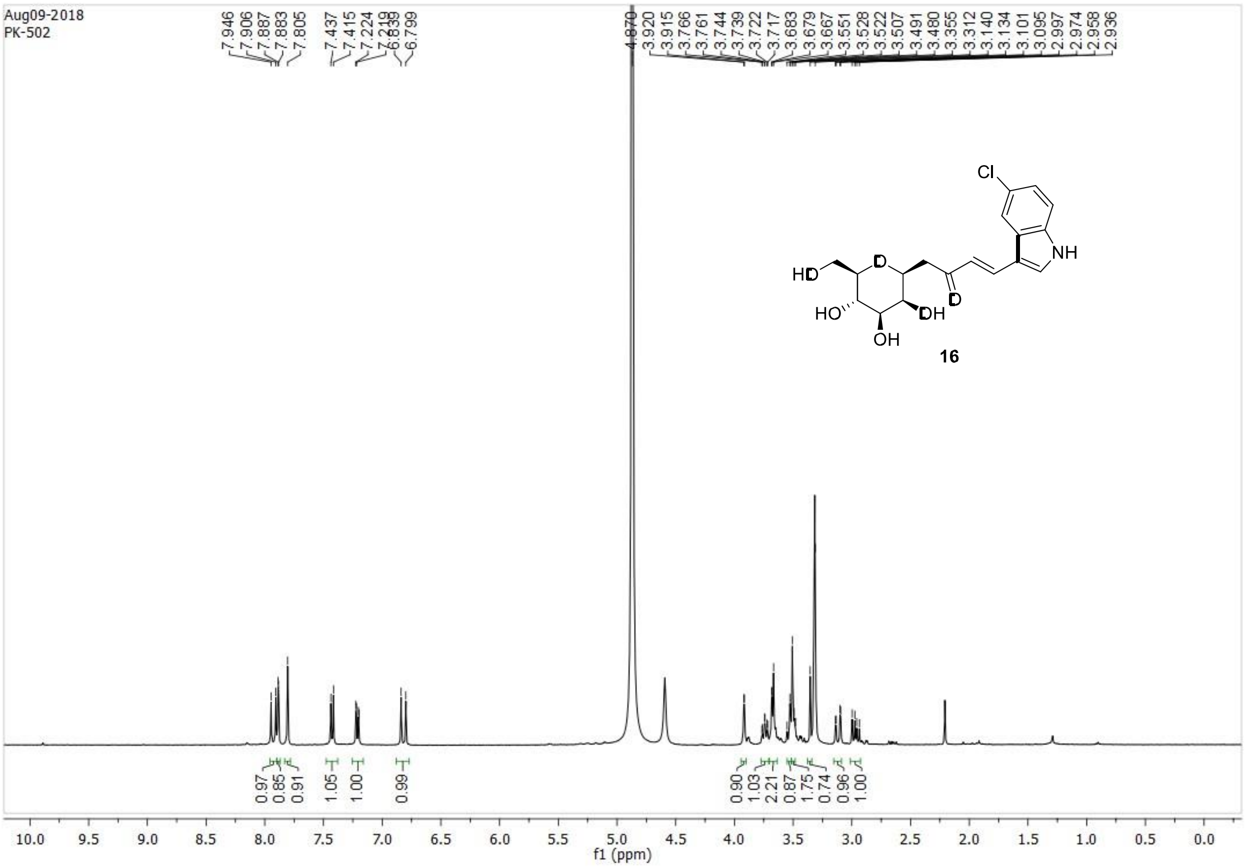
Aug09-2018
PK-502
7.946 7.906 7.887 7.883 7.805 7.437 7.415 7.224 7.219 6.839 6.799
4.870
3.920 3.915 3.766 3.761 3.744 3.739 3.722 3.717 3.683 3.679 3.667 3.551 3.528 3.522 3.507 3.491 3.480 3.355 3.312 3.140 3.134 3.101 3.095 2.997 2.974 2.958 2.936
Cl
NH
HO
O
OH
HO
OH
O
16
0.97 0.85 0.91 1.05 1.00 0.99
0.90 1.03 2.21 0.87 1.75 0.74 0.96 1.00
f1 (ppm)

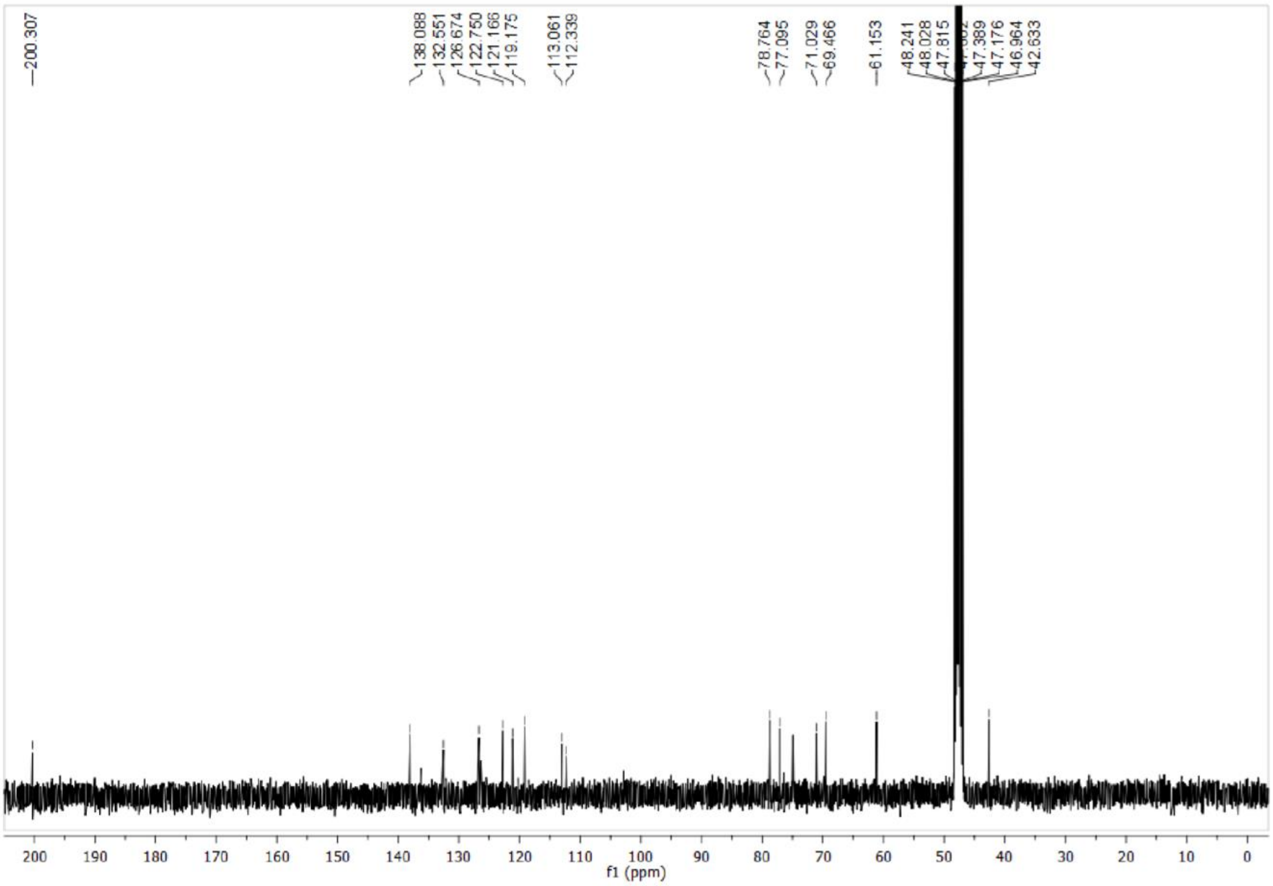
200.307
138.088 132.551 126.674 122.750 121.166 119.175
113.061 112.339
78.764 77.095 71.029 69.466
61.153
48.241 48.028 47.815 47.389 47.176 46.964 42.633
f1 (ppm)

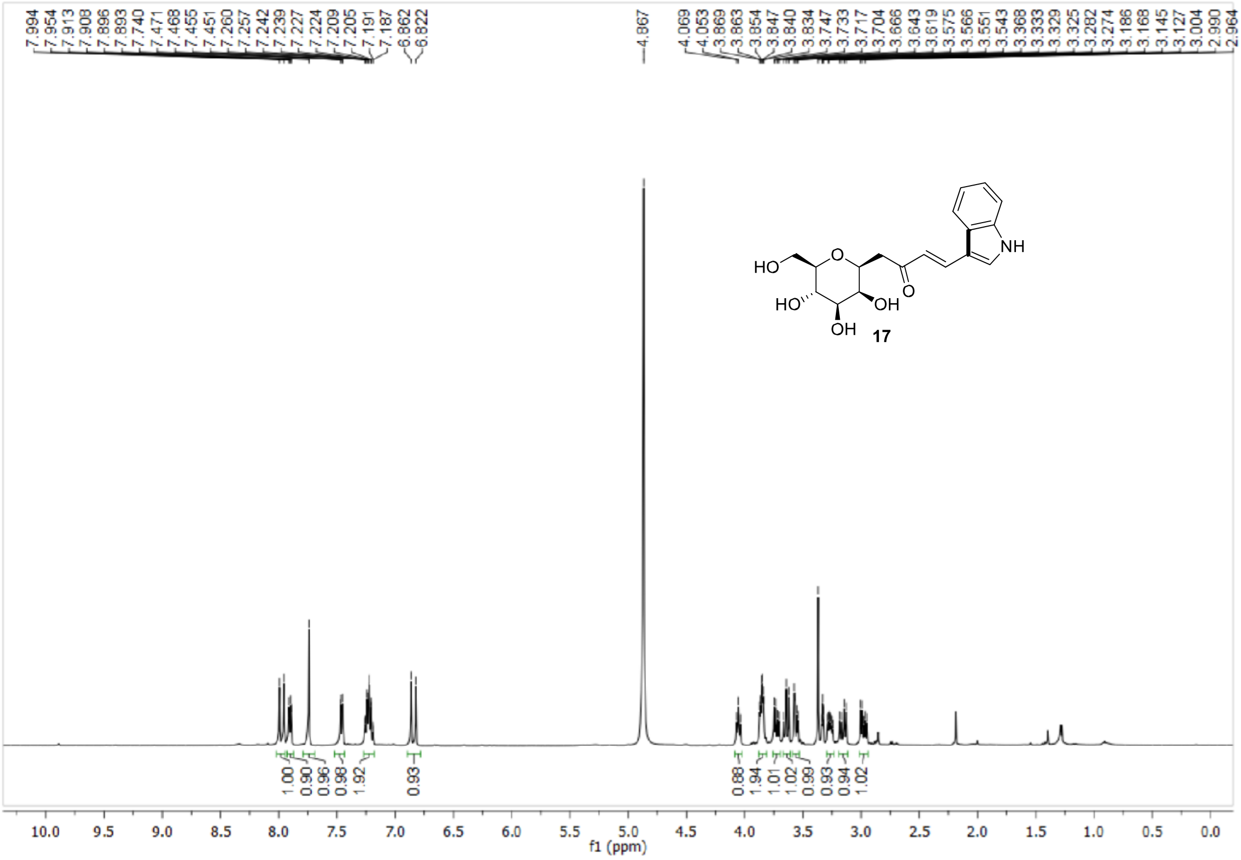
7.994 7.954 7.913 7.908 7.896 7.893 7.740 7.471 7.468 7.455 7.451 7.260 7.257 7.242 7.239 7.227 7.224 7.209 7.205 7.191 7.187 6.862 6.822
4.867
4.069 4.053 3.869 3.863 3.854 3.847 3.840 3.834 3.747 3.733 3.717 3.704 3.666 3.643 3.619 3.575 3.566 3.551 3.543 3.368 3.333 3.329 3.325 3.282 3.274 3.186 3.168 3.145 3.127 3.004 2.990 2.964
HO
HO
OH
OH
O
O
NH
17
1.00 0.90 0.96 0.98 1.92 0.93
0.88 1.94 1.01 1.02 0.99 0.93 0.94 1.02
10.0 9.5 9.0 8.5 8.0 7.5 7.0 6.5 6.0 5.5 5.0 4.5 4.0 3.5 3.0 2.5 2.0 1.5 1.0 0.5 0.0
f1 (ppm)

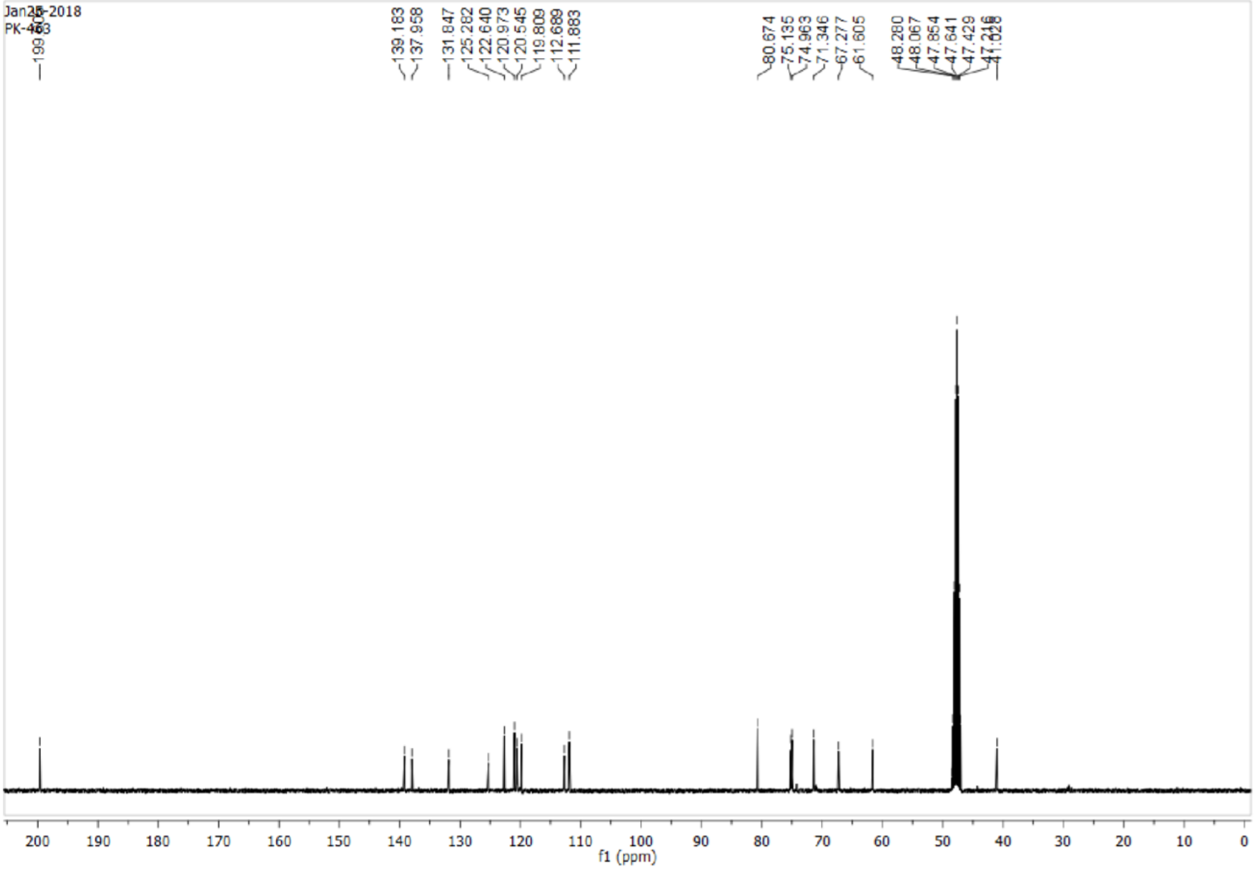
139.183 137.958 131.847 125.282 122.640 120.973 120.545 119.809 112.689 111.883
80.674 75.135 74.963 71.346 67.277 61.605
48.280 48.067 47.854 47.641 47.429
200 190 180 170 160 150 140 130 120 110 100 90 80 70 60 50 40 30 20 10 0
f1 (ppm)

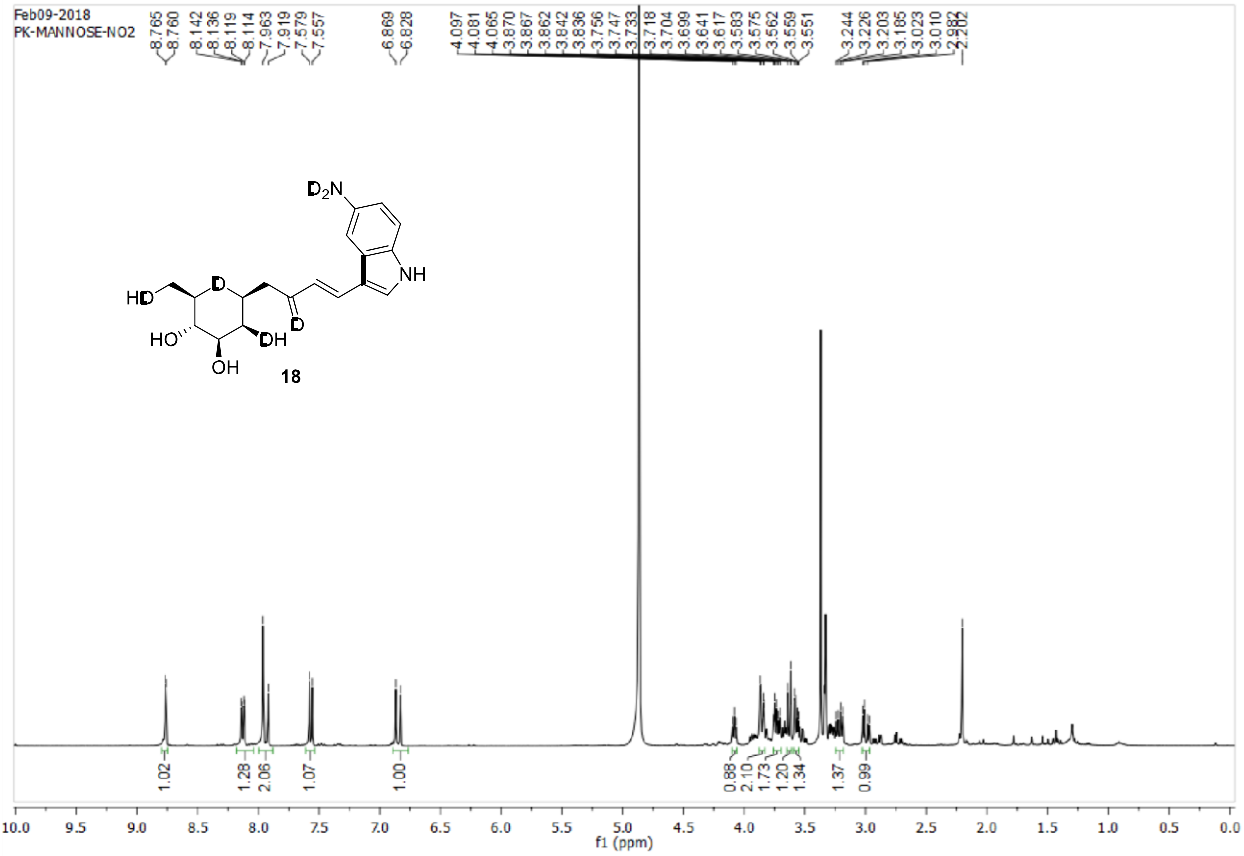
Feb09-2018
PK-MANNOSE-NO2
8.765
8.760
8.142
8.136
8.119
8.114
7.963
7.919
7.579
7.557
6.869
6.828
4.097
4.081
4.065
3.870
3.867
3.862
3.842
3.836
3.756
3.747
3.733
3.718
3.704
3.699
3.641
3.617
3.583
3.575
3.562
3.559
3.551
3.244
3.226
3.203
3.185
3.023
3.010
1.02
1.28
2.06
1.07
1.00
0.88
2.10
1.73
1.20
1.34
1.37
0.99
D2N
NH
HO
HO
OH
OH
18
f1 (ppm)

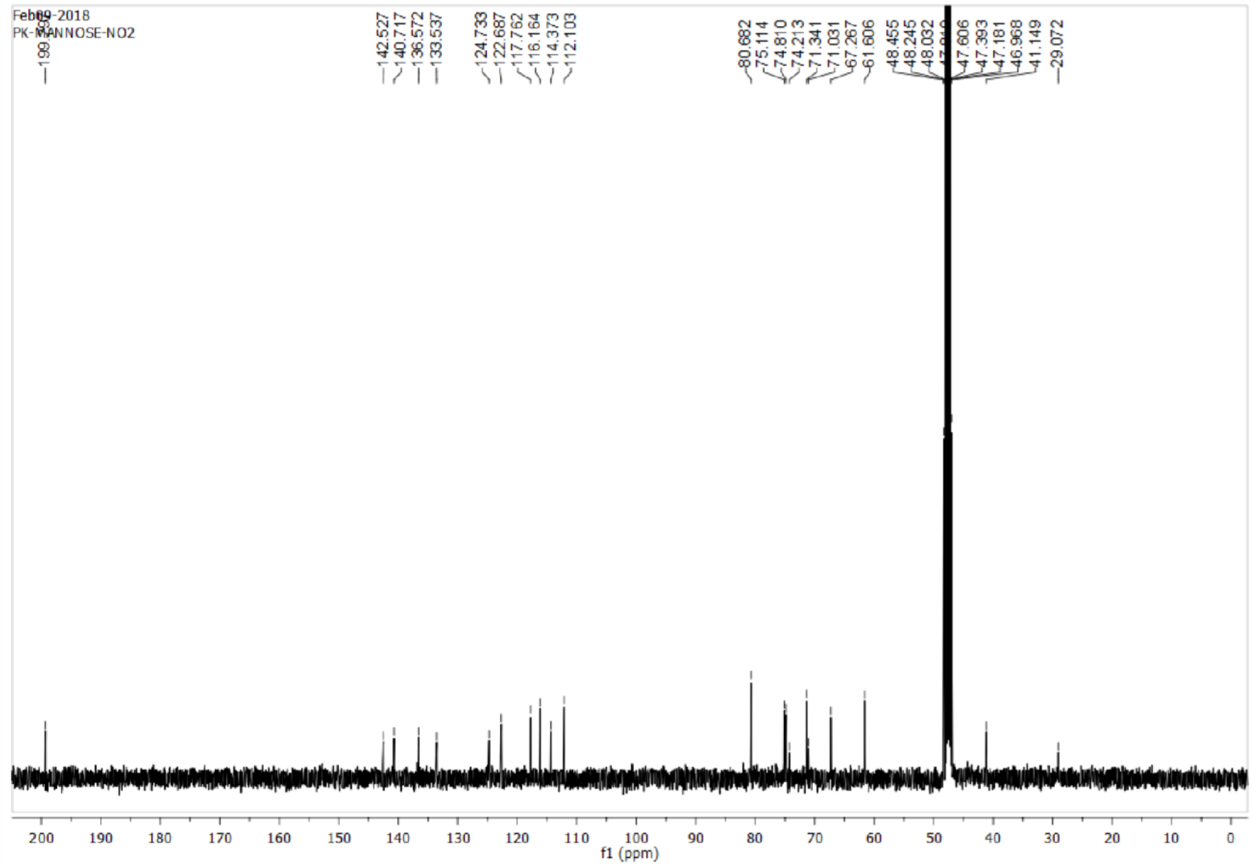
Feb09-2018
PK-MANNOSE-NO2
142.527
140.717
136.572
133.537
124.733
122.687
117.762
116.164
114.373
112.103
80.682
75.114
74.810
74.213
71.341
71.031
67.267
61.606
48.455
48.245
48.032
47.606
47.393
47.181
46.968
41.149
29.072
f1 (ppm)

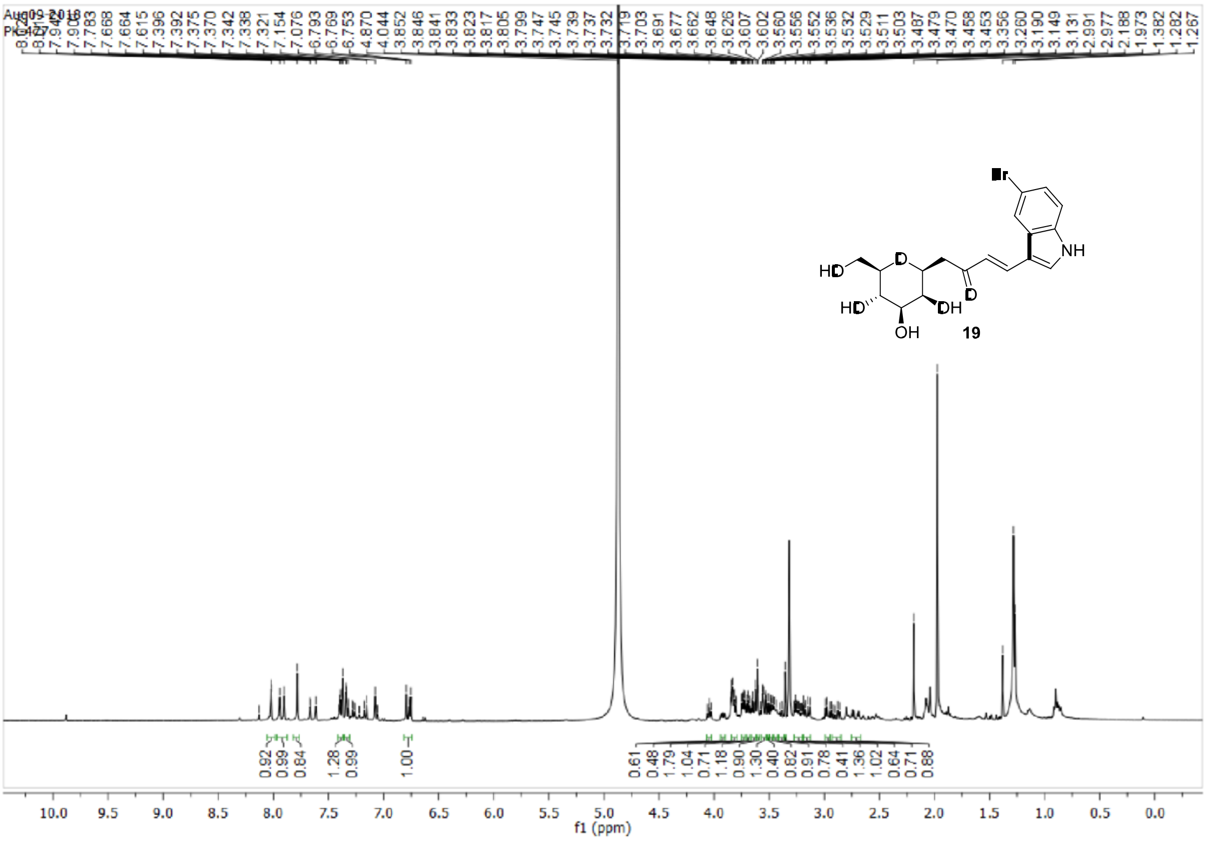
Aug09-2016
7.906 7.783 7.668 7.664 7.615 7.396 7.392 7.375 7.370 7.342 7.338 7.321 7.154 7.076 6.793 6.769 6.753 4.870 4.044 3.852 3.846 3.841 3.833 3.823 3.817 3.805 3.799 3.747 3.745 3.739 3.737 3.732 3.719 3.703 3.691 3.677 3.662 3.648 3.626 3.607 3.602 3.560 3.556 3.552 3.536 3.532 3.529 3.511 3.503 3.487 3.479 3.470 3.458 3.453 3.356 3.260 3.190 3.149 3.131 2.991 2.977 2.188 1.973 1.382 1.282 1.267
Br
NH
HO
O
HO
OH
OH
19
0.92 0.99 0.84 1.28 0.99 1.00
0.61 0.48 1.79 1.04 0.71 1.18 0.90 1.30 0.40 0.82 0.91 0.78 0.41 1.36 1.02 0.64 0.71 0.88
10.0 9.5 9.0 8.5 8.0 7.5 7.0 6.5 6.0 5.5 5.0 4.5 4.0 3.5 3.0 2.5 2.0 1.5 1.0 0.5 0.0
f1 (ppm)

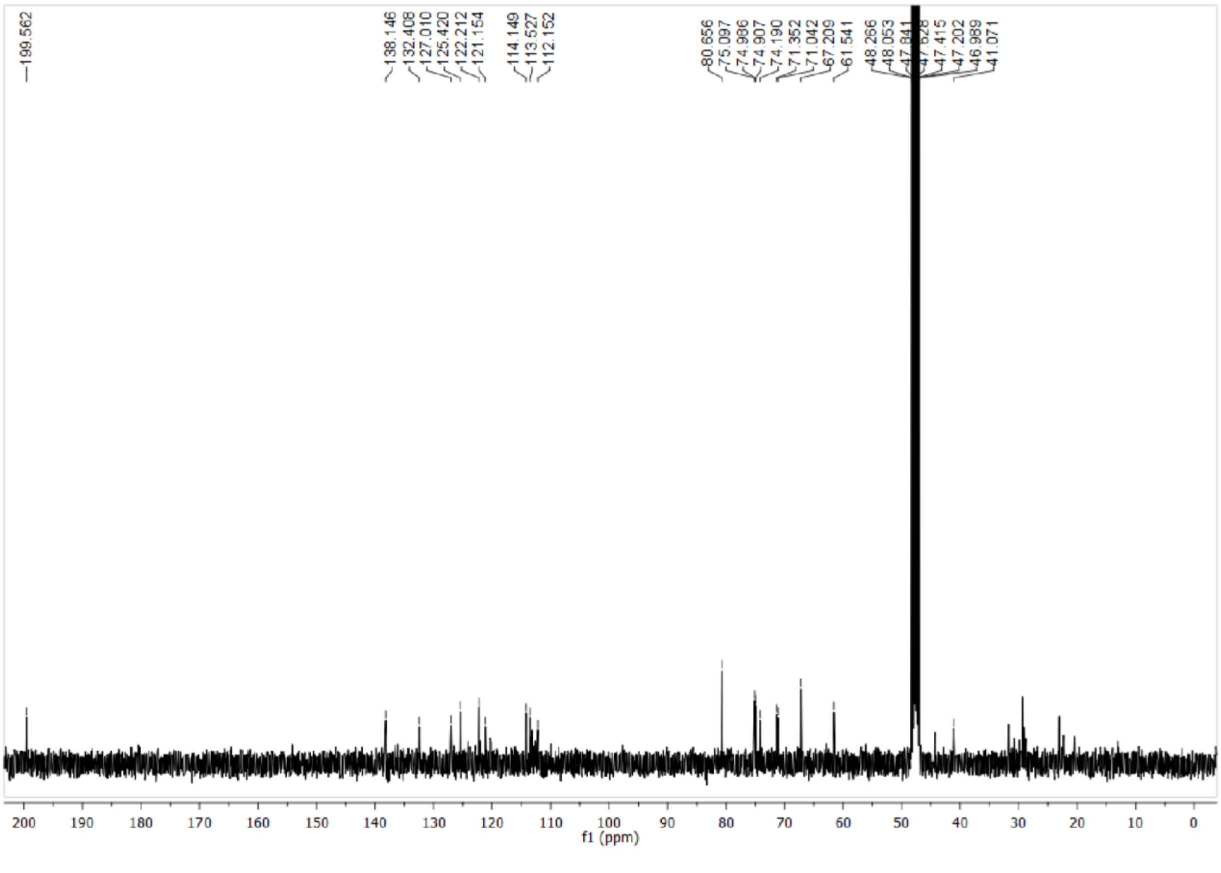
199.562
138.146 132.408 127.010 125.420 122.212 121.154 114.149 113.527 112.152
80.656 75.097 74.986 74.907 74.190 71.352 71.042 67.209 61.541 48.266 48.053 47.841 47.628 47.415 47.202 46.989 41.071
200 190 180 170 160 150 140 130 120 110 100 90 80 70 60 50 40 30 20 10 0
f1 (ppm)

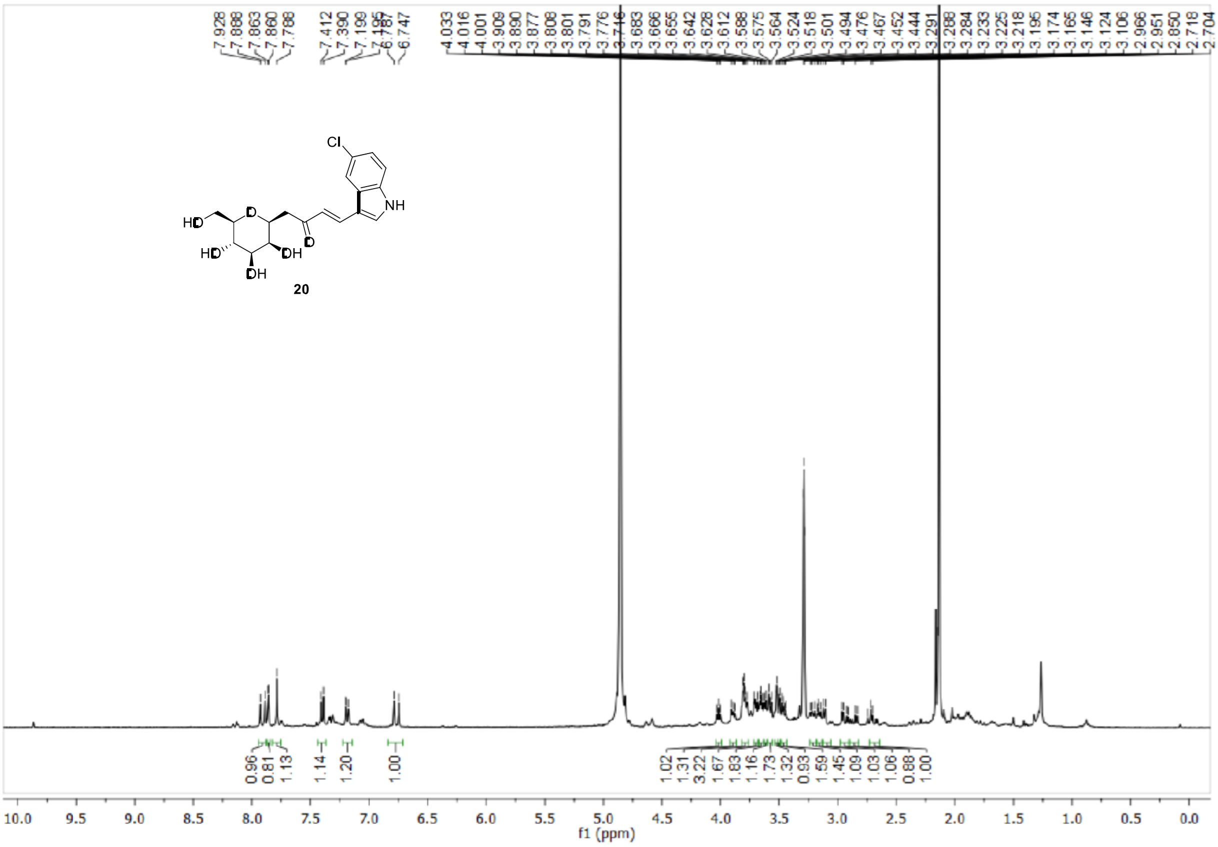
Cl
NH
HO
HO
OH
OH
O
O
20
f1 (ppm)

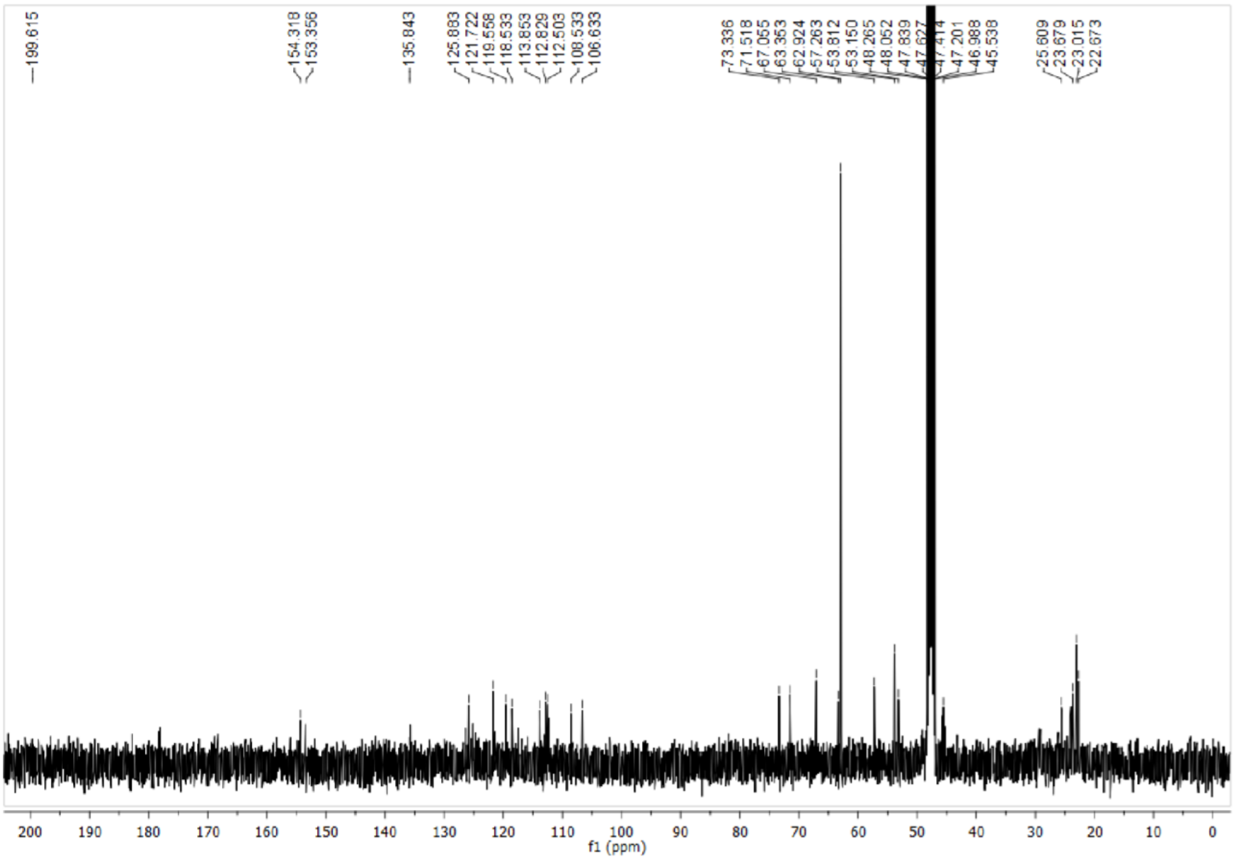
f1 (ppm)

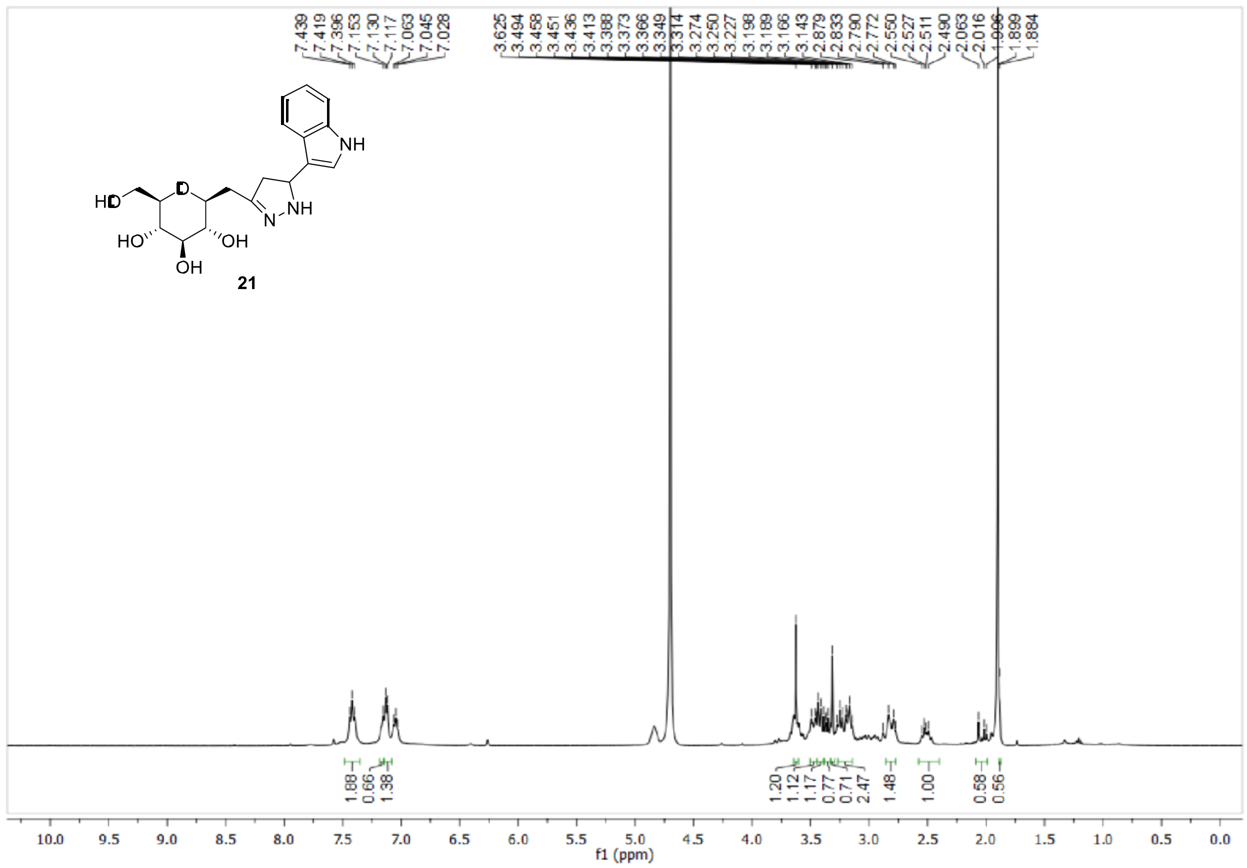
21
f1 (ppm)

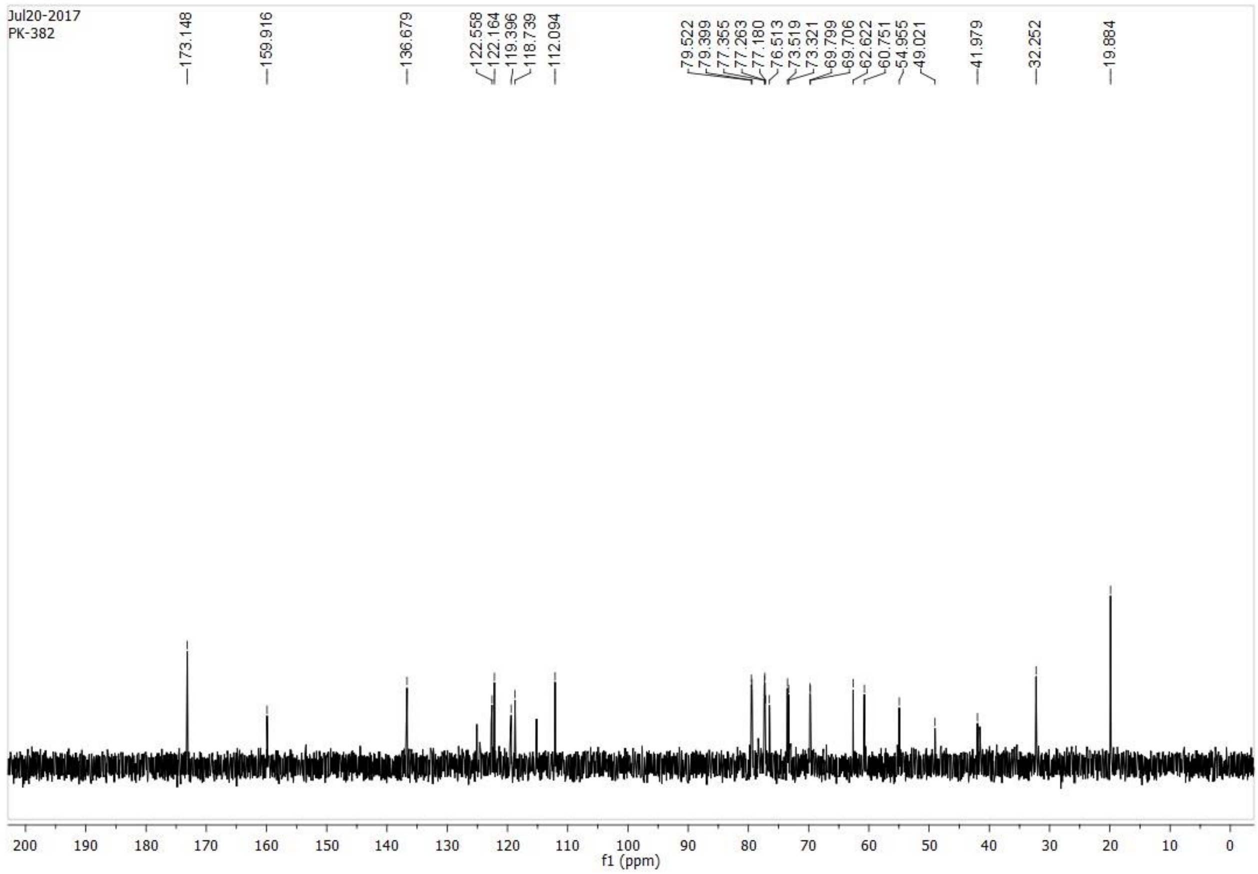
Jul20-2017
PK-382
f1 (ppm)

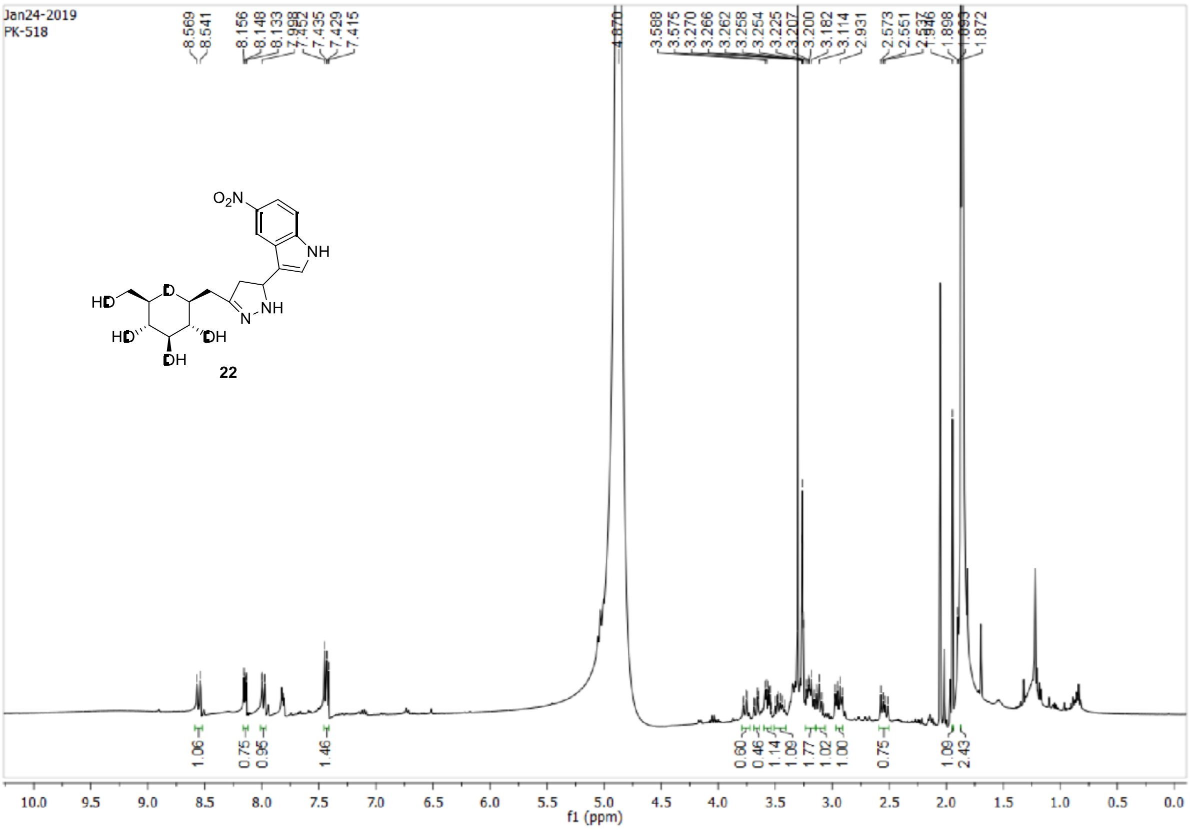
Jan24-2019
PK-518
O2N
NH
HO
OH
NH
N
22
f1 (ppm)

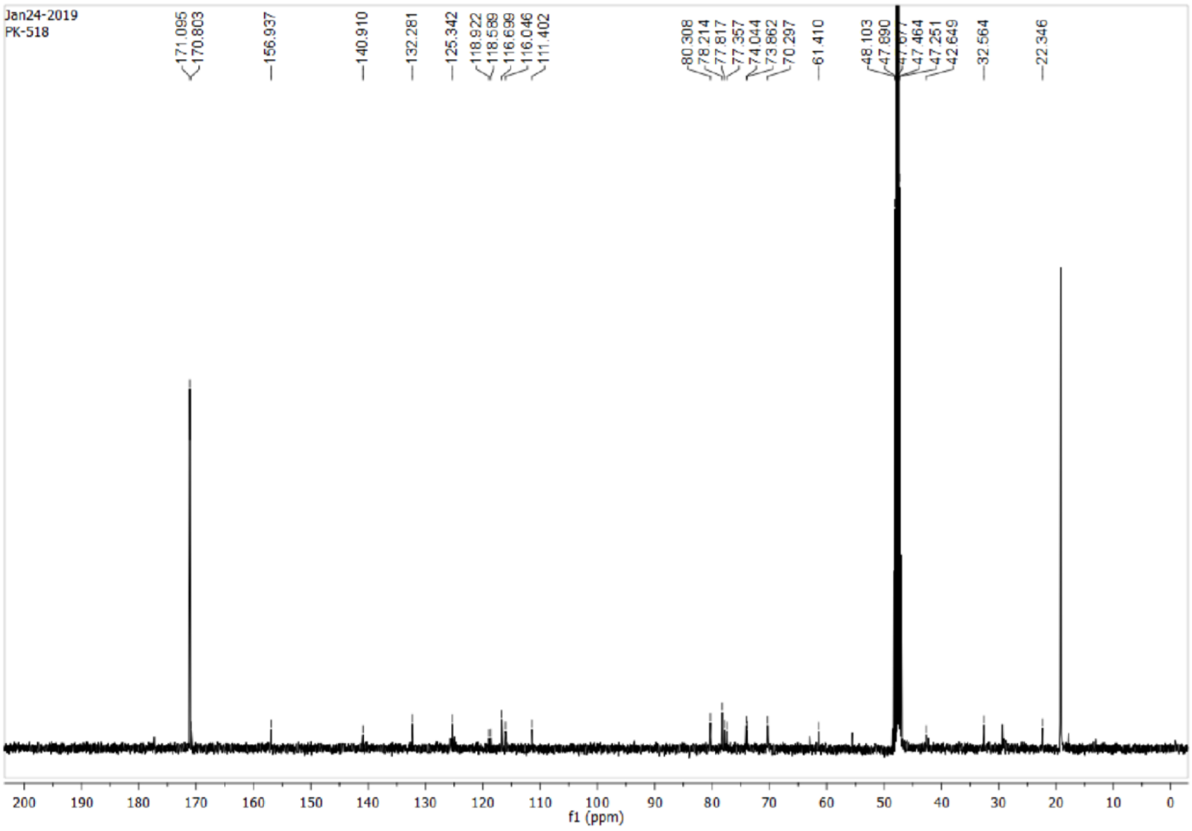
Jan24-2019
PK-518
f1 (ppm)

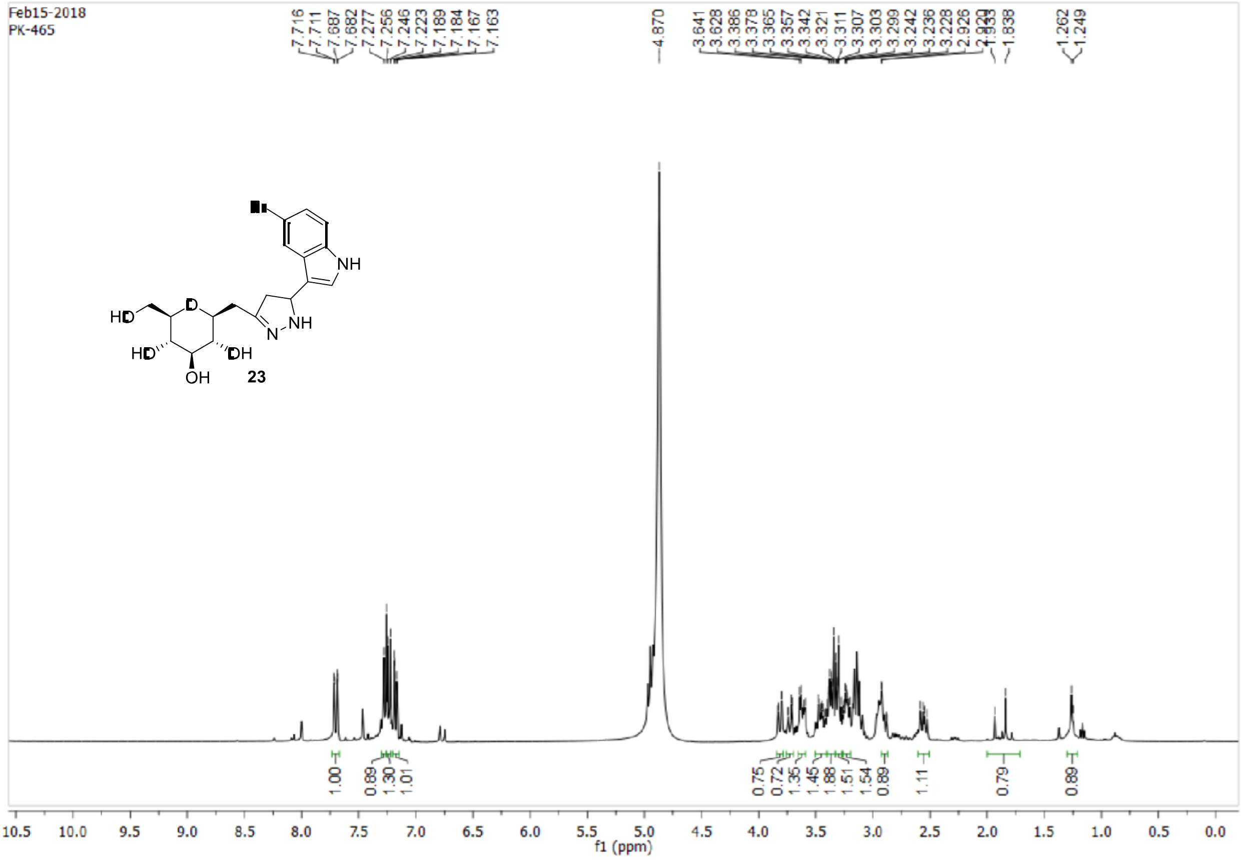
Feb15-2018
PK-465
7.716
7.711
7.687
7.682
7.277
7.256
7.246
7.223
7.189
7.184
7.167
7.163
4.870
3.641
3.628
3.386
3.378
3.365
3.357
3.342
3.321
3.311
3.307
3.303
3.299
3.242
3.236
3.228
2.926
1.838
1.262
1.249
NH
NH
N
HO
HO
OH
OH
O
23
1.00
0.89
1.30
1.01
0.75
0.72
1.35
1.45
1.88
1.51
1.54
0.89
1.11
0.79
0.89
f1 (ppm)

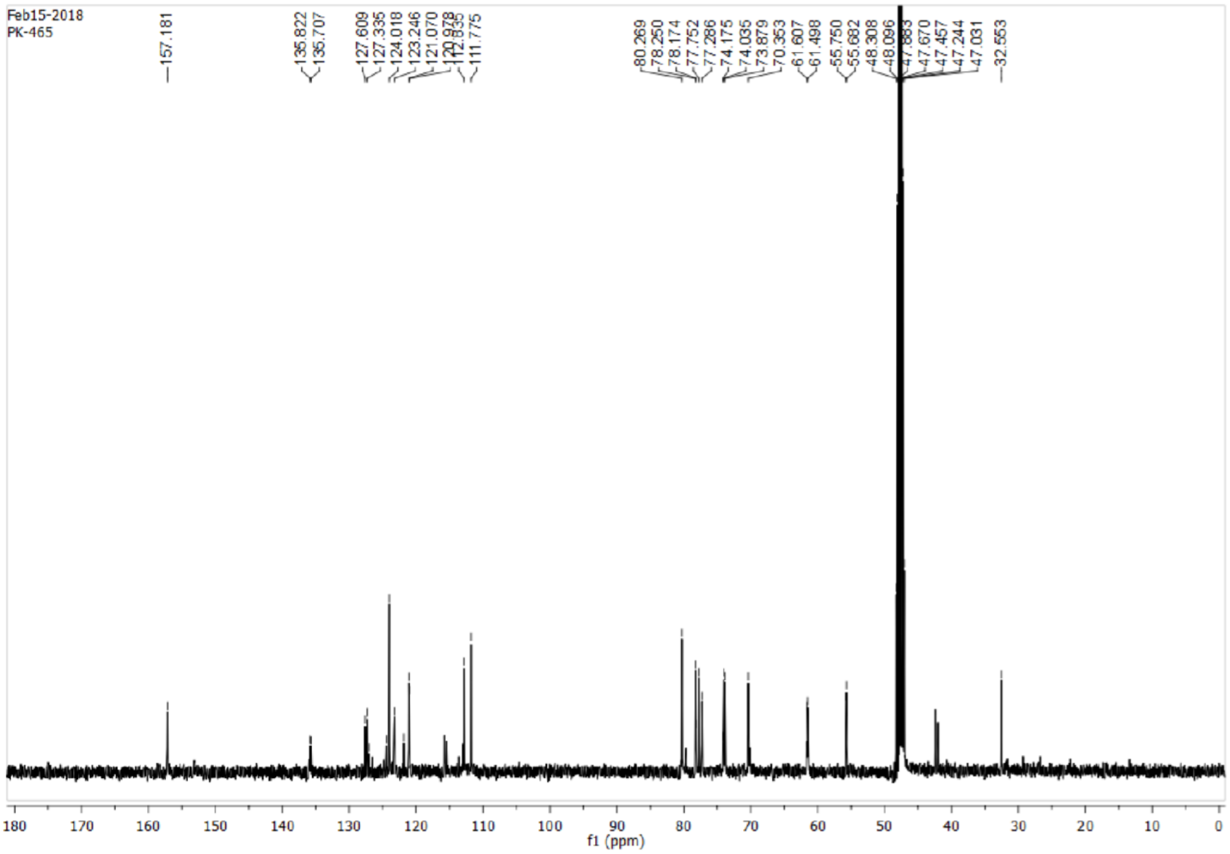
Feb15-2018
PK-465
157.181
135.822
135.707
127.609
127.335
124.018
123.246
121.070
111.775
80.269
78.250
78.174
77.752
77.286
74.175
74.035
73.879
70.353
61.607
61.498
55.750
55.682
48.308
48.096
47.883
47.670
47.457
47.244
47.031
32.553
f1 (ppm)

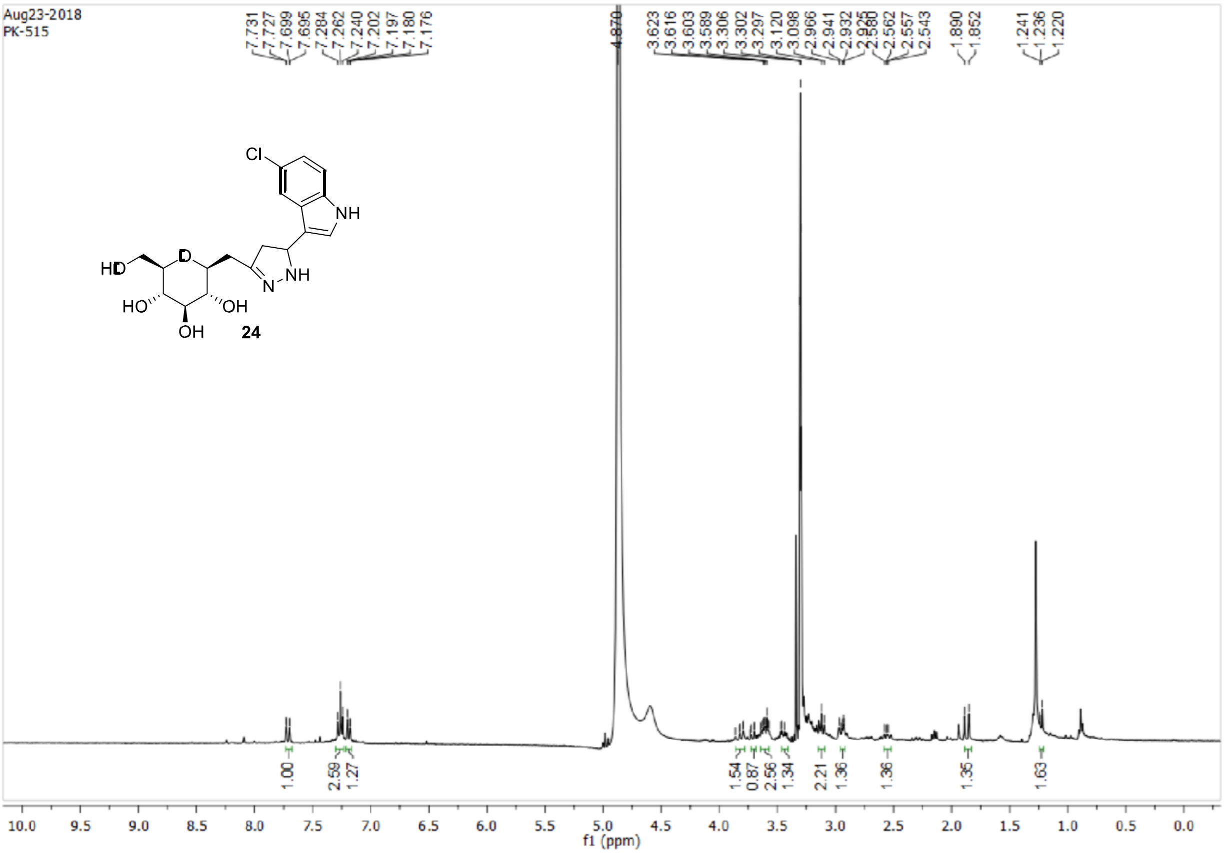
Aug23-2018
PK-515
7.731 7.727 7.699 7.695 7.284 7.262 7.240 7.202 7.197 7.180 7.176
4.870
3.623 3.616 3.603 3.589 3.306 3.302 3.297 3.120 3.098 2.966 2.941 2.932 2.925 2.580 2.562 2.557 2.543 1.890 1.852 1.241 1.236 1.220
Cl
NH
HO
O
N
NH
HO
OH
OH
24
1.00 2.59 1.27 1.54 0.87 2.56 1.34 2.21 1.36 1.36 1.35 1.63
10.0 9.5 9.0 8.5 8.0 7.5 7.0 6.5 6.0 5.5 5.0 4.5 4.0 3.5 3.0 2.5 2.0 1.5 1.0 0.5 0.0
f1 (ppm)

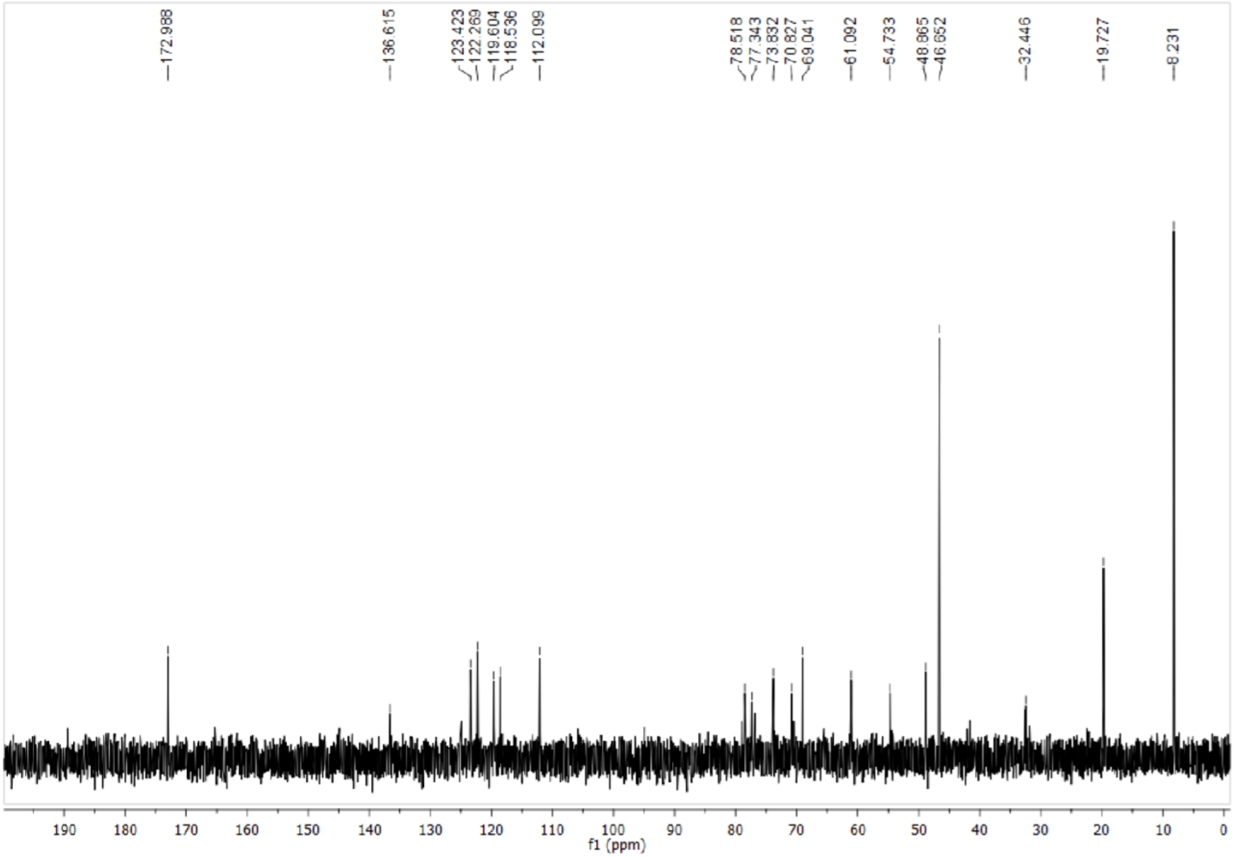
172.988
136.615
123.423 122.269 119.604 118.536 112.099
78.518 77.343 73.832 70.827 69.041
61.092
54.733
48.865 46.652
32.446
19.727
8.231
190 180 170 160 150 140 130 120 110 100 90 80 70 60 50 40 30 20 10 0
f1 (ppm)

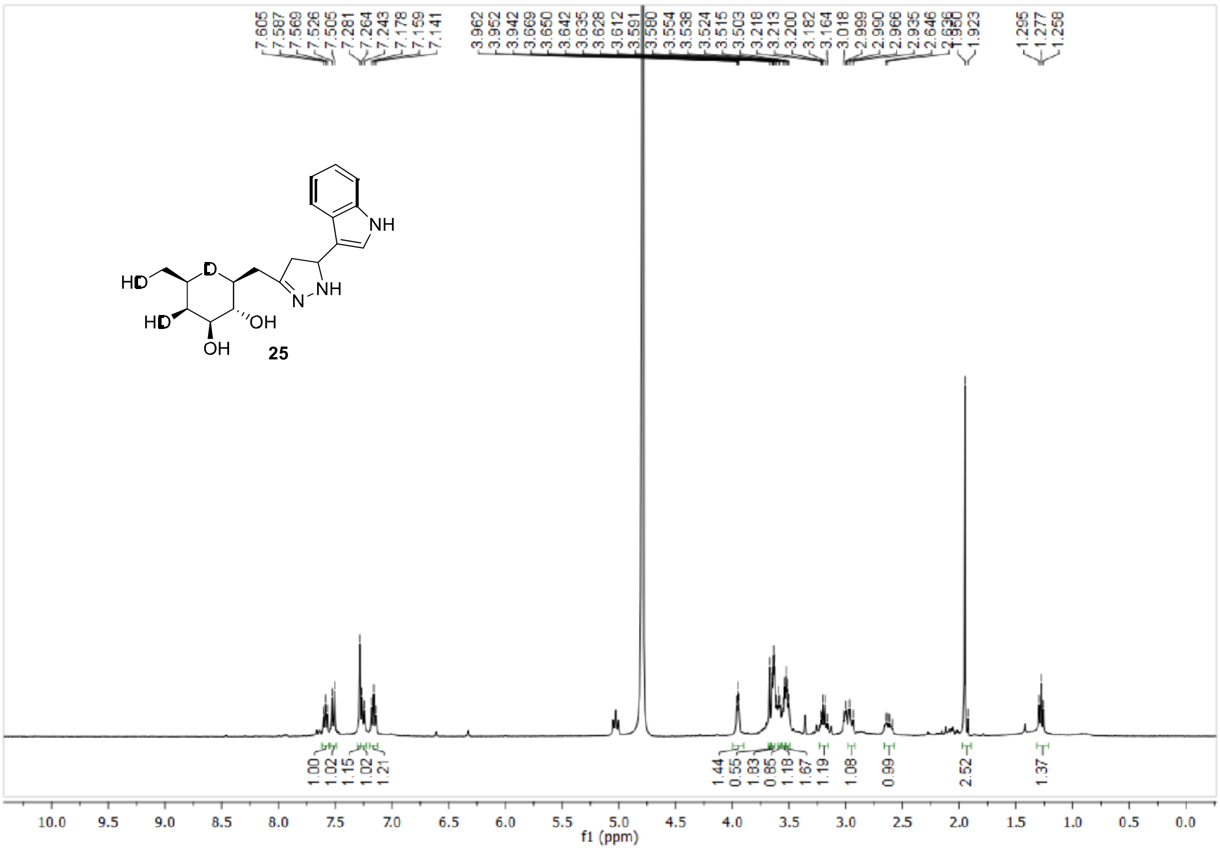

7.605
7.587
7.569
7.526
7.505
7.281
7.264
7.243
7.178
7.159
7.141
3.962
3.952
3.942
3.669
3.650
3.642
3.635
3.628
3.612
3.591
3.580
3.554
3.538
3.524
3.515
3.503
3.218
3.213
3.200
3.182
3.164
3.018
2.999
2.990
2.966
2.935
2.646
1.923
1.295
1.277
1.258
HO
O
NH
NH
N
HO
OH
OH
25
1.00
1.02
1.15
1.02
1.21
1.44
0.55
1.83
0.85
1.18
1.67
1.19
1.08
0.99
2.52
1.37
10.0
9.5
9.0
8.5
8.0
7.5
7.0
6.5
6.0
5.5
5.0
4.5
4.0
3.5
3.0
2.5
2.0
1.5
1.0
0.5
0.0
f1 (ppm)

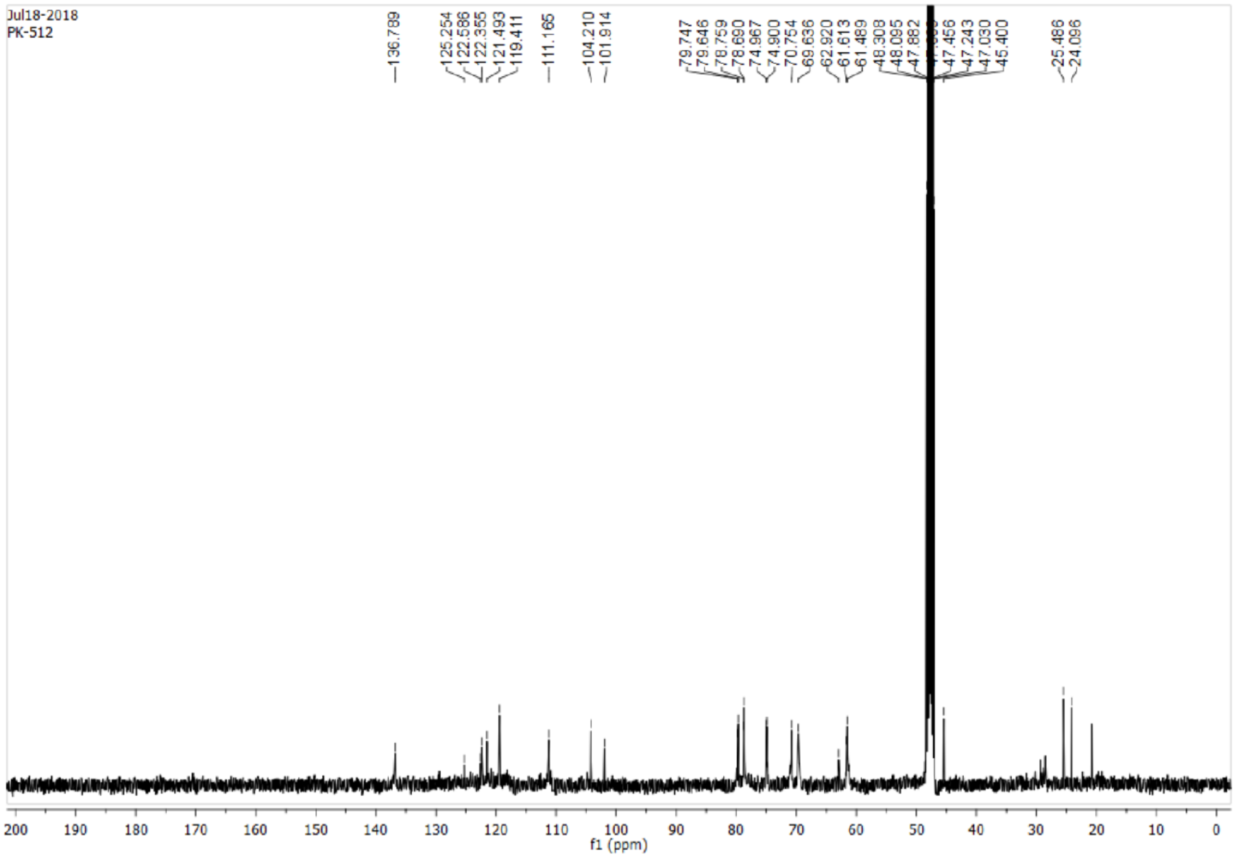

Jul18-2018
PK-512
136.789
125.254
122.586
122.355
121.493
119.411
111.165
104.210
101.914
79.747
79.646
78.759
78.690
74.967
74.900
70.754
69.636
62.920
61.613
61.489
48.308
48.095
47.882
47.456
47.243
47.030
45.400
25.486
24.096
200
190
180
170
160
150
140
130
120
110
100
90
80
70
60
50
40
30
20
10
0
f1 (ppm)

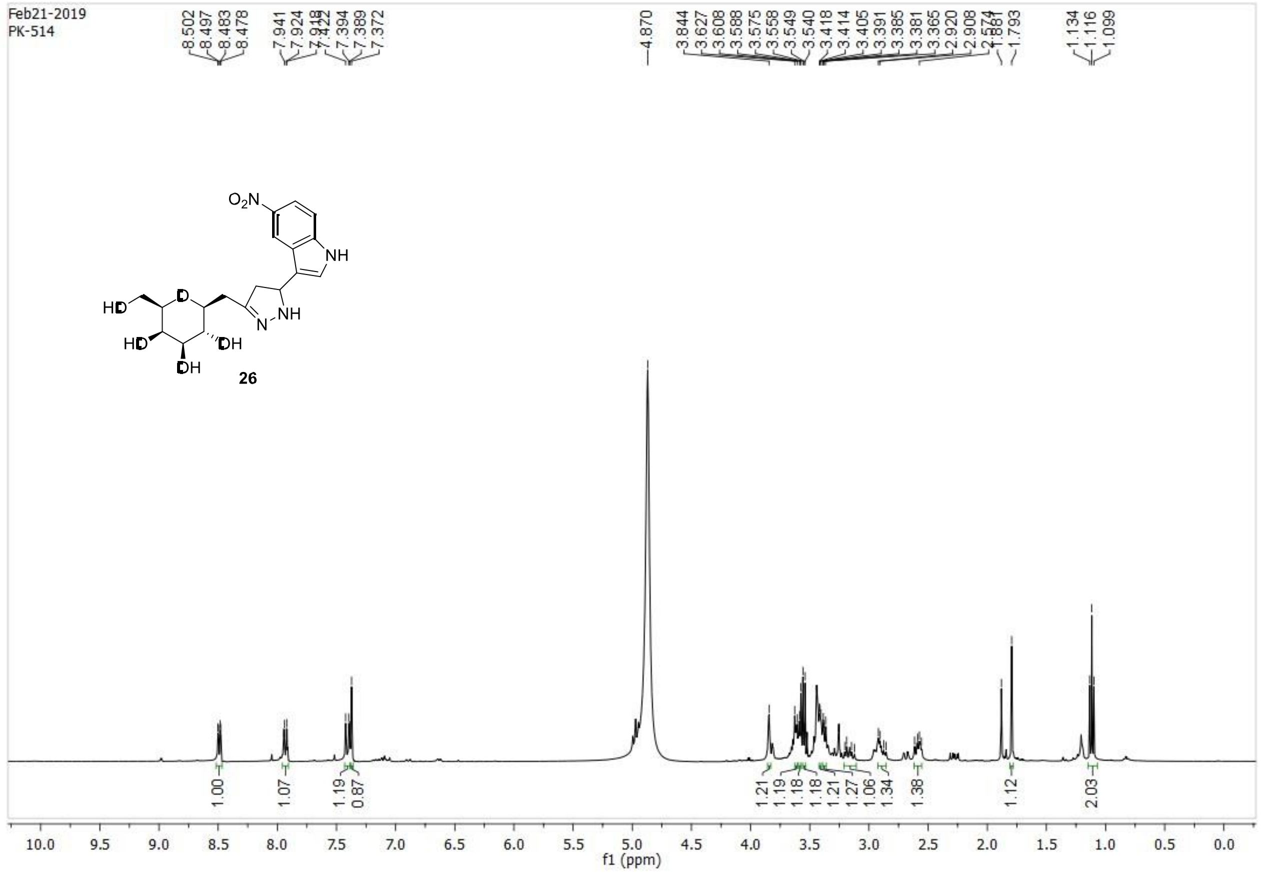
Feb21-2019
PK-514
26
f1 (ppm)

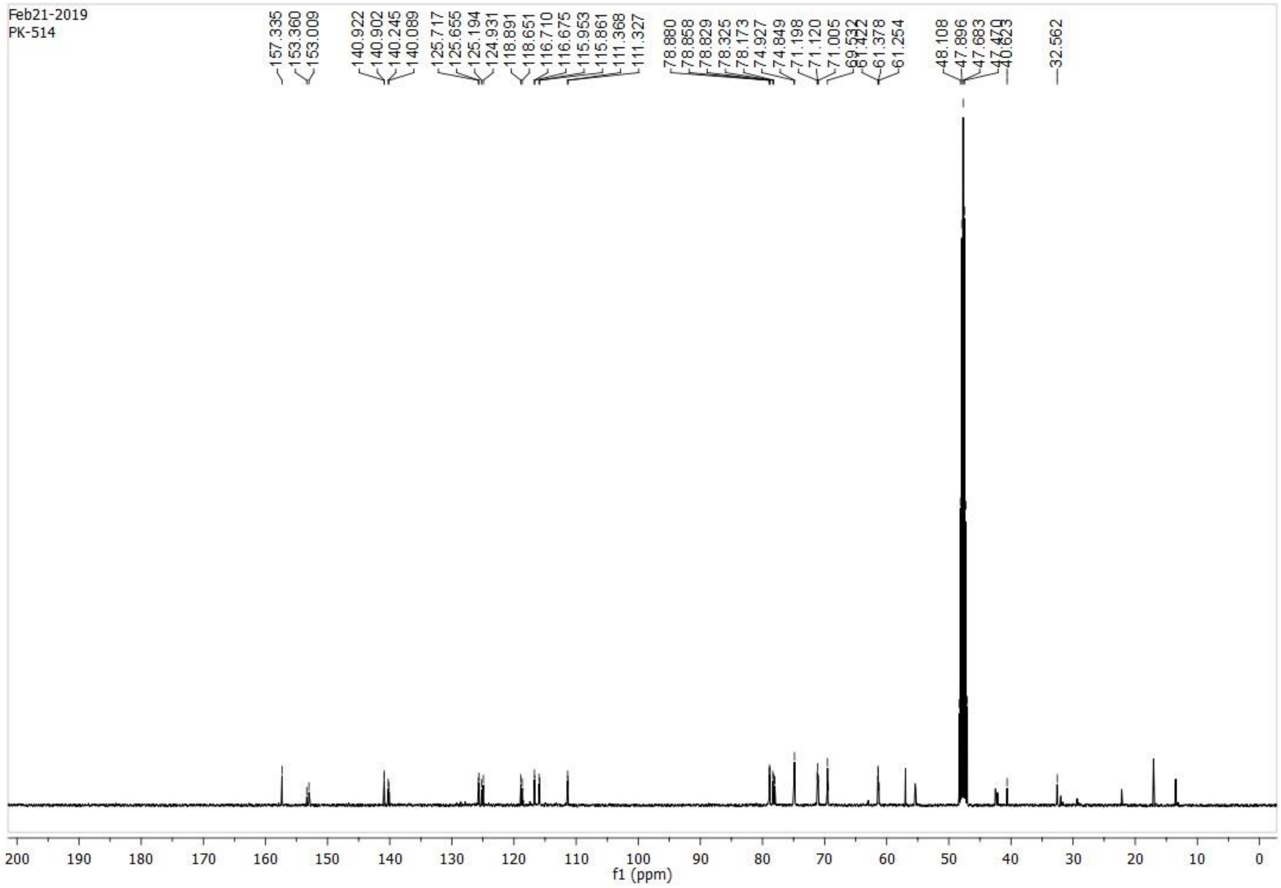
Feb21-2019
PK-514
f1 (ppm)

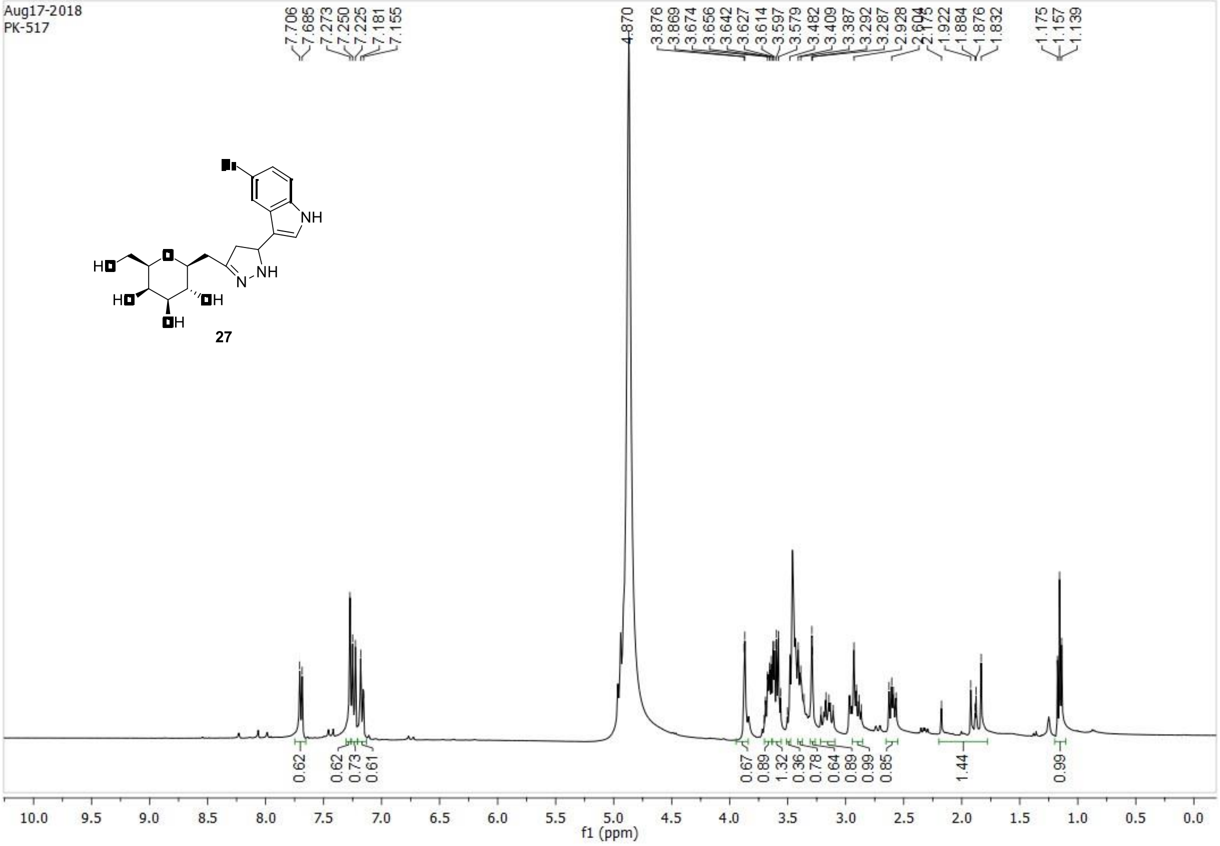
Aug17-2018
PK-517
7.706
7.685
7.273
7.250
7.225
7.181
7.155
4.870
3.876
3.869
3.674
3.656
3.642
3.627
3.614
3.597
3.579
3.482
3.409
3.387
3.292
3.287
2.928
1.922
1.884
1.876
1.832
1.175
1.157
1.139
NH
HO
N
NH
OH
27
0.62
0.62
0.73
0.61
0.67
0.89
1.32
0.36
0.78
0.64
0.89
0.99
0.85
1.44
0.99
f1 (ppm)

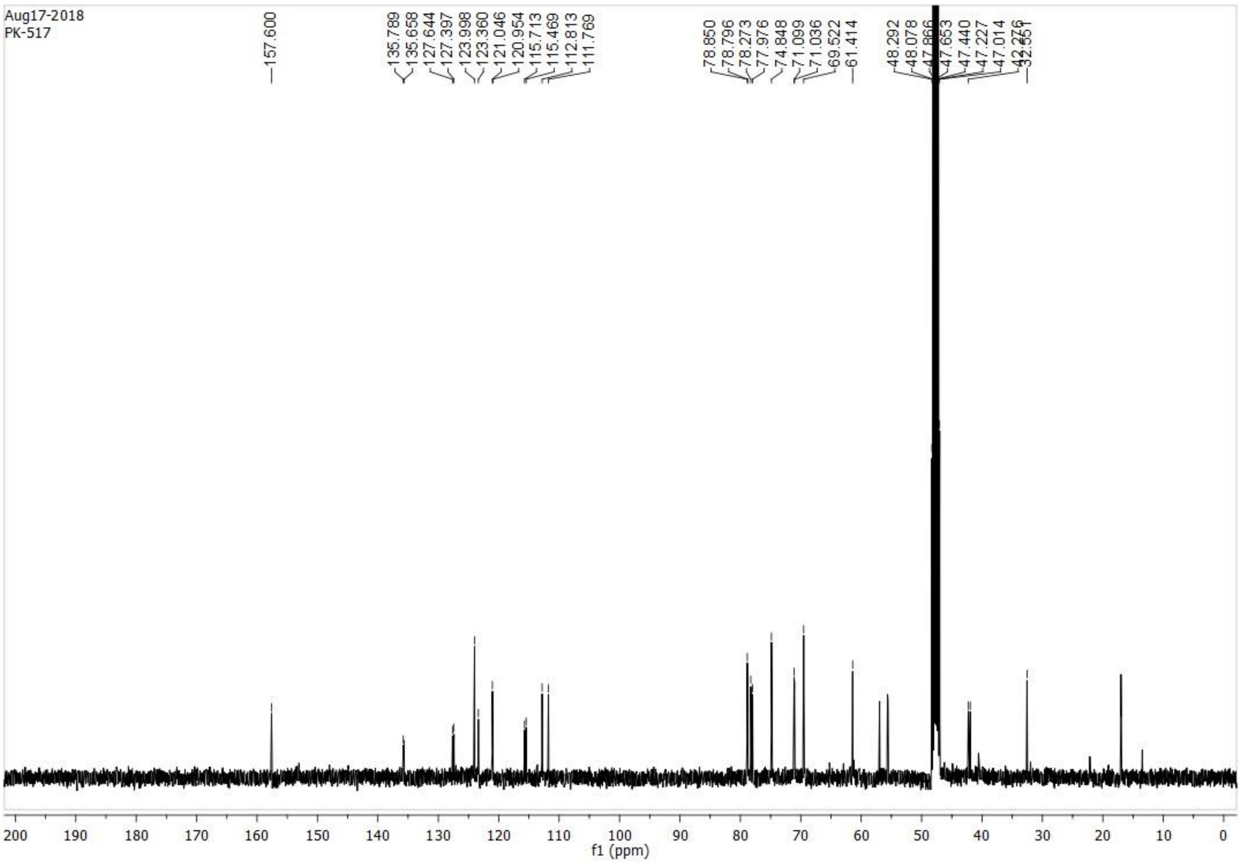
Aug17-2018
PK-517
157.600
135.789
135.658
127.644
127.397
123.998
123.360
121.046
120.954
115.713
115.469
112.813
111.769
78.850
78.796
78.273
77.976
74.848
71.099
71.036
69.522
61.414
48.292
48.078
47.653
47.440
47.227
47.014
f1 (ppm)

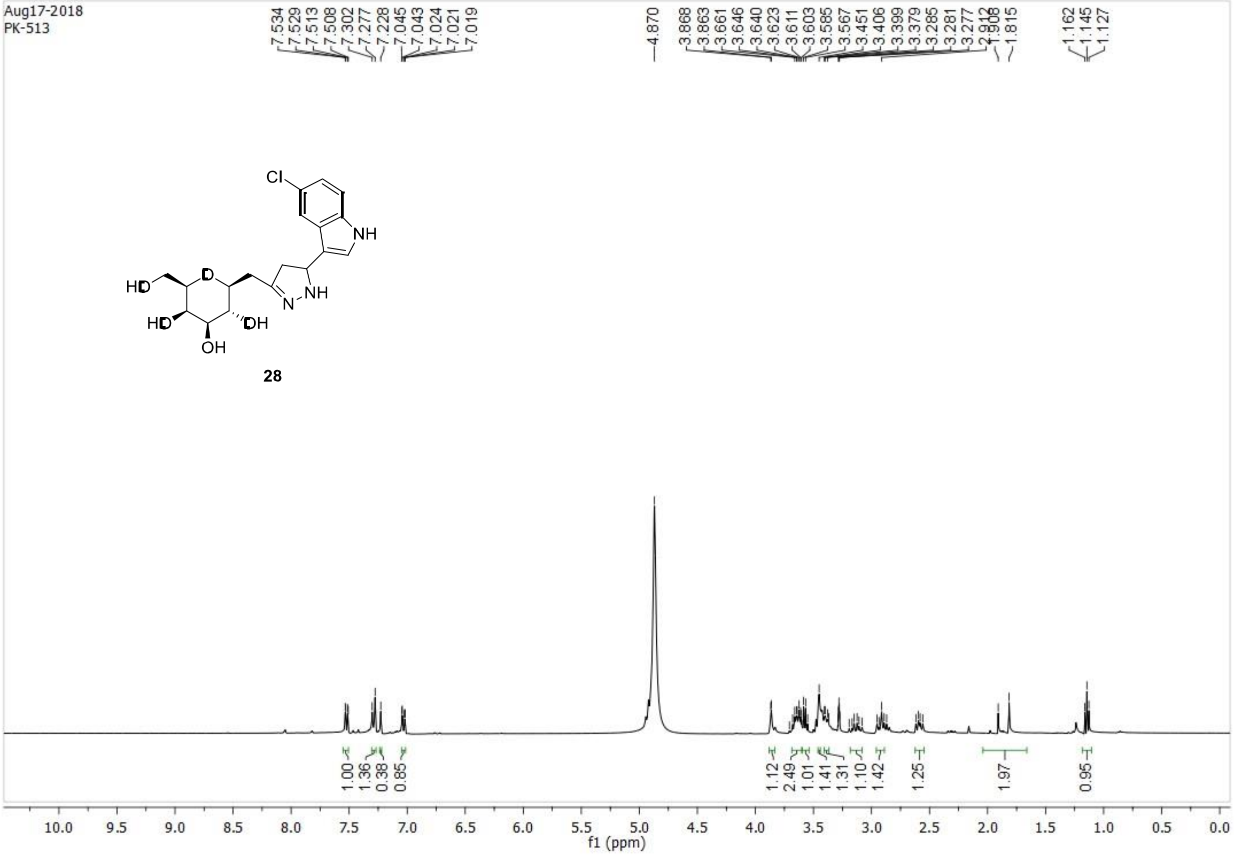
Aug17-2018
PK-513
7.534
7.529
7.513
7.508
7.302
7.277
7.228
7.045
7.043
7.024
7.021
7.019
4.870
3.868
3.863
3.661
3.646
3.640
3.623
3.611
3.603
3.585
3.567
3.451
3.406
3.399
3.379
3.285
3.281
3.277
2.912
1.908
1.815
1.162
1.145
1.127
Cl
NH
HO
O
N
NH
HO
OH
OH
28
1.00
1.36
0.38
0.85
1.12
2.49
1.01
1.41
1.31
1.10
1.42
1.25
1.97
0.95
10.0 9.5 9.0 8.5 8.0 7.5 7.0 6.5 6.0 5.5 5.0 4.5 4.0 3.5 3.0 2.5 2.0 1.5 1.0 0.5 0.0
f1 (ppm)

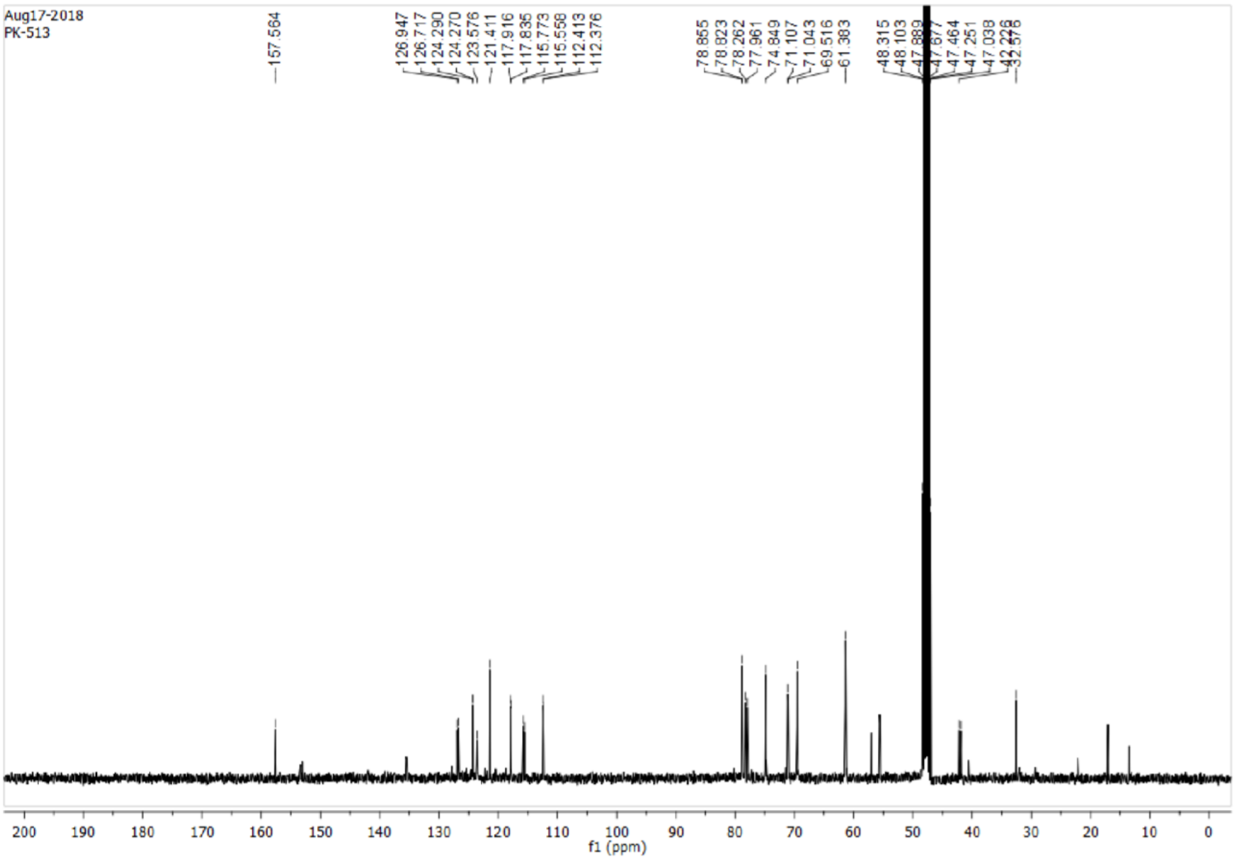
Aug17-2018
PK-513
157.564
126.947
126.717
124.290
124.270
123.576
121.411
117.916
117.835
115.773
115.558
112.413
112.376
78.855
78.823
78.262
77.961
74.849
71.107
71.043
69.516
61.383
48.315
48.103
47.889
47.677
47.464
47.251
47.038
42.226
32.576
200 190 180 170 160 150 140 130 120 110 100 90 80 70 60 50 40 30 20 10 0
f1 (ppm)

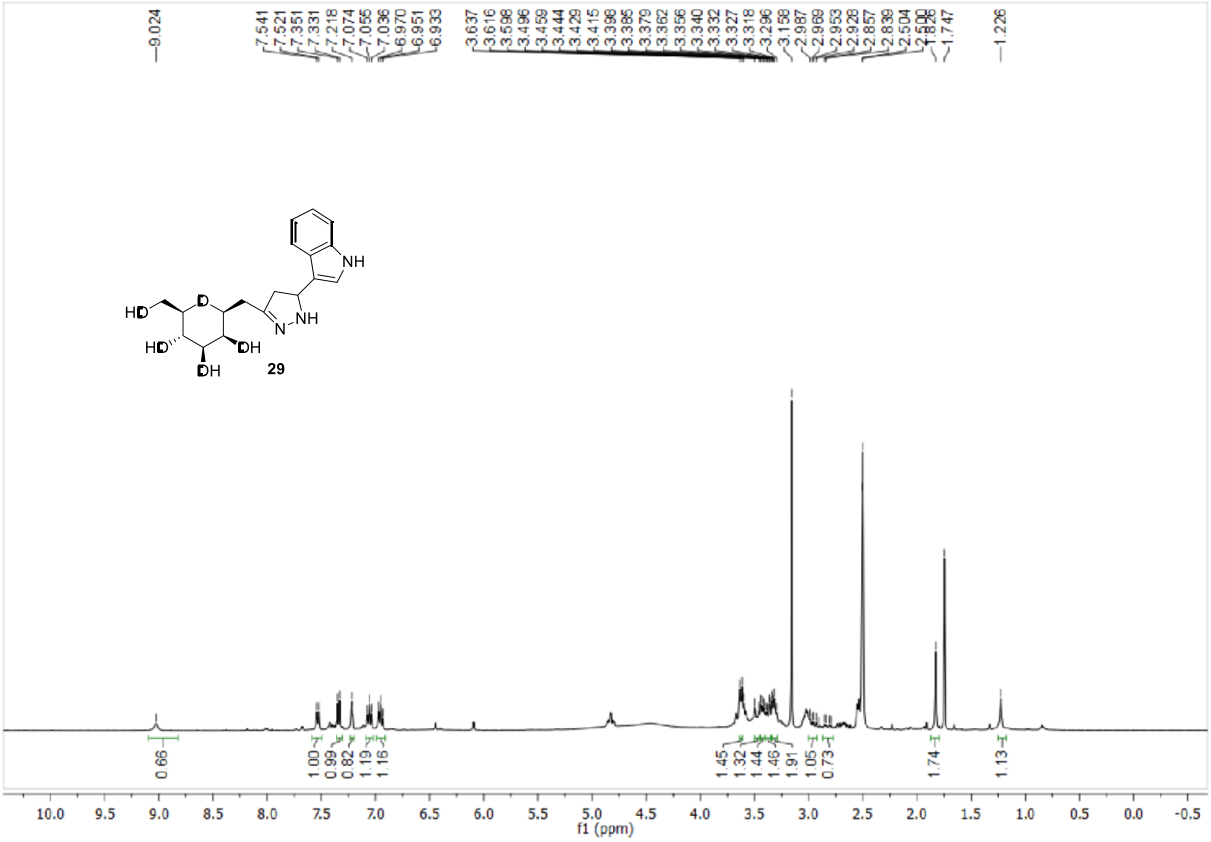
9.024
7.541 7.521 7.351 7.331 7.218 7.074 7.055 7.036 6.970 6.951 6.933
3.637 3.616 3.598 3.496 3.459 3.444 3.429 3.415 3.398 3.385 3.379 3.362 3.356 3.340 3.332 3.327 3.318 3.296 3.158 2.987 2.969 2.953 2.928 2.857 2.839 2.504 2.500
1.747
1.226
HO
O
HO
OH
OH
NH
N
NH
29
0.66
1.00 0.99 0.82 1.19 1.16
1.45 1.32 1.44 1.46 1.91 1.05 0.73
1.74
1.13
f1 (ppm)

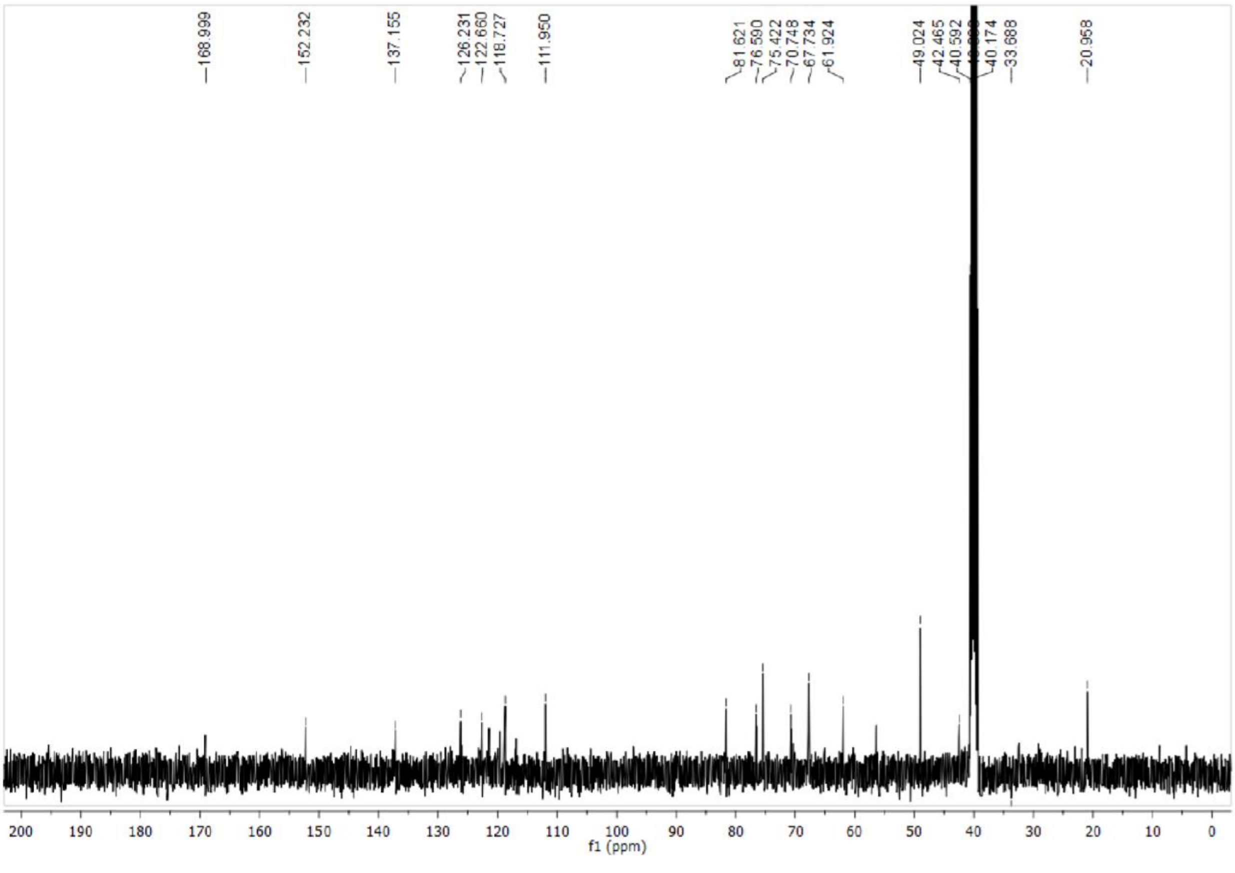
168.999
152.232
137.155
126.231 122.660 118.727
111.950
81.621 76.590 75.422 70.748 67.734 61.924
49.024 42.465 40.592 40.174 33.688
20.958
f1 (ppm)

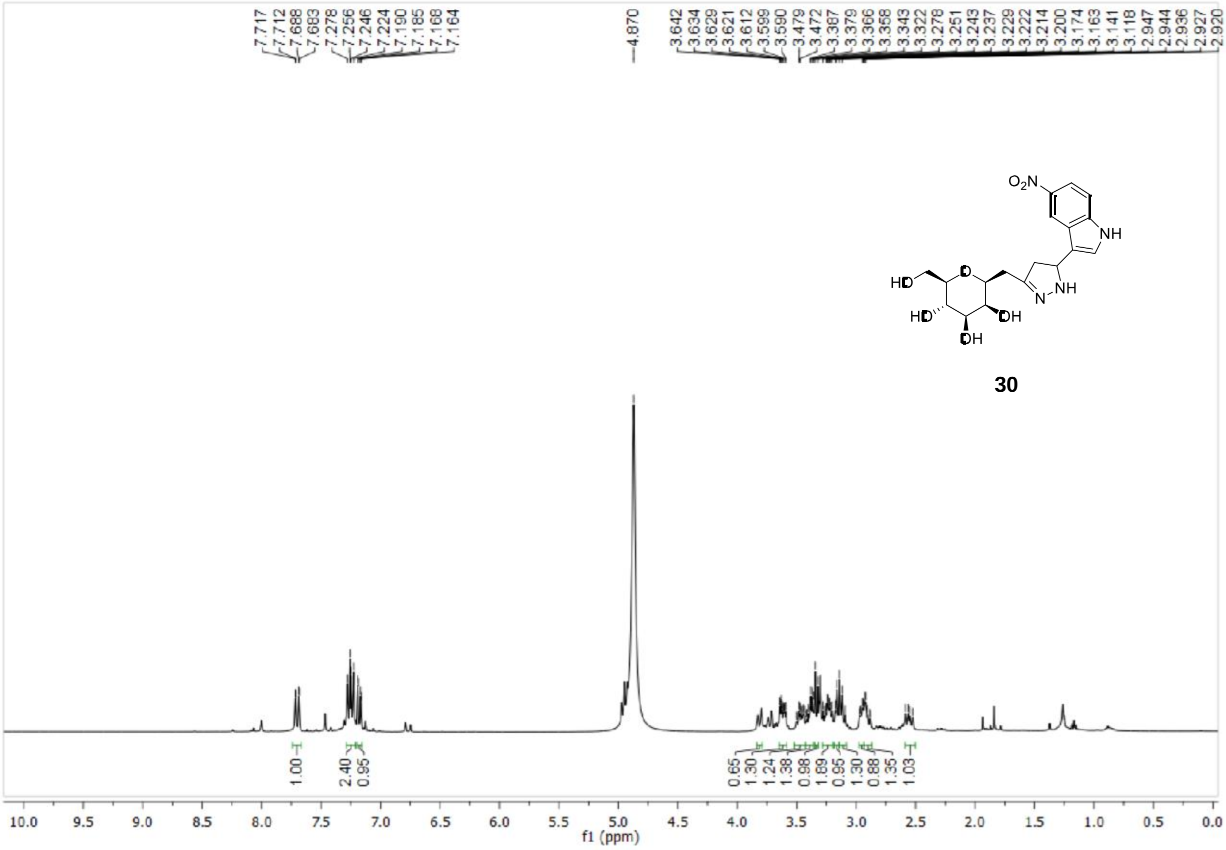
7.717
7.712
7.688
7.683
7.278
7.256
7.246
7.224
7.190
7.185
7.168
7.164
4.870
3.642
3.634
3.629
3.621
3.612
3.599
3.590
3.479
3.472
3.387
3.379
3.366
3.358
3.343
3.322
3.276
3.251
3.243
3.237
3.229
3.222
3.214
3.200
3.174
3.163
3.141
3.118
2.947
2.944
2.936
2.927
2.920
O_2N
NH
HO
HO
OH
OH
O
N
NH
30
1.00
2.40
0.95
0.65
1.30
1.24
1.38
0.98
1.89
0.95
1.30
0.88
1.35
1.03
10.0
9.5
9.0
8.5
8.0
7.5
7.0
6.5
6.0
5.5
5.0
4.5
4.0
3.5
3.0
2.5
2.0
1.5
1.0
0.5
0.0
f1 (ppm)

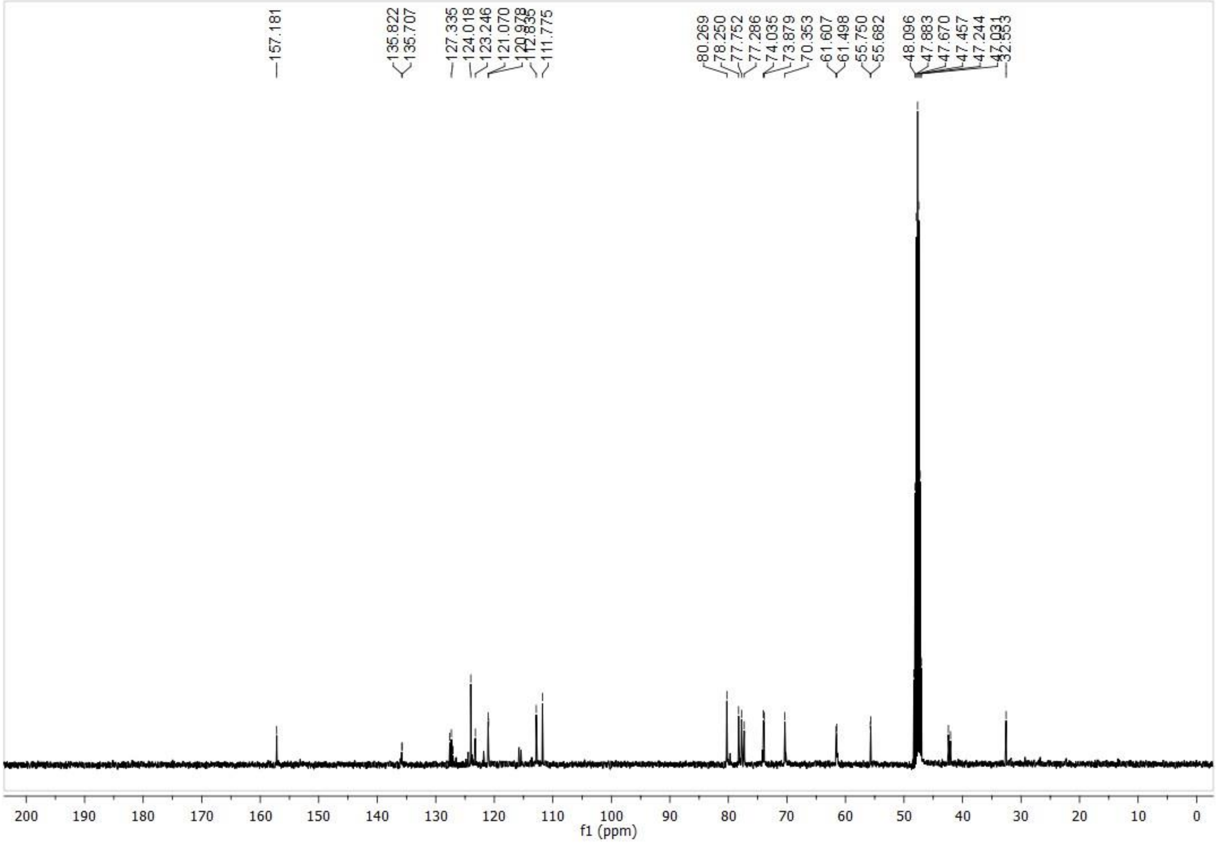
157.181
135.822
135.707
127.335
124.018
123.246
121.070
120.978
112.835
111.775
80.269
78.250
77.752
77.286
74.035
73.879
70.353
61.607
61.498
55.750
55.682
48.096
47.883
47.670
47.457
47.244
47.031
32.553
200
190
180
170
160
150
140
130
120
110
100
90
80
70
60
50
40
30
20
10
0
f1 (ppm)

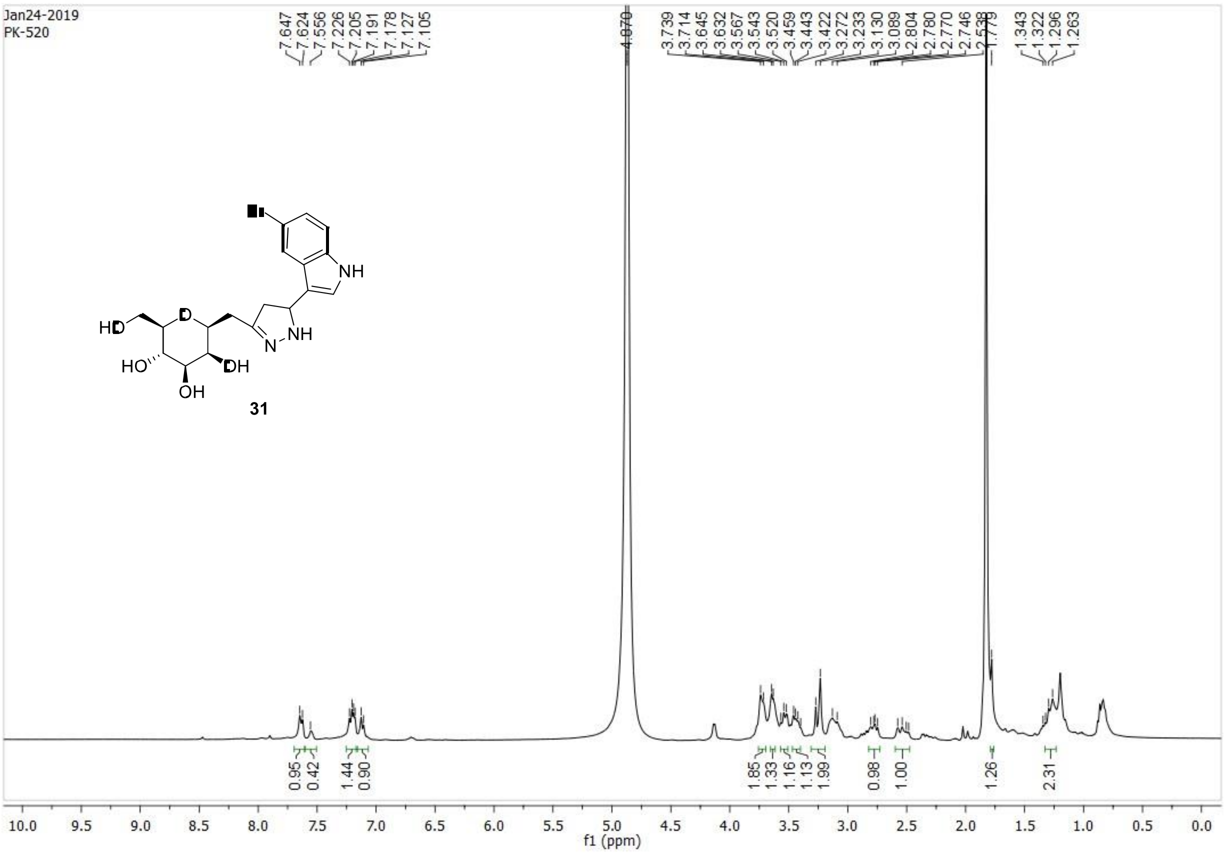

Jan24-2019
PK-520
7.647
7.624
7.556
7.226
7.205
7.191
7.178
7.127
7.105
4.870
3.739
3.714
3.645
3.632
3.567
3.543
3.520
3.459
3.443
3.422
3.272
3.233
3.130
3.089
2.804
2.780
2.770
2.746
1.779
1.343
1.322
1.296
1.263
NH
HO
O
N
NH
HO
OH
OH
31
0.95
0.42
1.44
0.90
1.85
1.33
1.16
1.13
1.99
0.98
1.00
1.26
2.31
10.0 9.5 9.0 8.5 8.0 7.5 7.0 6.5 6.0 5.5 5.0 4.5 4.0 3.5 3.0 2.5 2.0 1.5 1.0 0.5 0.0
f1 (ppm)

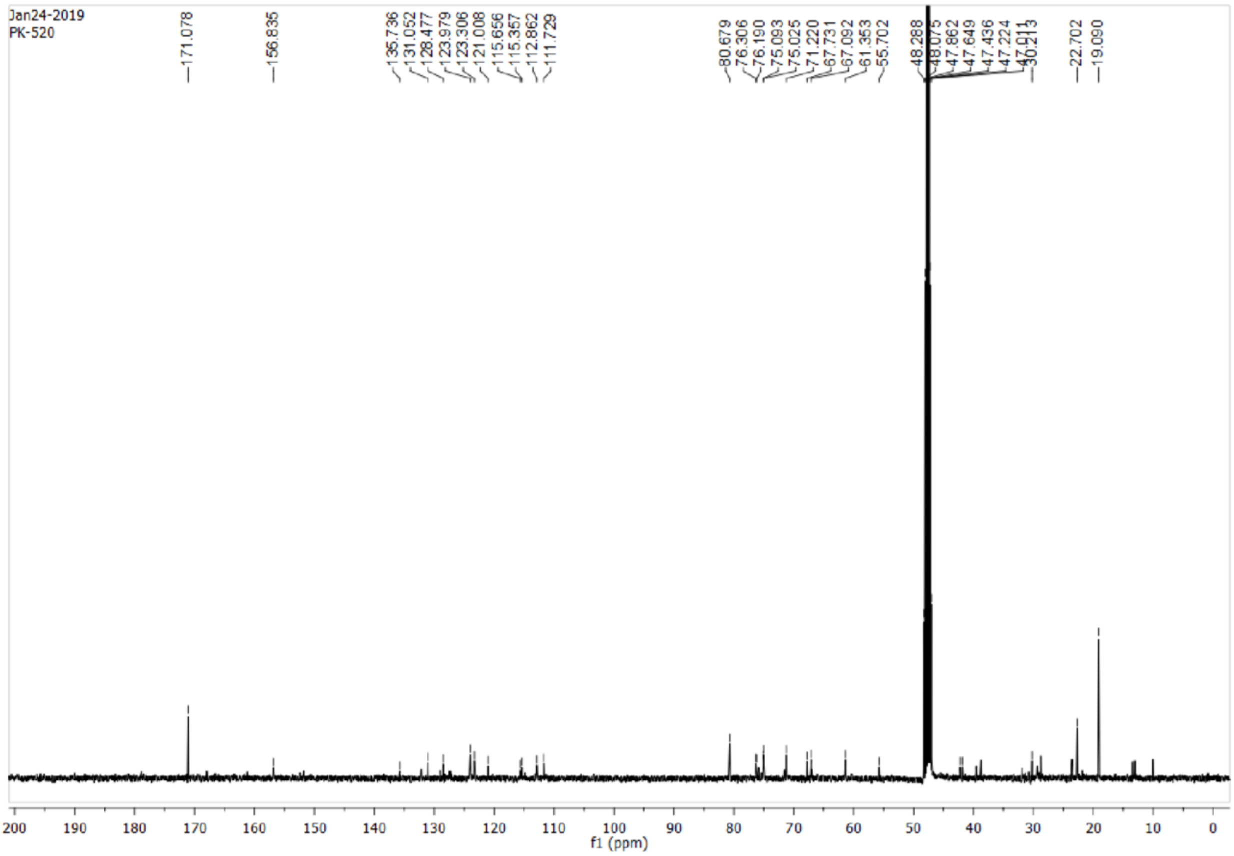

Jan24-2019
PK-520
171.078
156.835
135.736
131.052
128.477
123.979
123.306
121.008
115.656
115.357
112.862
111.729
80.679
76.306
76.190
75.093
75.025
71.220
67.731
67.092
61.353
55.702
48.288
48.075
47.862
47.649
47.436
47.224
47.011
30.213
22.702
19.090
200 190 180 170 160 150 140 130 120 110 100 90 80 70 60 50 40 30 20 10 0
f1 (ppm)

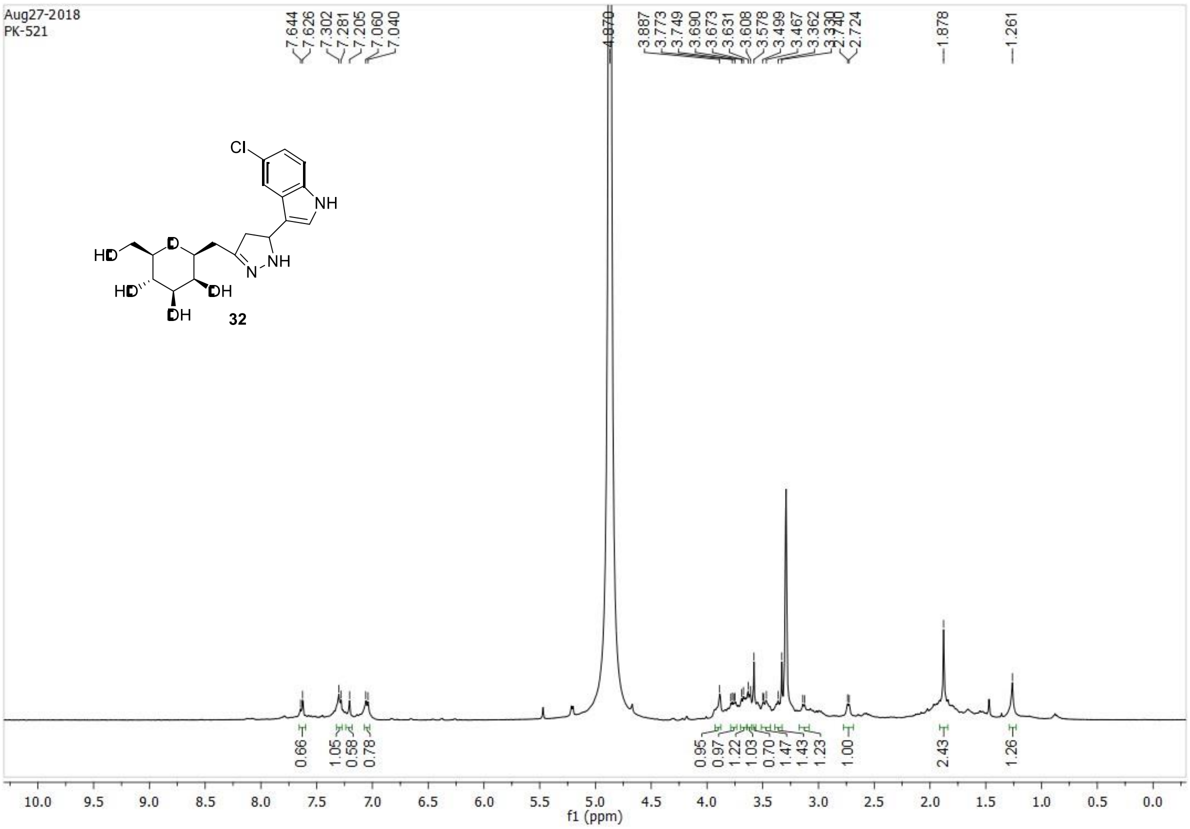
Aug27-2018
PK-521
7.644
7.626
7.302
7.281
7.205
7.060
7.040
4.870
3.887
3.773
3.749
3.690
3.673
3.631
3.608
3.578
3.499
3.467
3.362
2.724
1.878
1.261
Cl
NH
HO
O
N
NH
HO
OH
OH
32
0.66
1.05
0.58
0.78
0.95
0.97
1.22
1.03
0.70
1.47
1.43
1.23
1.00
2.43
1.26
f1 (ppm)

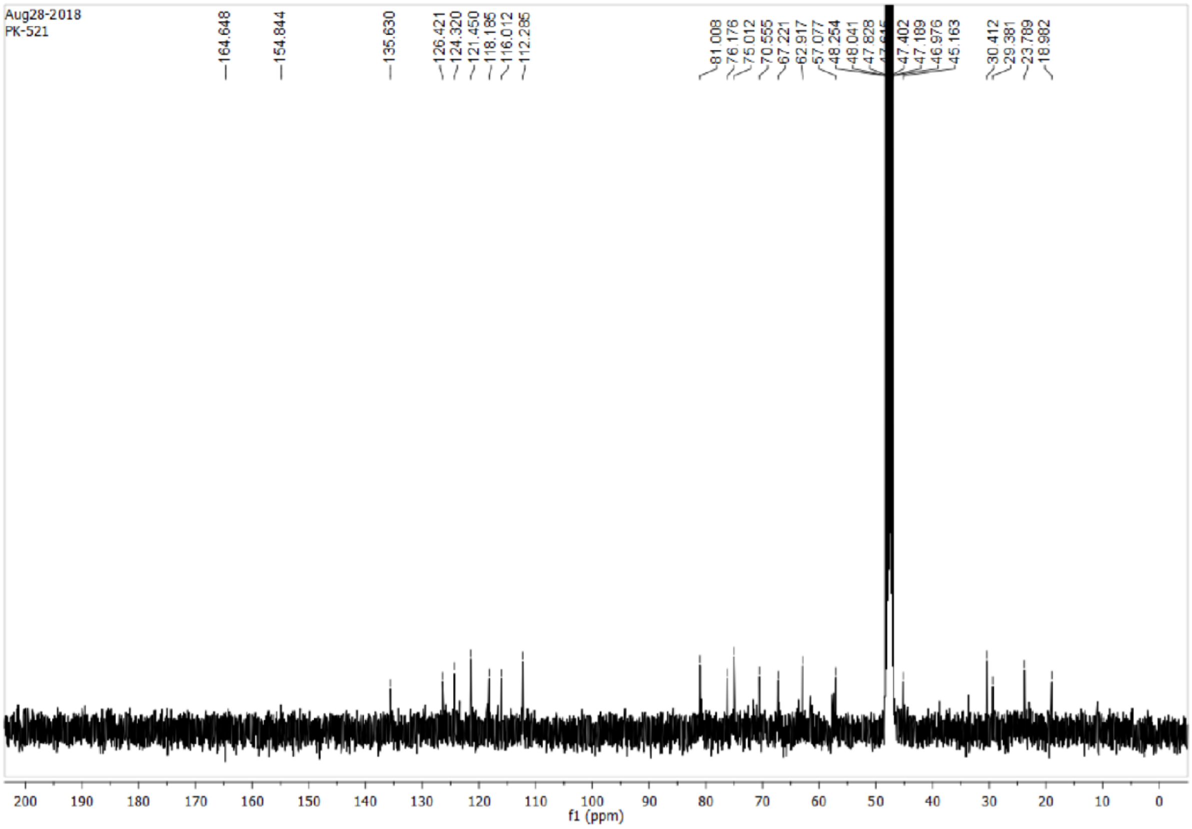
Aug28-2018
PK-521
164.648
154.844
135.630
126.421
124.320
121.450
118.185
116.012
112.285
81.008
76.176
75.012
70.555
67.221
62.917
57.077
48.254
48.041
47.828
47.402
47.189
46.976
45.163
30.412
29.381
23.789
18.982
f1 (ppm)

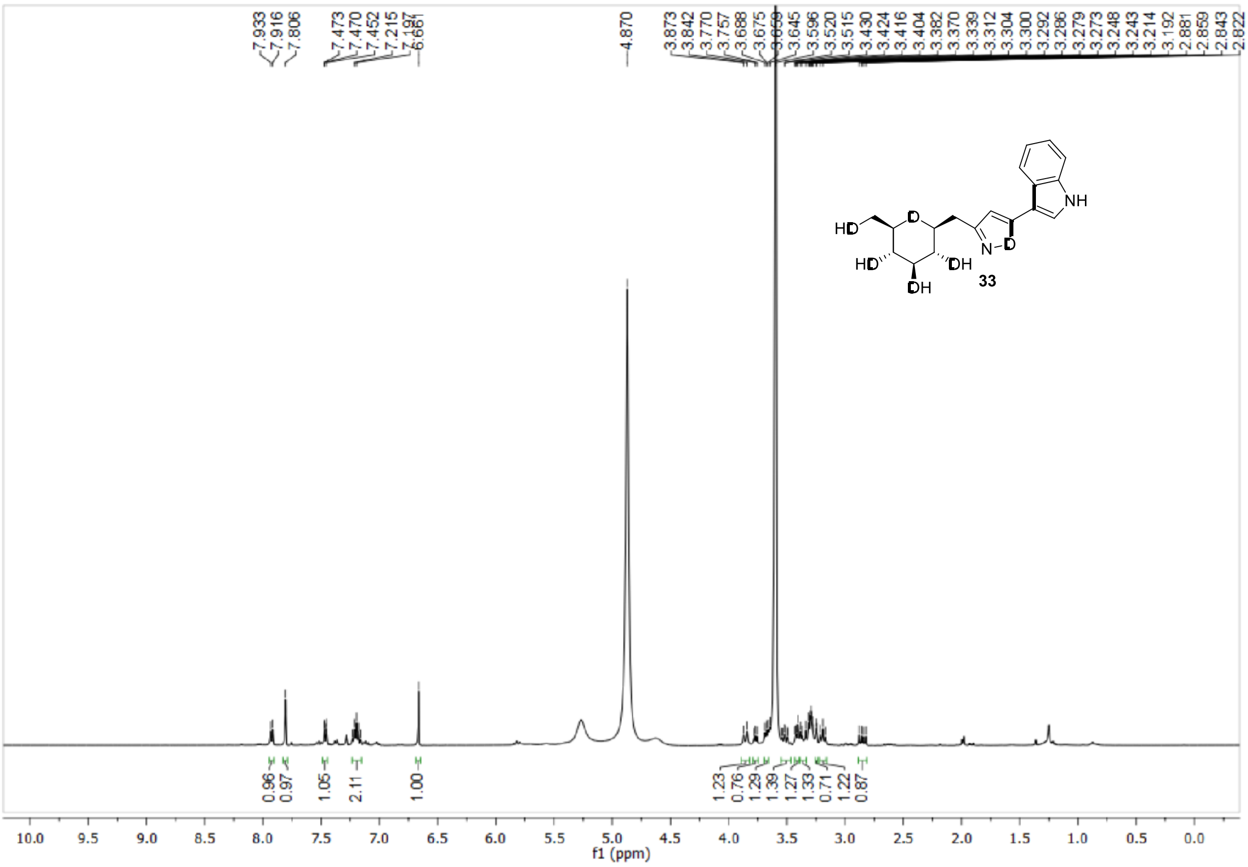
7.933
7.916
7.806
7.473
7.470
7.452
7.215
4.870
3.873
3.842
3.770
3.757
3.688
3.675
3.645
3.596
3.520
3.515
3.430
3.424
3.416
3.404
3.382
3.370
3.339
3.312
3.304
3.300
3.292
3.286
3.279
3.273
3.248
3.243
3.214
3.192
2.881
2.859
2.843
2.822
HO
O
OH
OH
HO
N
O
NH
33
0.96
0.97
1.05
2.11
1.00
1.23
0.76
1.29
1.39
1.27
1.33
0.71
1.22
0.87
f1 (ppm)

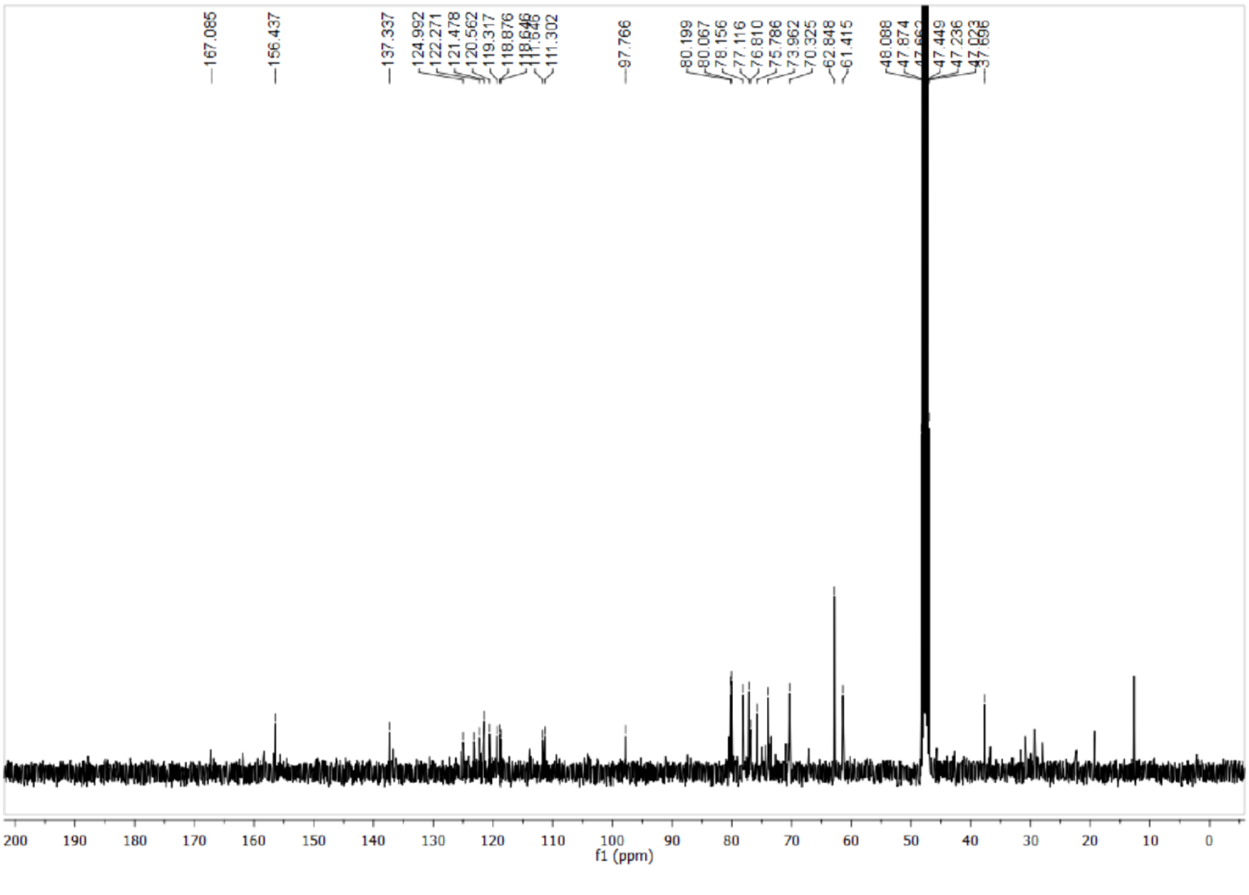
167.085
156.437
137.337
124.992
122.271
121.478
120.562
119.317
118.876
111.302
97.766
80.199
80.067
78.156
77.116
76.810
75.786
73.962
70.325
62.848
61.415
48.088
47.874
47.449
47.236
f1 (ppm)

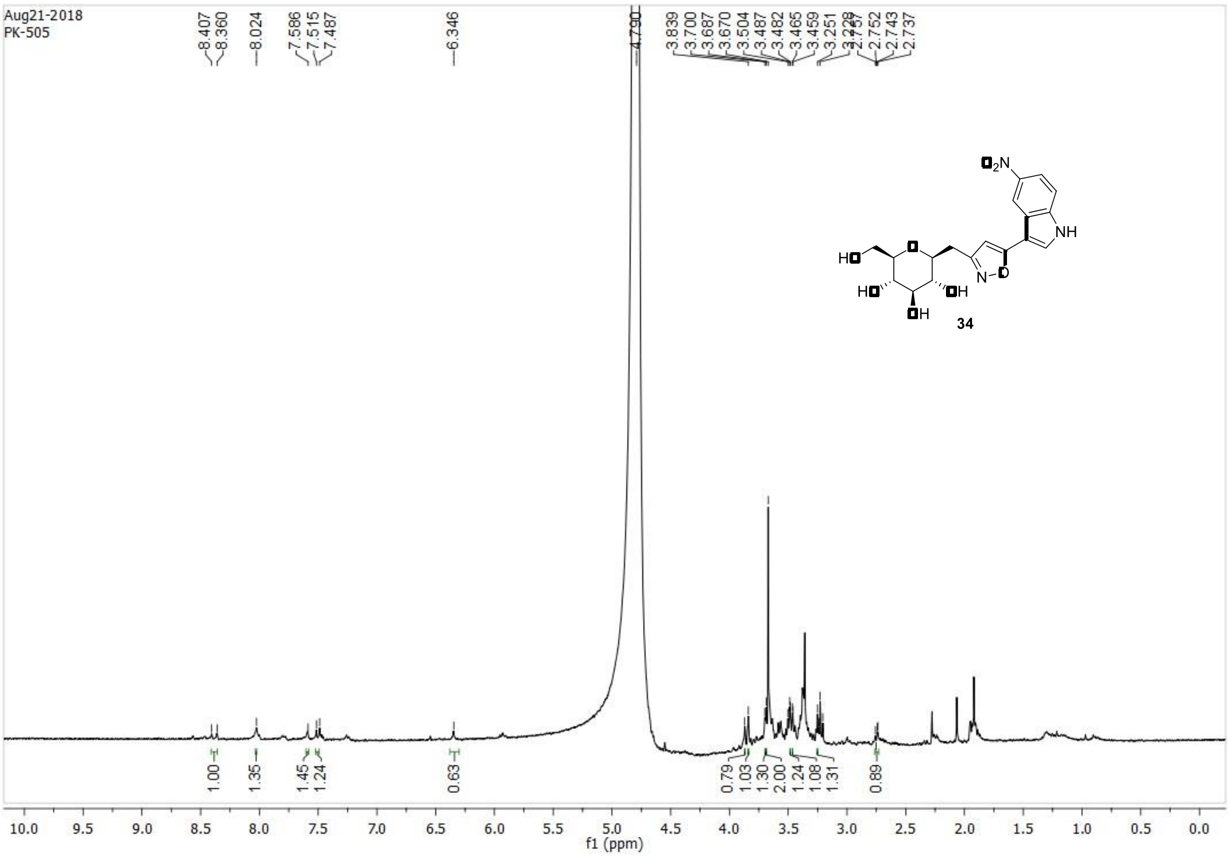
Aug21-2018
PK-505
8.407
8.360
8.024
7.586
7.515
7.487
6.346
3.839
3.700
3.687
3.670
3.504
3.487
3.482
3.465
3.459
3.251
2.752
2.743
2.737
1.00
1.35
1.45
1.24
0.63
0.79
1.03
1.30
2.00
1.24
1.08
1.31
0.89
NH
34
f1 (ppm)

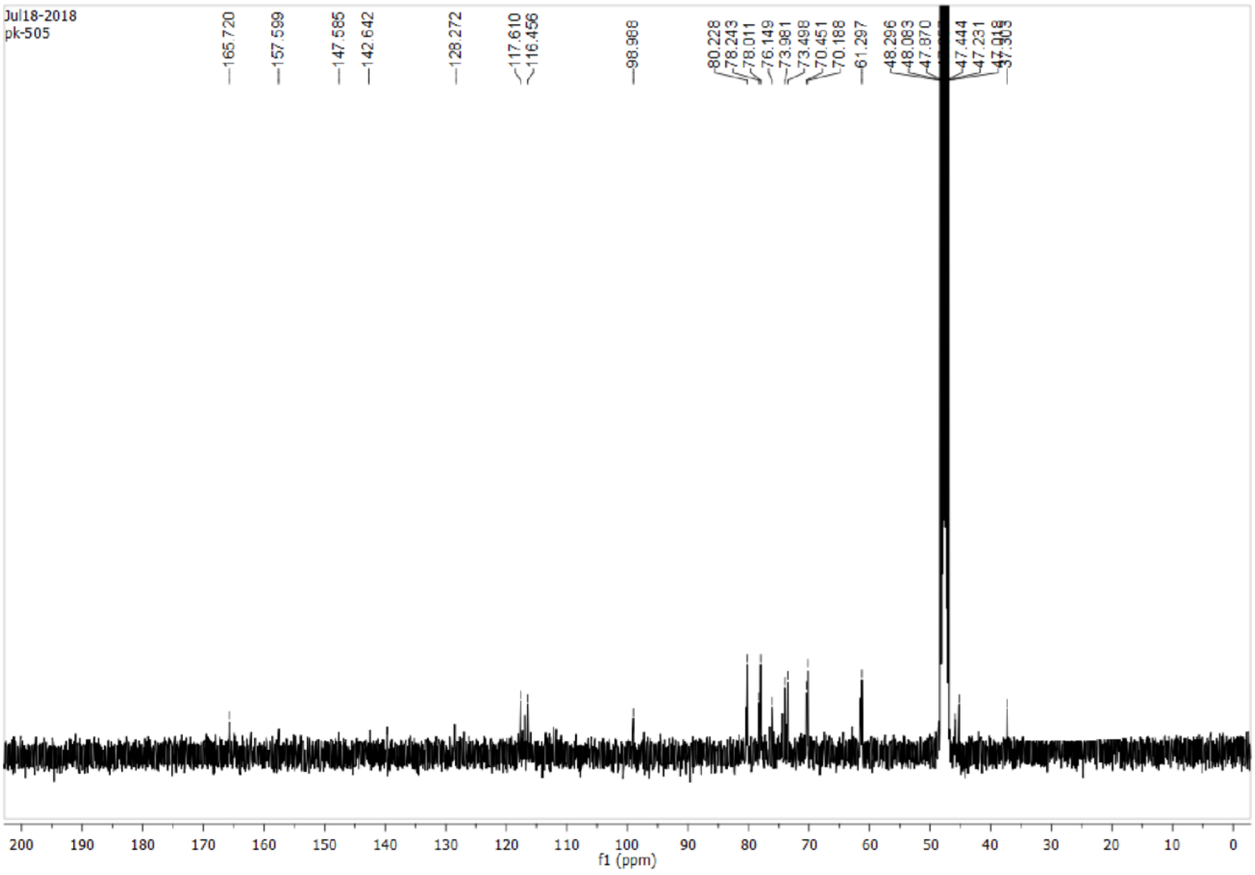
Jul18-2018
pk-505
165.720
157.599
147.585
142.642
128.272
117.610
116.456
98.988
80.228
78.243
78.011
76.149
73.981
73.498
70.451
70.188
61.297
48.296
48.083
47.870
47.444
47.231
f1 (ppm)

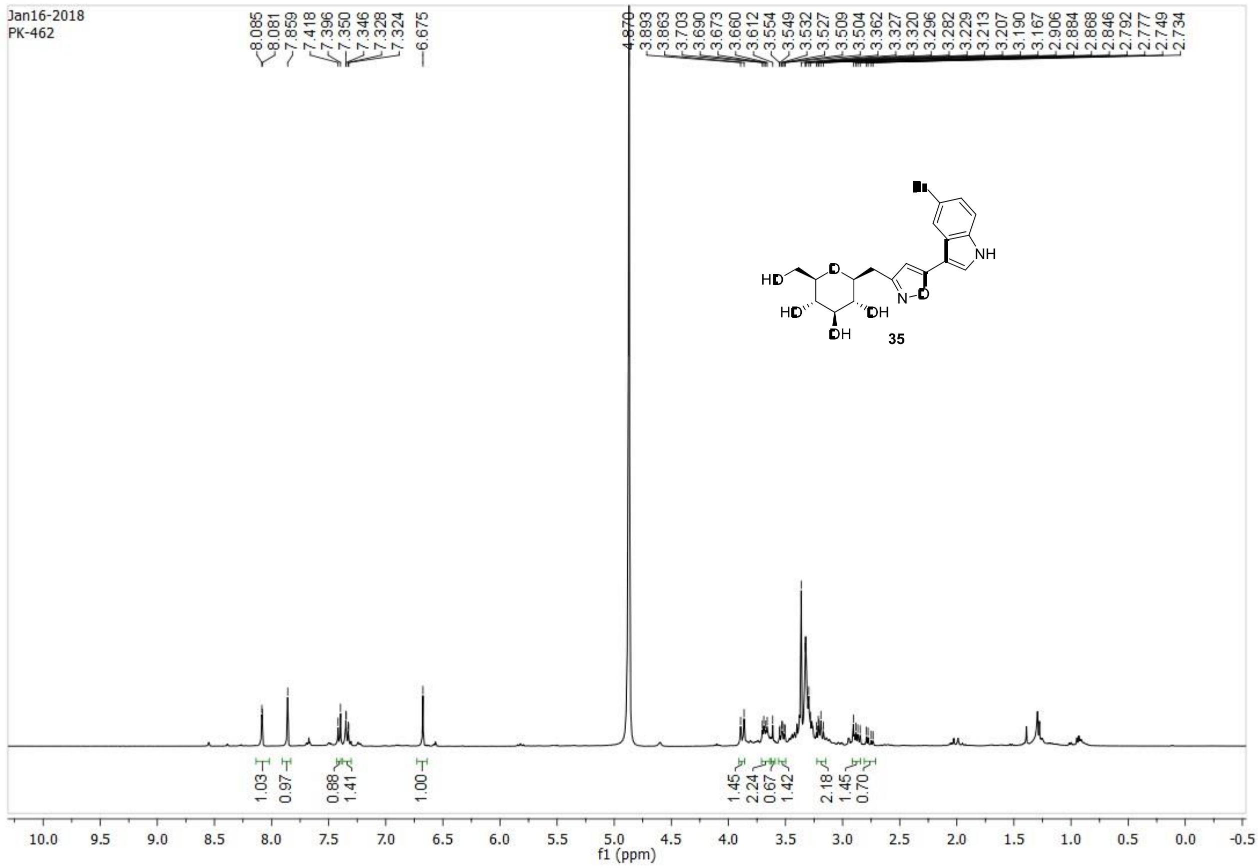
Jan16-2018
PK-462
8.085
8.081
7.859
7.418
7.396
7.350
7.346
7.328
7.324
6.675
4.870
3.893
3.863
3.703
3.690
3.673
3.660
3.612
3.554
3.549
3.532
3.527
3.509
3.504
3.362
3.327
3.320
3.296
3.282
3.229
3.213
3.207
3.190
3.167
2.906
2.884
2.868
2.846
2.792
2.777
2.749
2.734
NH
HO
HO
OH
OH
N
35
1.03
0.97
0.88
1.41
1.00
1.45
2.24
0.67
1.42
2.18
1.45
0.70
f1 (ppm)

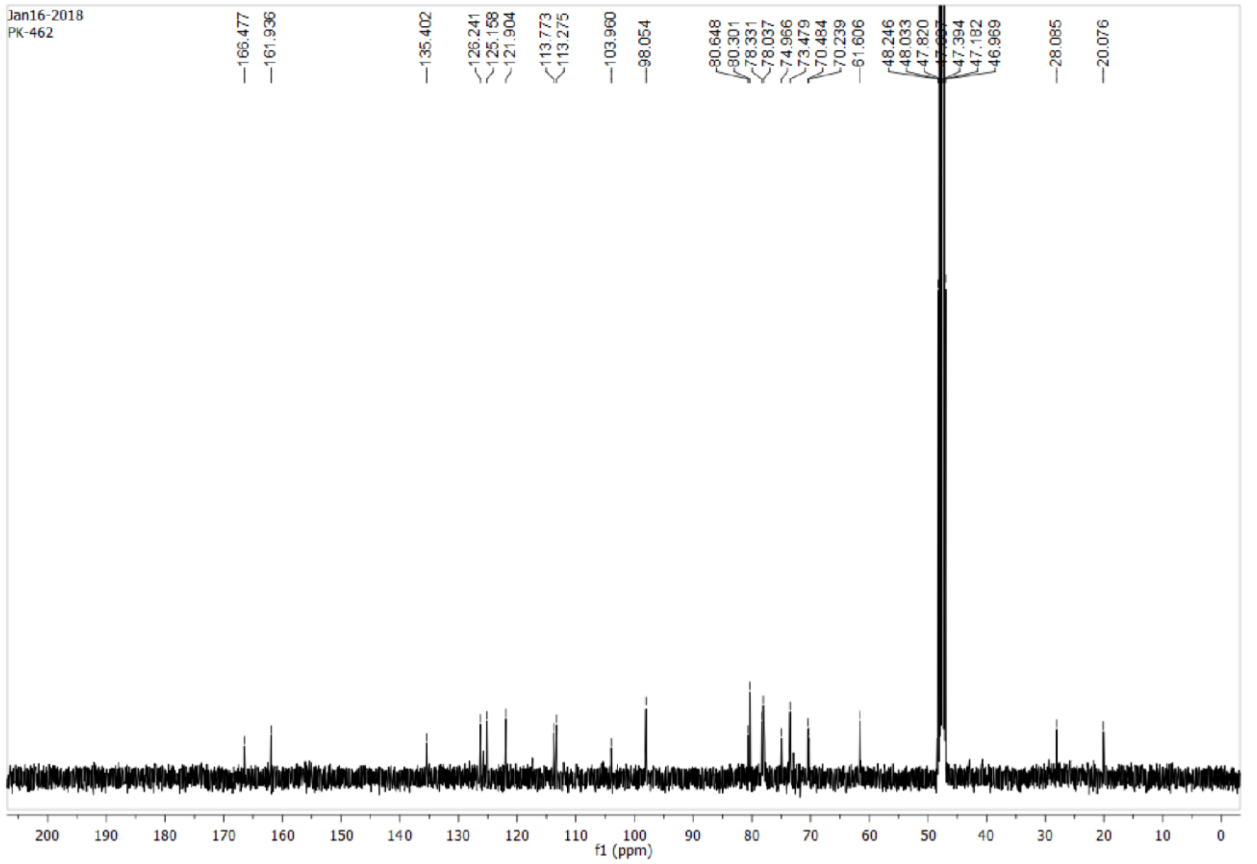
Jan16-2018
PK-462
166.477
161.936
135.402
126.241
125.158
121.904
113.773
113.275
103.960
98.054
80.648
80.301
78.331
78.037
74.966
73.479
70.484
70.239
61.606
48.246
48.033
47.820
47.394
47.182
46.969
28.085
20.076
f1 (ppm)

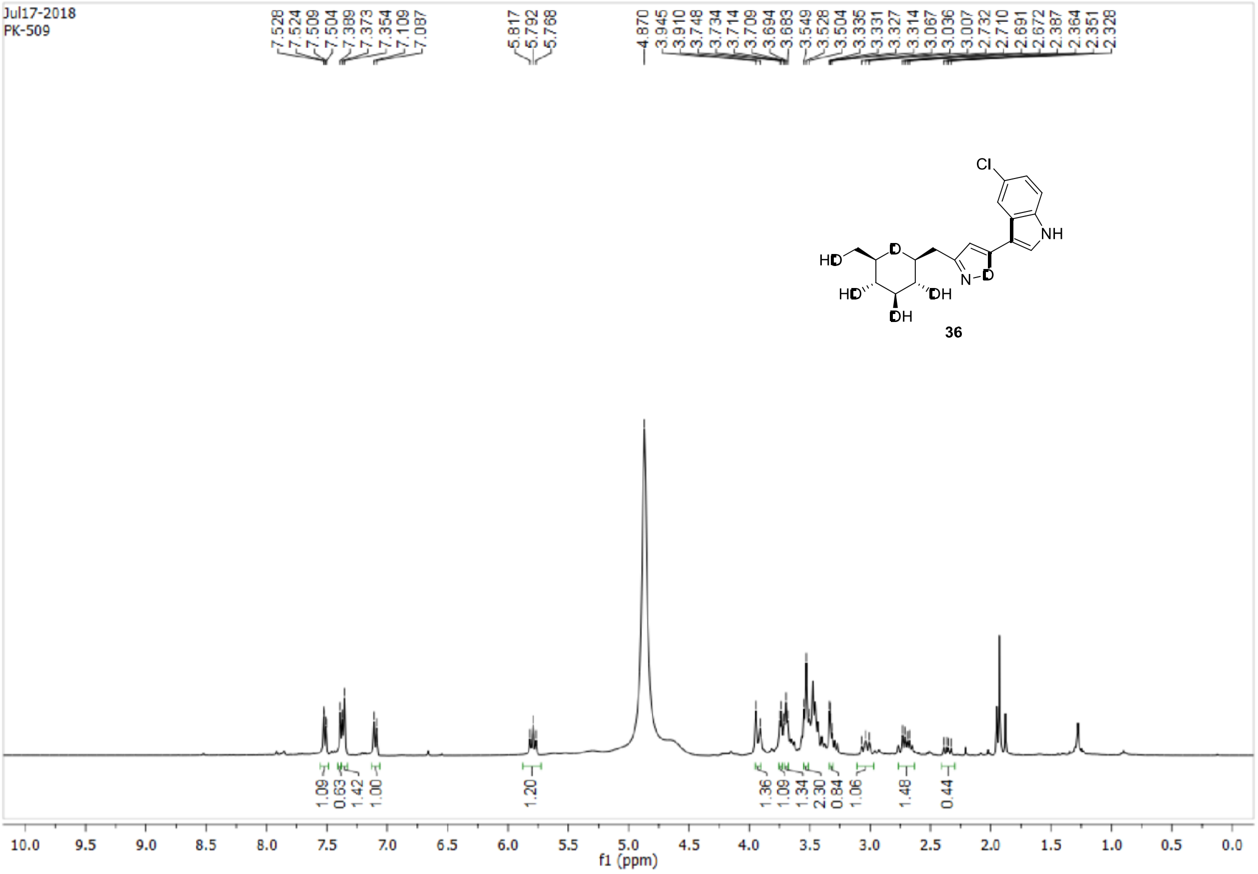

Jul17-2018
PK-509
7.528 7.524 7.509 7.504 7.389 7.373 7.354 7.109 7.087
5.817 5.792 5.768
4.870 3.945 3.910 3.748 3.734 3.714 3.709 3.694 3.683 3.549 3.528 3.504 3.335 3.331 3.327 3.314 3.067 3.036 3.007 2.732 2.710 2.691 2.672 2.387 2.364 2.351 2.328
Cl
NH
HO
O
N
OH
36
1.09 0.63 1.42 1.00 1.20 1.36 1.09 1.34 2.30 0.84 1.06 1.48 0.44
f1 (ppm)

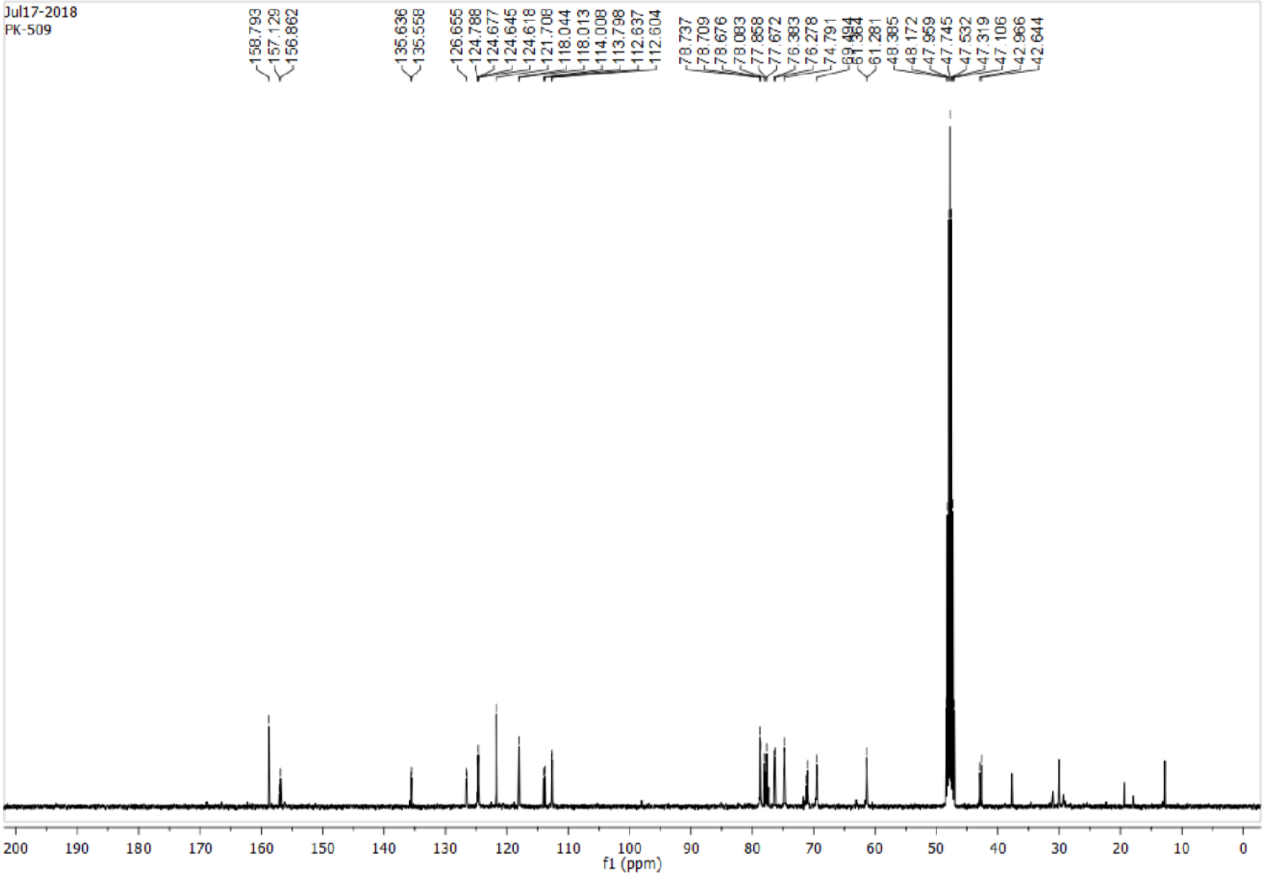

Jul17-2018
PK-509
158.793 157.129 156.862
135.636 135.558
126.655 124.788 124.677 124.645 124.618 121.708 118.044 118.013 114.008 113.798 112.637 112.604
78.737 78.709 78.676 78.083 77.858 77.672 76.383 76.278 74.791 61.281 48.385 48.172 47.959 47.745 47.532 47.319 47.106 42.966 42.644
f1 (ppm)

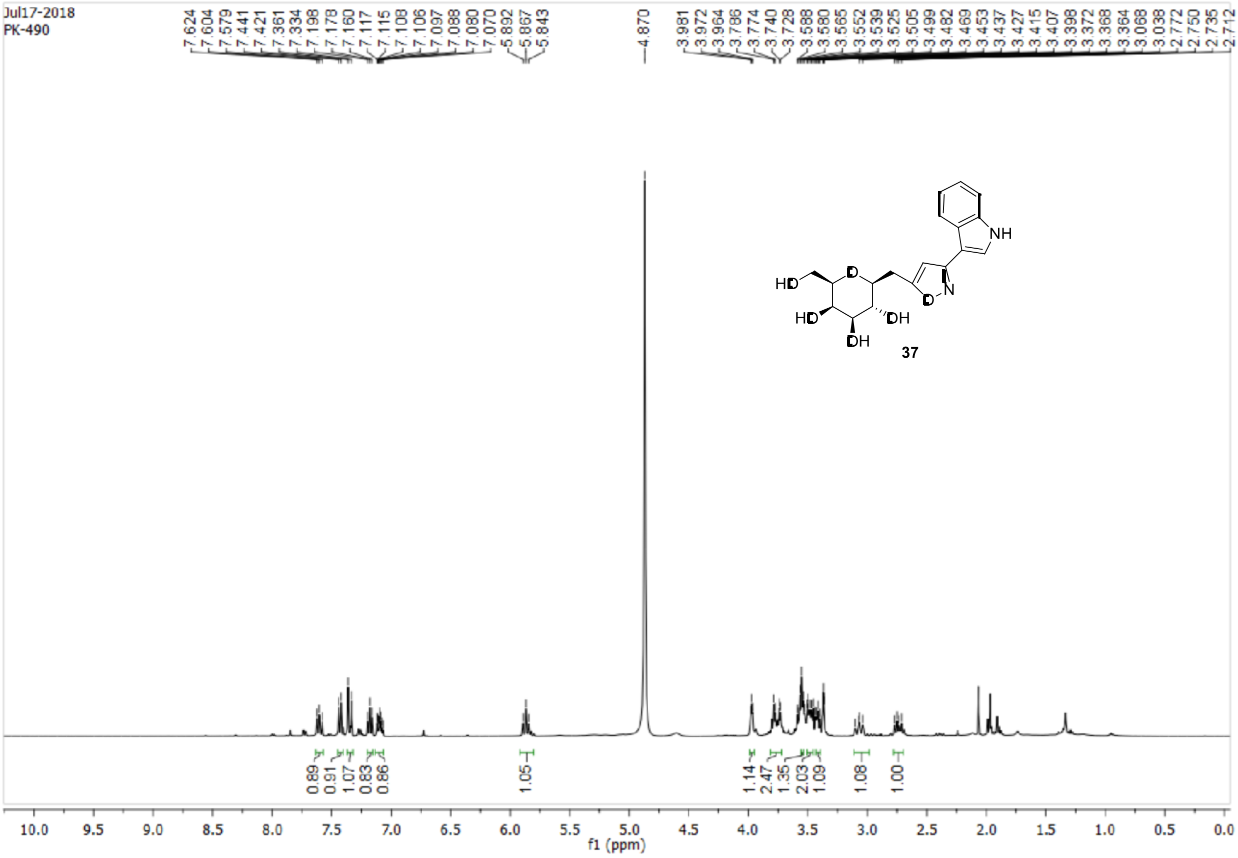

Jul17-2018
PK-490
7.624
7.604
7.579
7.441
7.421
7.361
7.334
7.198
7.178
7.160
7.117
7.115
7.108
7.106
7.097
7.088
7.080
7.070
5.892
5.867
5.843
4.870
3.981
3.972
3.964
3.786
3.774
3.740
3.728
3.588
3.580
3.565
3.552
3.539
3.525
3.505
3.499
3.482
3.469
3.453
3.437
3.427
3.415
3.407
3.398
3.372
3.368
3.364
3.068
3.038
2.772
2.750
2.735
2.712
NH
HO
O
O
N
HO
OH
OH
37
0.89
0.91
1.07
0.83
0.86
1.05
1.14
2.47
1.35
2.03
1.09
1.08
1.00
f1 (ppm)

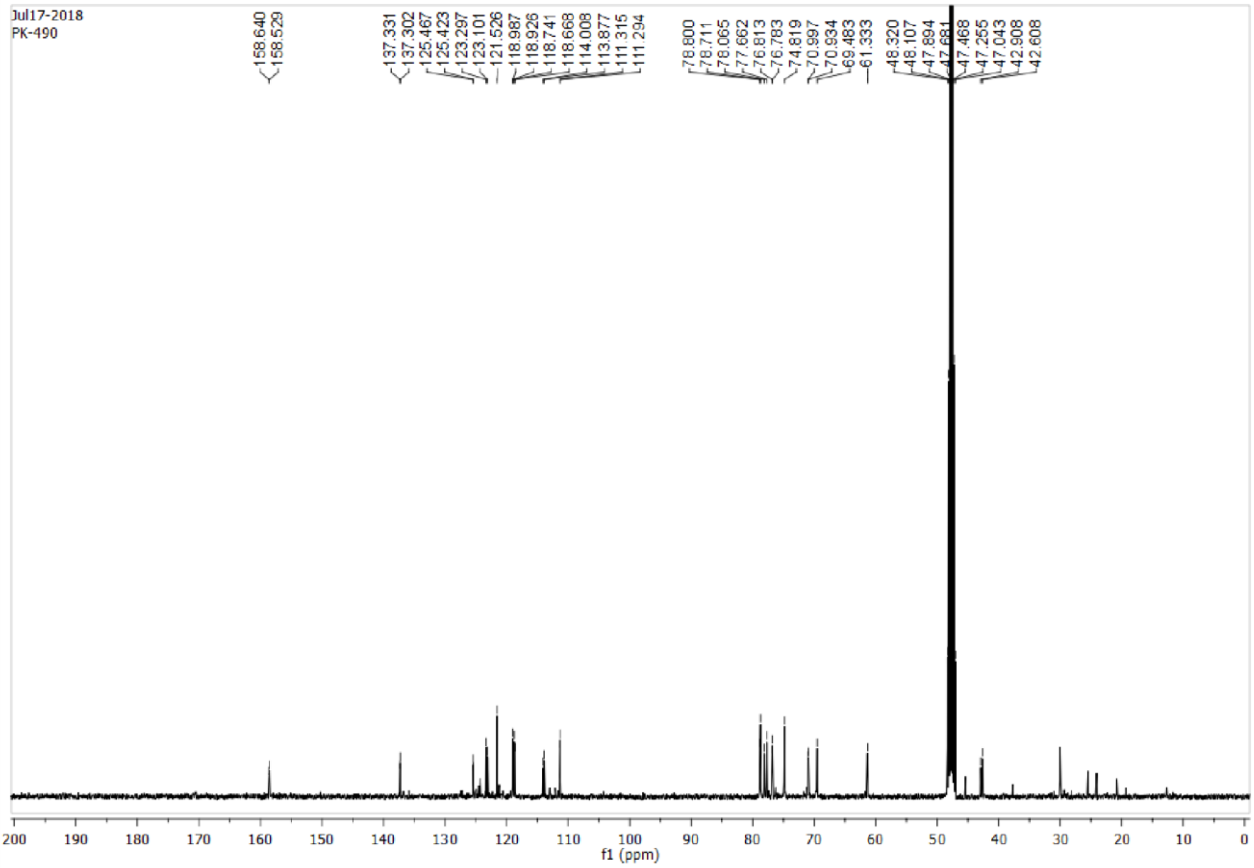

Jul17-2018
PK-490
158.640
158.529
137.331
137.302
125.467
125.423
123.297
123.101
121.526
118.987
118.926
118.741
118.668
114.008
113.877
111.315
111.294
78.800
78.711
78.065
77.662
76.813
76.783
74.819
70.997
70.934
69.483
61.333
48.320
48.107
47.894
47.681
47.468
47.255
47.043
42.908
42.608
f1 (ppm)

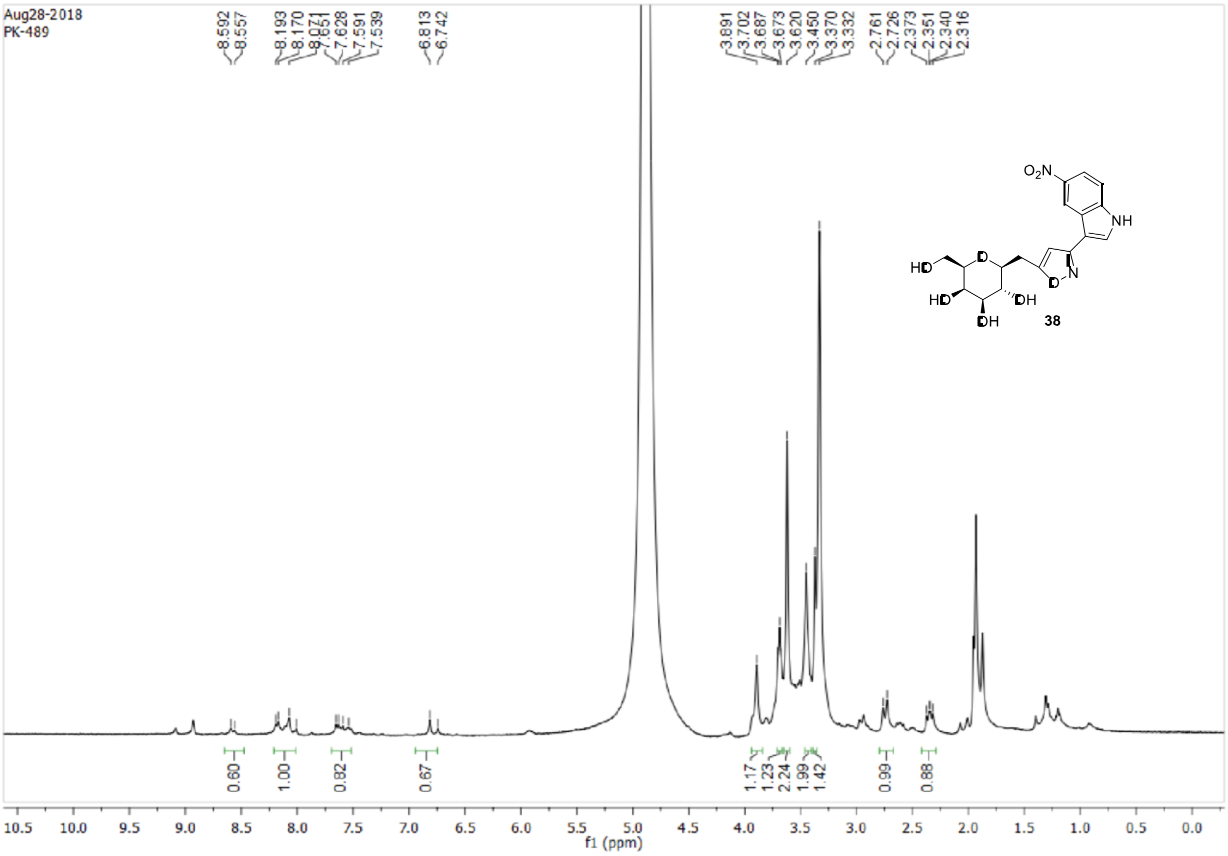

Aug28-2018
PK-489
8.592
8.557
8.193
8.170
8.071
7.651
7.628
7.591
7.539
6.813
6.742
3.891
3.702
3.687
3.673
3.620
3.450
3.370
3.332
2.761
2.726
2.373
2.351
2.340
2.316
0.60
1.00
0.82
0.67
1.17
1.23
2.24
1.99
1.42
0.99
0.88
f1 (ppm)
O2N
NH
HO
O
N
OH
38

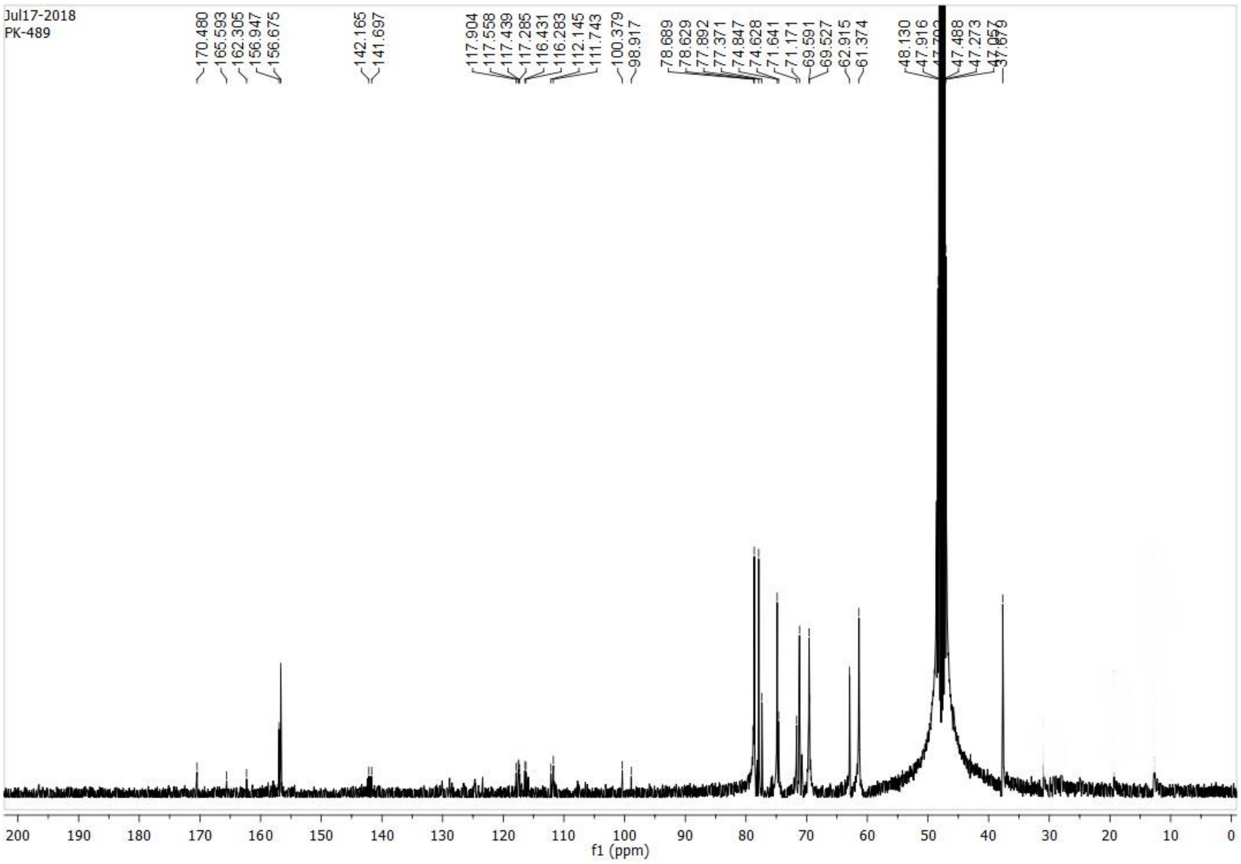

Jul17-2018
PK-489
170.480
165.593
162.305
156.947
156.675
142.165
141.697
117.904
117.558
117.439
117.285
116.431
116.283
112.145
111.743
100.379
98.917
78.689
78.629
77.892
77.371
74.847
74.628
71.641
71.171
69.591
69.527
62.915
61.374
48.130
47.916
47.488
47.273
f1 (ppm)

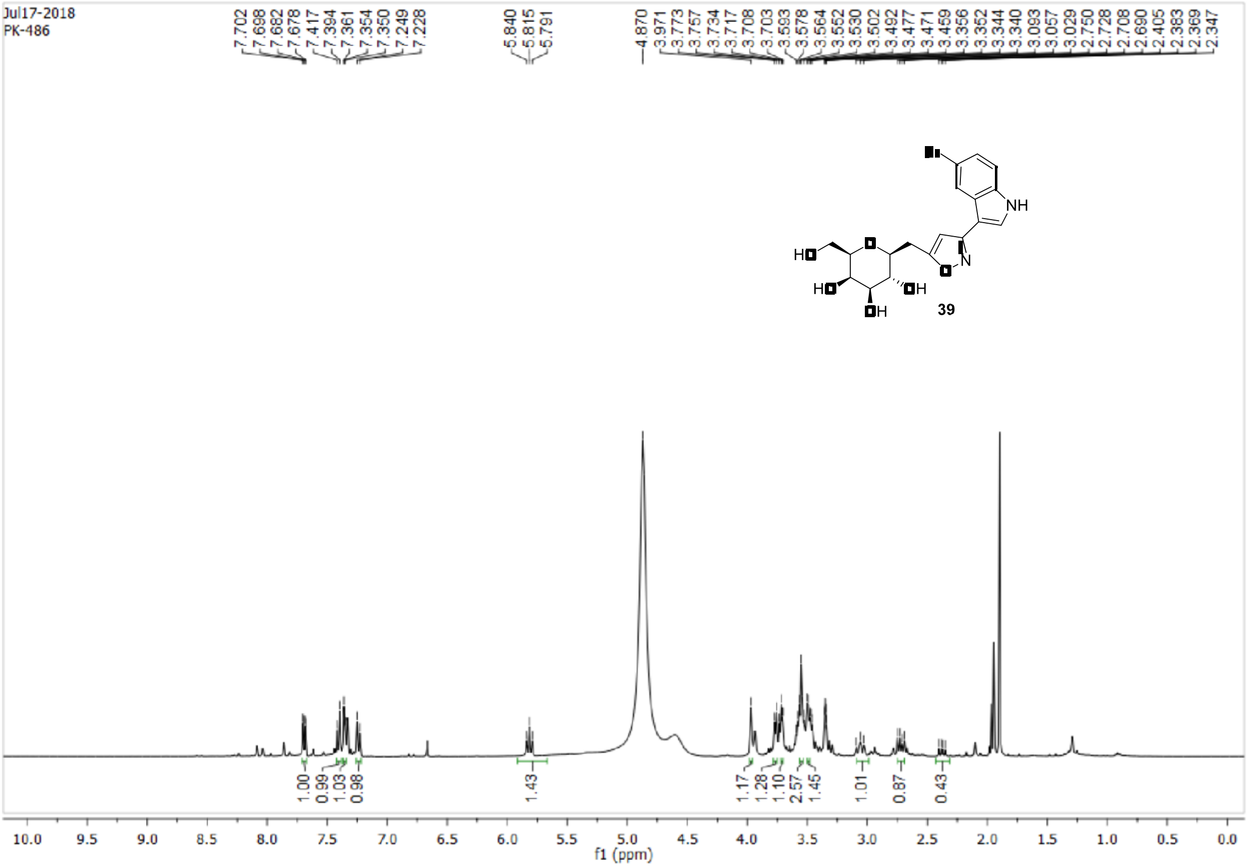
Jul17-2018
PK-486
7.702 7.698 7.682 7.678 7.417 7.394 7.361 7.354 7.350 7.249 7.228
5.840 5.815 5.791
4.870 3.971 3.773 3.757 3.734 3.717 3.708 3.703 3.593 3.578 3.564 3.552 3.530 3.502 3.492 3.477 3.471 3.459 3.356 3.352 3.344 3.340 3.093 3.057 3.029 2.750 2.728 2.708 2.690 2.405 2.383 2.369 2.347
NH
N
39
1.00 0.99 1.03 0.98
1.43
1.17 1.28 1.10 2.57 1.45
1.01
0.87
0.43
f1 (ppm)

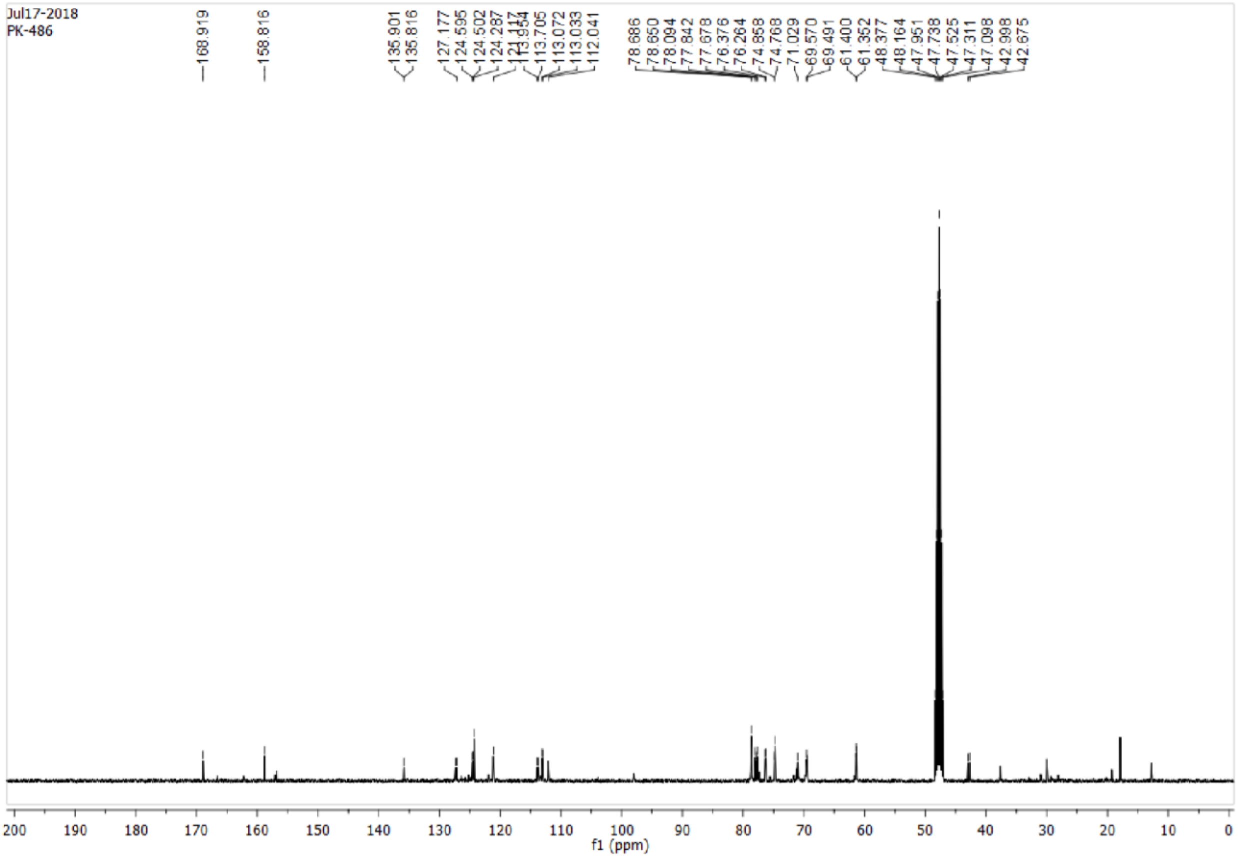
Jul17-2018
PK-486
168.919
158.816
135.901 135.816
127.177 124.595 124.502 124.287 121.147 113.954 113.705 113.072 113.033 112.041
78.686 78.650 78.094 77.842 77.678 76.376 76.264 74.858 74.768 71.029 69.570 69.491 61.400 61.352 48.377 48.164 47.951 47.738 47.525 47.311 47.098 42.998 42.675
f1 (ppm)

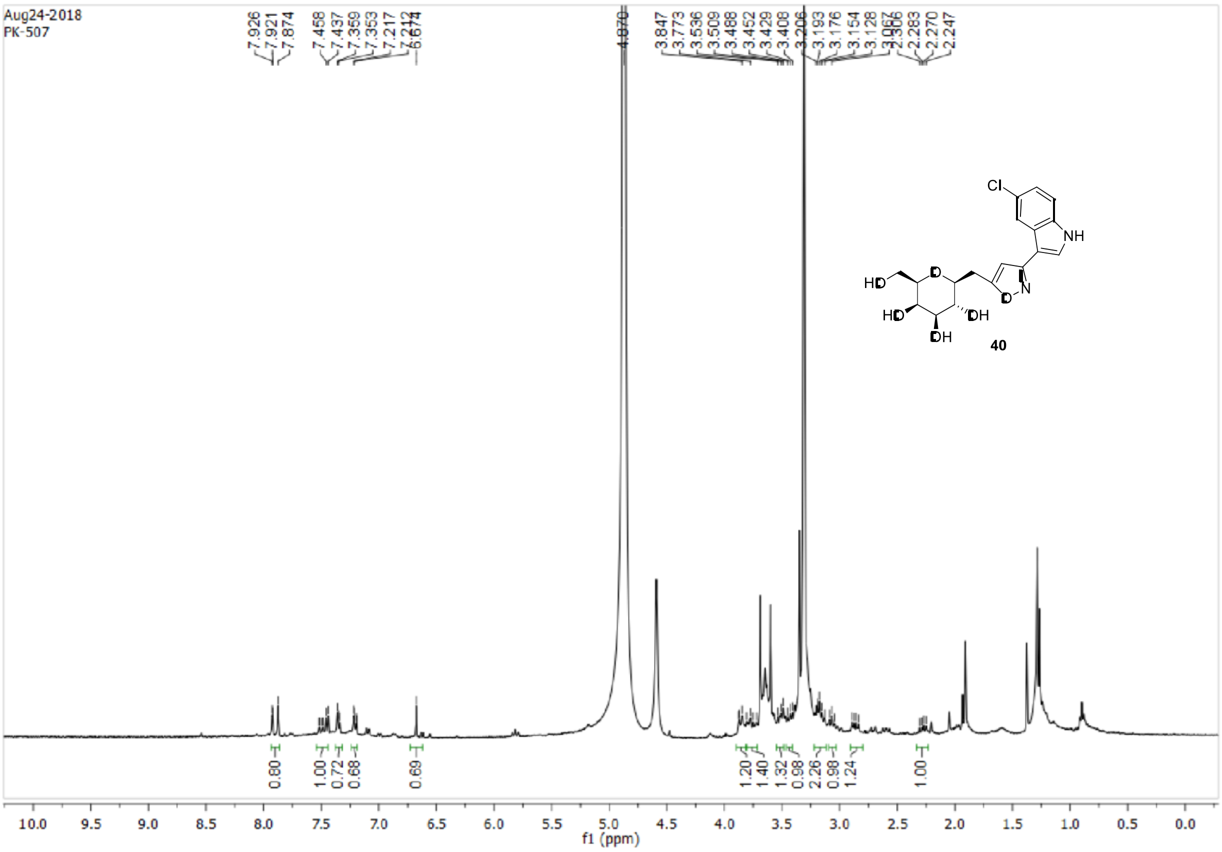
Aug24-2018
PK-507
7.926
7.921
7.874
7.458
7.437
7.359
7.353
7.217
4.870
3.847
3.773
3.536
3.509
3.488
3.452
3.429
3.408
3.206
3.193
3.176
3.154
3.128
2.283
2.270
2.247
Cl
NH
HO
O
N
OH
40
0.80
1.00
0.72
0.68
0.69
1.20
1.40
1.32
0.98
2.26
0.98
1.24
1.00
10.0
9.5
9.0
8.5
8.0
7.5
7.0
6.5
6.0
5.5
5.0
4.5
4.0
3.5
3.0
2.5
2.0
1.5
1.0
0.5
0.0
f1 (ppm)

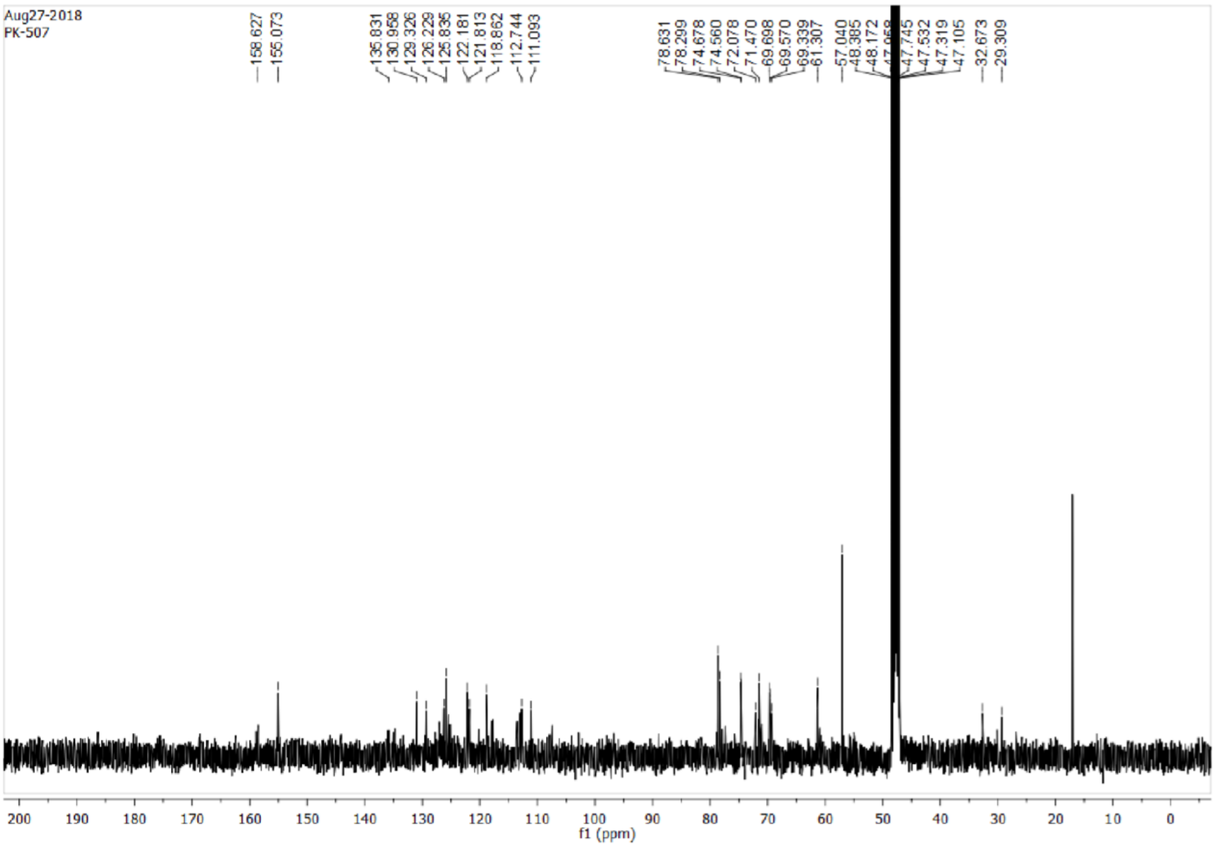
Aug27-2018
PK-507
158.627
155.073
135.831
130.958
129.326
126.229
125.835
122.181
121.813
118.862
112.744
111.093
78.631
78.299
74.678
74.560
72.078
71.470
69.698
69.570
69.339
61.307
57.040
48.385
48.172
47.745
47.532
47.319
47.105
32.673
29.309
200
190
180
170
160
150
140
130
120
110
100
90
80
70
60
50
40
30
20
10
0
f1 (ppm)

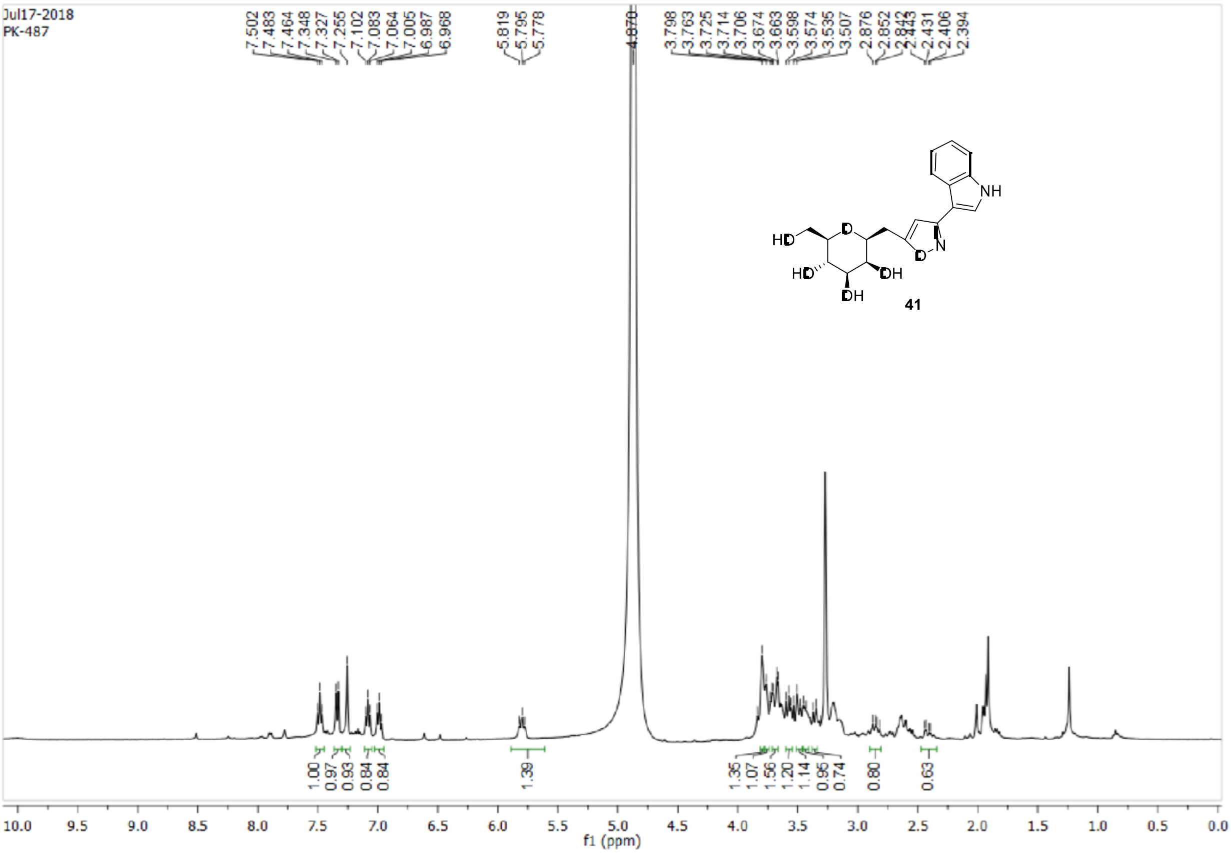
Jul17-2018
PK-487
7.502
7.483
7.464
7.348
7.327
7.255
7.102
7.083
7.064
7.005
6.987
6.968
5.819
5.795
5.778
4.870
3.798
3.763
3.725
3.714
3.706
3.674
3.663
3.598
3.574
3.535
3.507
2.876
2.852
2.431
2.406
2.394
HO
HO
OH
OH
O
O
N
NH
41
1.00
0.97
0.93
0.84
0.84
1.39
1.35
1.07
1.56
1.20
1.14
0.95
0.74
0.80
0.63
f1 (ppm)

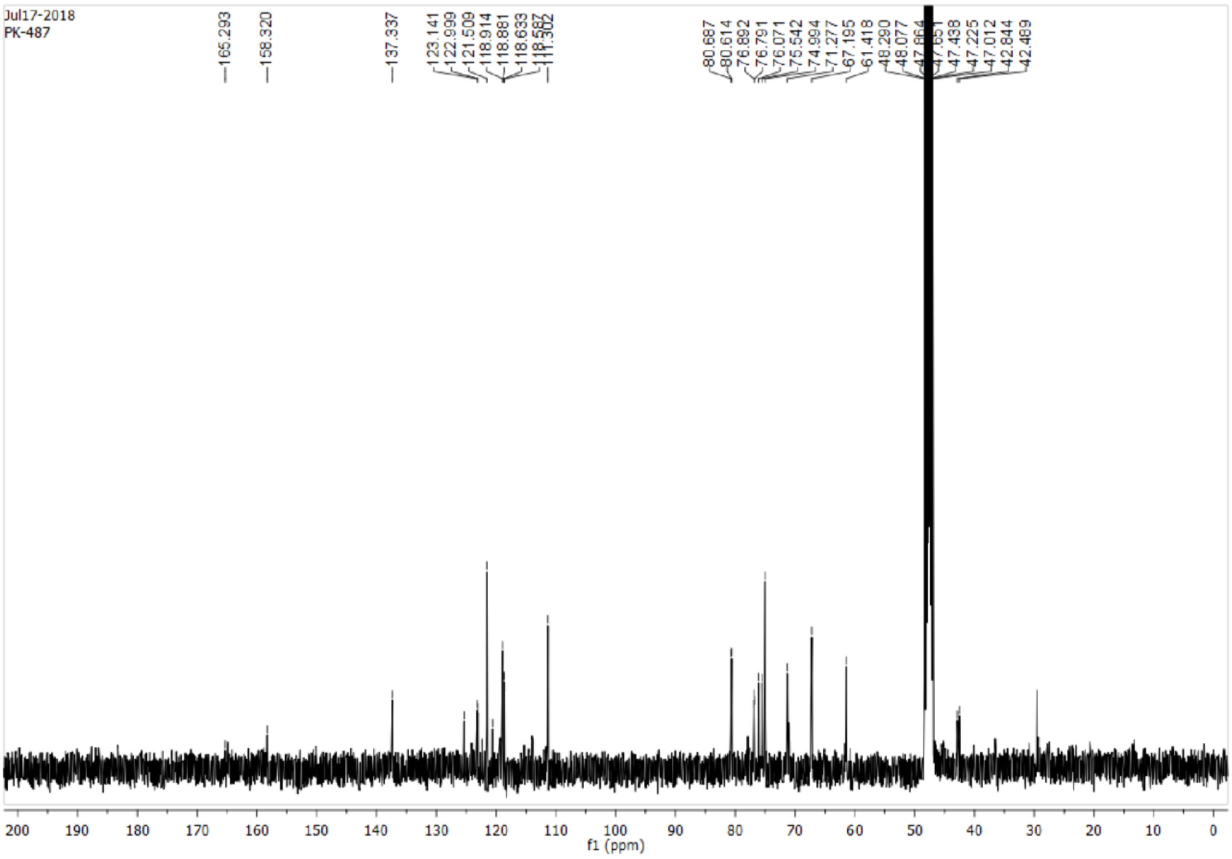
Jul17-2018
PK-487
165.293
158.320
137.337
123.141
122.999
121.509
118.914
118.881
118.633
111.302
80.687
80.614
76.892
76.791
76.071
75.542
74.994
71.277
67.195
61.418
48.290
48.077
47.438
47.225
47.012
42.844
42.489
f1 (ppm)

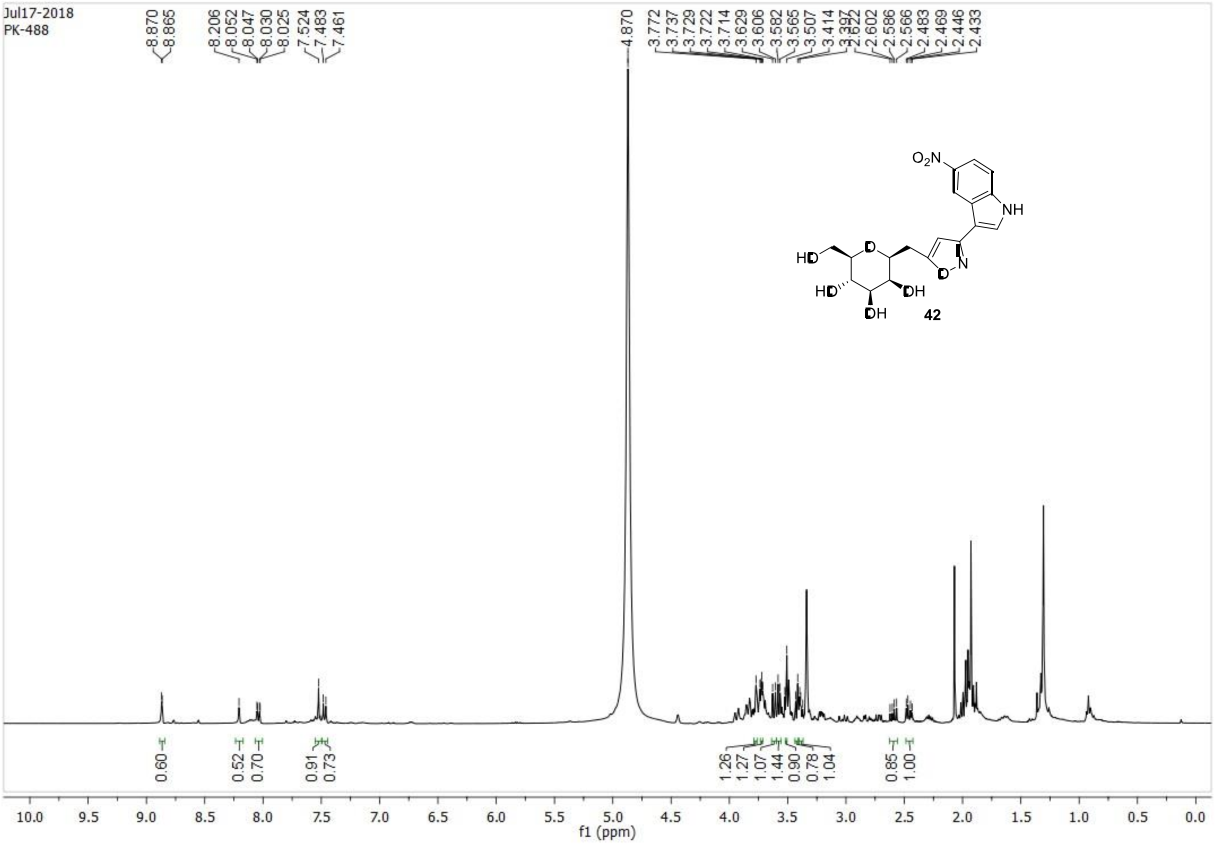
Jul17-2018
PK-488
8.870
8.865
8.206
8.052
8.047
8.030
8.025
7.524
7.483
7.461
4.870
3.772
3.737
3.729
3.722
3.714
3.629
3.606
3.582
3.565
3.507
3.414
2.602
2.586
2.566
2.483
2.469
2.446
2.433
O2N
NH
HO
O
N
OH
42
0.60
0.52
0.70
0.91
0.73
1.26
1.27
1.07
1.44
0.90
0.78
1.04
0.85
1.00
f1 (ppm)

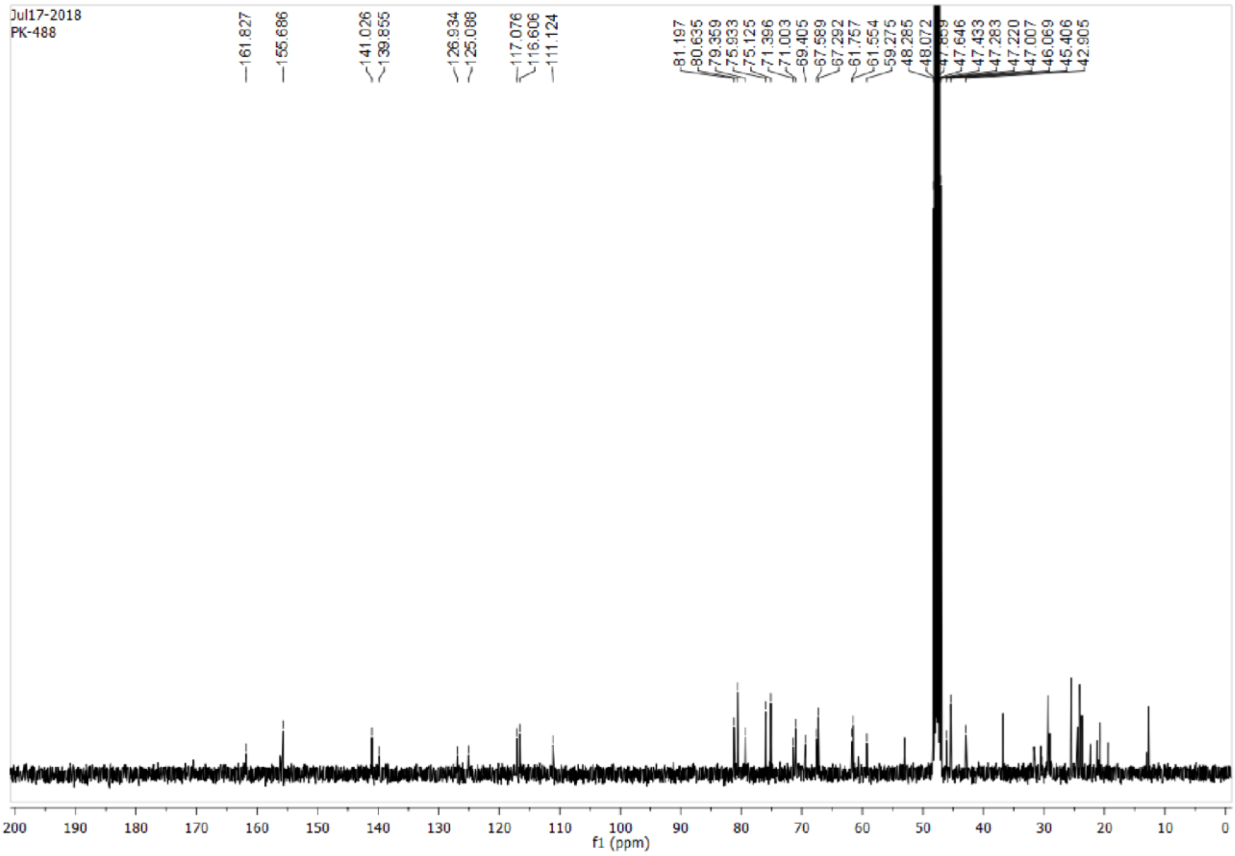
Jul17-2018
PK-488
161.827
155.686
141.026
139.855
126.934
125.088
117.076
116.606
111.124
81.197
80.635
79.359
75.933
75.125
71.396
71.003
69.405
67.589
67.292
61.757
61.554
59.275
48.285
48.072
47.646
47.433
47.283
47.220
47.007
46.069
45.406
42.905
f1 (ppm)

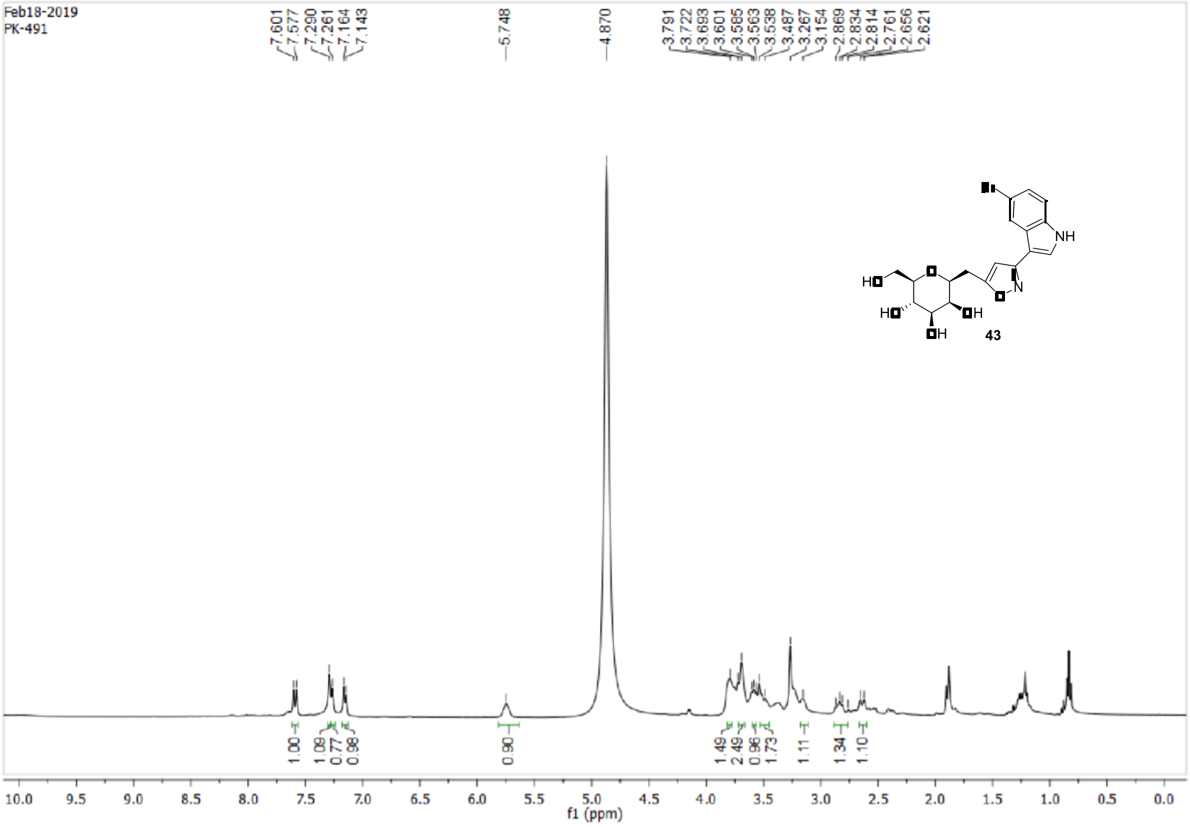
Feb18-2019
PK-491
43
f1 (ppm)

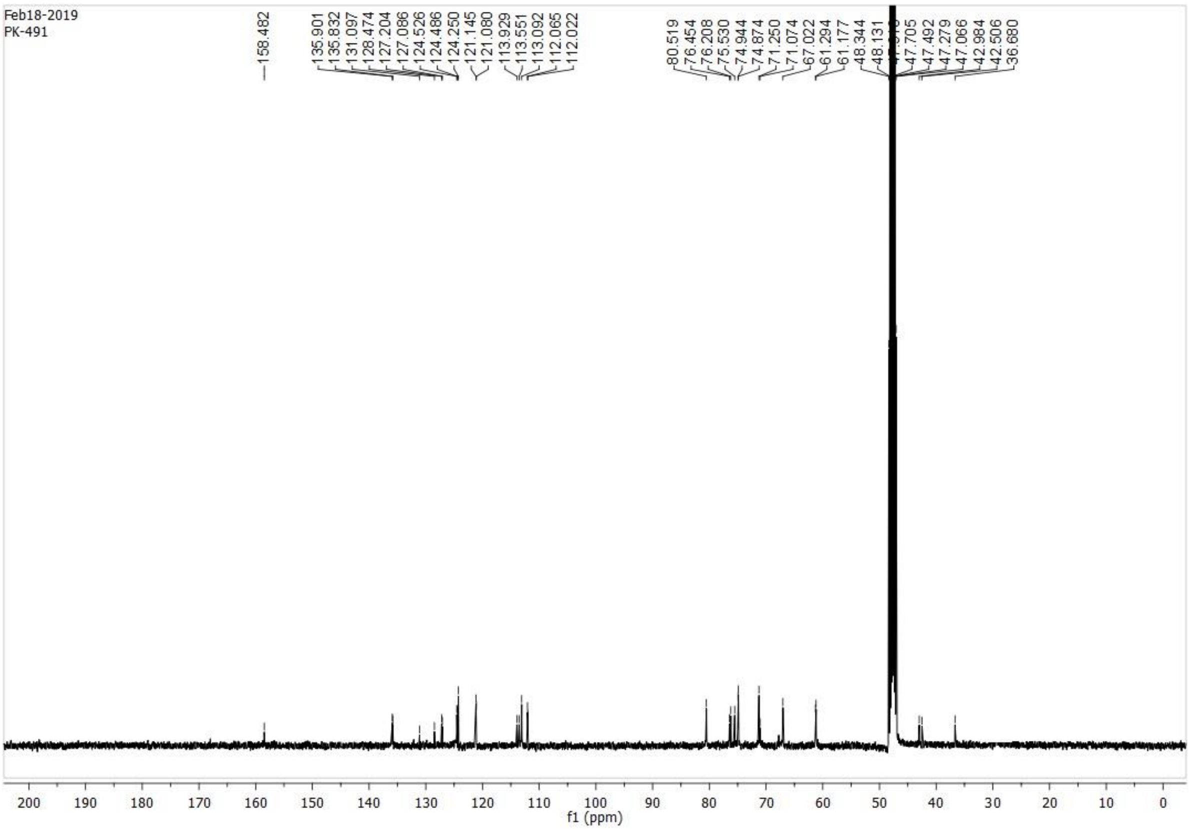
Feb18-2019
PK-491
f1 (ppm)

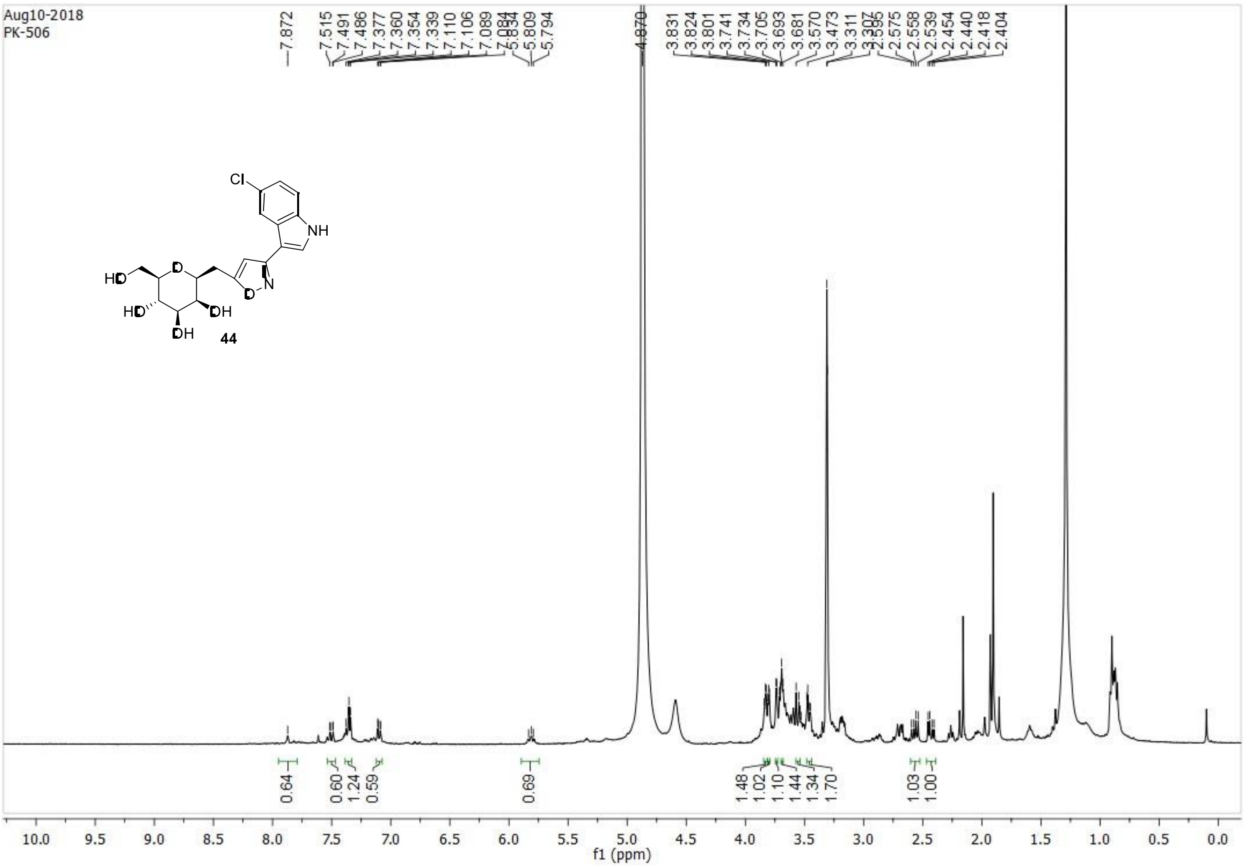
Aug10-2018
PK-506
7.872
7.515
7.491
7.486
7.377
7.360
7.354
7.339
7.110
7.106
7.089
5.809
5.794
4.870
3.831
3.824
3.801
3.741
3.734
3.705
3.693
3.681
3.570
3.473
3.311
2.575
2.558
2.539
2.454
2.440
2.418
2.404
Cl
NH
HO
O
N
OH
44
0.64
0.60
1.24
0.59
0.69
1.48
1.02
1.10
1.44
1.34
1.70
1.03
1.00
f1 (ppm)

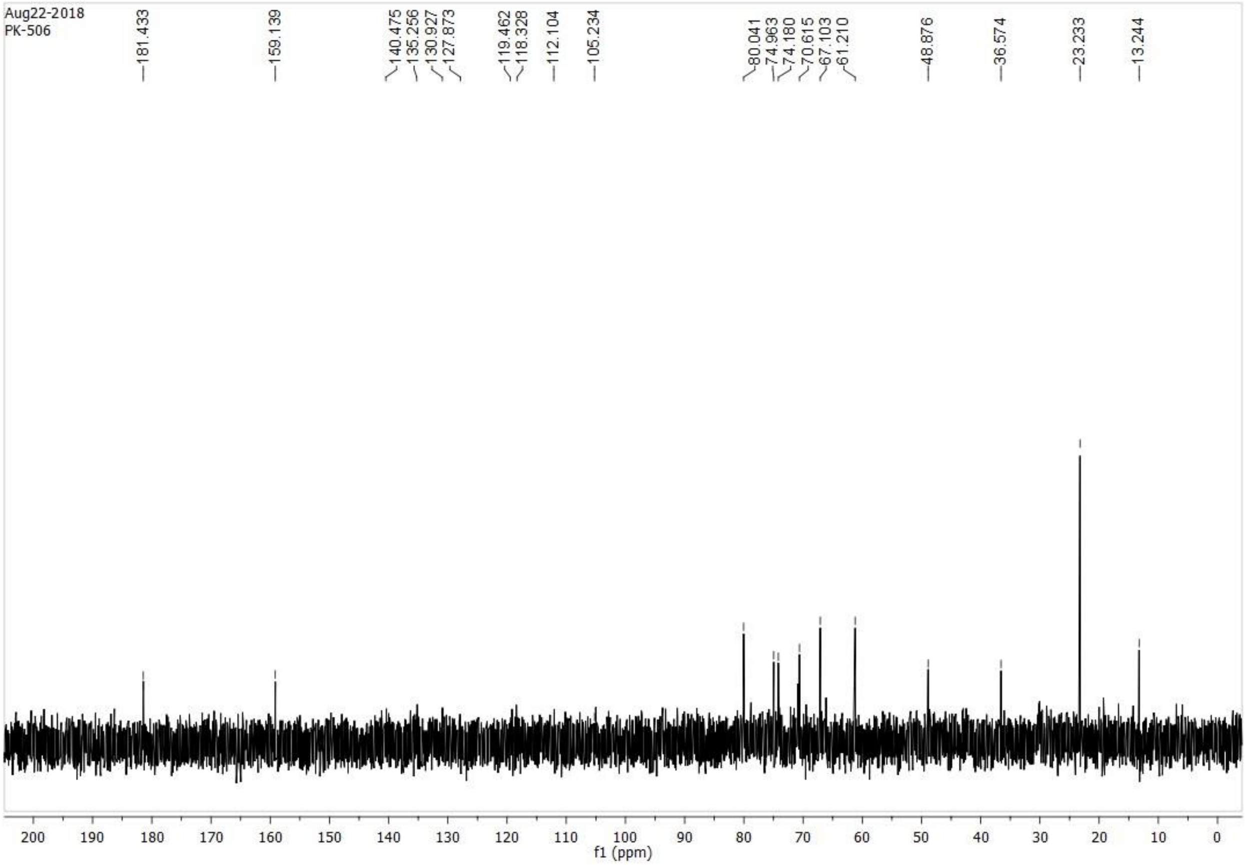
Aug22-2018
PK-506
181.433
159.139
140.475
135.256
130.927
127.873
119.462
118.328
112.104
105.234
80.041
74.963
74.180
70.615
67.103
61.210
48.876
36.574
23.233
13.244
f1 (ppm)

Lactate Dehydrogenase Cell Assay Materials & Methods: (Himedia, product code; CCK036):

Introduction:

Lactate dehydrogenase (LDH) is a cytoplasmic enzyme present in all mammalian cells. Plasma membrane of live cells is impermeable to LDH. However it is rapidly released into the culture medium when plasma membrane is damaged. The quantity of the leaked LDH is used as a measure of cell cytotoxicity. Due to low molecular weight (35kDa) LDH is easily released from the cells even with very minor damage or perturbations in the membrane. This makes the LDH release assay very sensitive.

This Kit has been designed for rapid determination of cell cytotoxicity using an enzyme coupled two step reaction.

Lactate Dehydrogenase (LDH) is based on an enzyme coupled reaction in which the LDH released from cells with damaged membrane reduces the tetrazolium dye to a colored formazan product. The reaction takes place in two steps - Step 1:

LDH catalyzes the conversion of lactate to pyruvate via reduction of NAD+ to NADH.

Step 2:

NADH thus generated reduces the tetrazolium dye to a colored formazan product which can be measured spectrophotometrically.

Materials:

- Kit contents: CCK036(A) LDH reagent 5 x 5ml -20 ºC in dark CCK036(B) Lysis solution 5ml -20 ºC in dark
  CCK036(C) Stop solution 25ml -20 ºC in dark

- Cells in appropriate medium
- Adjustable pipettes and a repeat pipettor
- 96-well plate for culturing the cells
- 96-well plate reader capable of measuring the Absorbance at 450nm and more than 600 nm.

Method:

Assay controls

It is necessary to include different types of controls depending on application of LDH assay.

1. Maximum LDH control: It is recommended to perform maximum LDH estimation to determine total amount of LDH present in the cells.
2. Background control: Significant amount of LDH is present in the serum used for supplementing the culture medium. This contributes to the background absorbance / fluorescence. Hence it is recommended to set a background control that contains medium without cells. Absorbance/fluorescence of background control wells should be subtracted from absorbance / fluorescence values of experimental wells to get corrected
3. Untreated control: Untreated control, also known as vehicle control should be included in the assay to obtain LDH values generated due to the solvent in which test compound is

dissolved. Untreated control wells contain the solvent at a concentration used for dissolving the test compound.

- Preparation of cells: Freshly harvested MCF-7 cells for assay. Seeded in the 96well plate (1500/well) incubated at 37^oC in a 5% CO2 incubator for 8hrs allow the cells to attach bottom of plate.
- Media removed and 90ul of fresh media was added in each well with compound (25uM in treated wells in triplicate) for 48hrs.
- Meanwhile lysis solution CCK036(B) was thawed and equilibrate it at room temperature.
- 10µl of lysis solution CCK036 (B) was added to maximum LDH control wells, similarly 10µl DPBS was added to experimental wells, untreated control wells and vehicle control wells.
- Plate was Incubated at 37 ^oC with 5% CO2 for 30 – 45 minutes.
- Observed cell lysis in maximum LDH control cells microscopically.
- Plate was Centrifuge at 1500 rpm for 5 minutes to settle the cell debris at the bottom of wells.
- 50µl of supernatant was transfer from each well to new 96well plate.
- LDH reagent bottle CCK036 (A) was thawed and equilibrated at room temperature. 50µl of LDH reagent was added to each well and kept the plate at room temperature for 15 – 30 minutes. (*Note: Wrap the plate in aluminium foil to avoid exposure to light.)*
- 50 µl of Stop solution CCK036 (C) was added to each well and read the absorbance at 450nm as a main wavelength and reference wavelength greater than 600nm.
- Average absorbance values were calculated from triplicate values.

LDH generated due to effect of test compound on the cells

A = Avg absorbance of test – Avg absorbance of background control (or vehicle control if applicable)

Total LDH released from the cells due to complete lysis

B = Avg absorbance of maximum LDH control – Avg absorbance of volume correction control

Baseline LDH released from the cells

C = Avg absorbance of untreated control – Avg absorbance of background control

% Cytotoxicity $= \frac{A-C}{B-C}$ 100 x

Results:

											**%Cytotoxicity**
					**Avrg**	**A**	**B**	**C**	**A-C**	**B-C**	**100*A-C/B-C**
**9**	0.261	0.243	0.236	0.241	0.24525	0.1025			0.04725		12.90102389

**20**	0.296	0.196	0.209	0.205	0.2265	0.08375			0.0285		7.781569966
**25**	0.279	0.291	0.266	0.276	0.278	0.13525			0.08		21.84300341
**34**	0.34	0.291	0.299	0.319	0.31225	0.1695			0.11425		31.19453925
**37**	0.351	0.332	0.327	0.315	0.33125	0.1885			0.13325		36.38225256
**UTC**	0.211	0.191	0.18	0.21	0.198			0.05525			
**MLC**	0.684	0.5	0.521	0.552	0.56425		0.4215			0.36625	
**BLNK**	0.14	0.143	0.144	0.144	0.14275						

UTC= Untreated control
MLC= Maximum LDH control
BLNK= Blank (Background control)

Table S1. Table represent the hydrogen bonding interactions of active compounds (9, 20, 25, 34, 37) with the Cox-2 (1CX2).

S. No.	Name of the protein (PDB ID)	Ligand (Active Compound)	Number of hydrogen bond	Hydrogen bonding amino acid	Bond Length (Å)
1.	Cox – 2 (1CX2)	**9**	5	Asn43<br>Asn43<br>Lys468<br>Ser471<br>Tyr122	1.9<br>2.7<br>2.5<br>2.6<br>2.0
2.	Cox – 2 (1CX2)	**20**	5	His214<br>Gln454<br>His386<br>His386<br>Asn382	1.8<br>2.8<br>2.3<br>2.9<br>2.6
3.	Cox – 2 (1CX2)	**34**	3	Asn43<br>Asn39<br>Gln461	2.7<br>2.1<br>2.0
4.	Cox – 2 (1CX2)	**25**	4	Cys41<br>Glu465<br>Cys47<br>Arg44	3.0<br>3.4<br>2.5<br>2.6
5.	Cox – 2 (1CX2)	**37**	5	Arg120<br>Glu524<br>Lys83<br>Lys83<br>Pro84	1.9<br>2.4<br>2.3<br>1.8<br>2.5
